# Ligand-Unsupported Assembly of a Linear Penta-Tin Chain via Metal–Metal Donor–Acceptor Interactions

**DOI:** 10.1021/acs.inorgchem.6c00602

**Published:** 2026-06-23

**Authors:** Yi-Chen Lin, Kuheli Das, Ankit Raj, Li-Ching Shen, You-Song Cheng, Ting-Shen Kuo, Han-Jung Li, Hirotsugu Hiramatsu, Sung-Fu Hung, Hsueh-Ju Liu

**Affiliations:** † Department of Applied Chemistry, 34914 National Yang Ming Chiao Tung University, 1001 Daxue Rd, East District, Hsinchu City 300093, Taiwan; ‡ Department of Chemistry, 34879 National Taiwan Normal University, Taipei 11677, Taiwan; § Department of Chemistry, 34900 Chung Yuan Christian University, Taoyuan City 320314, Taiwan; ∥ Center for Emergent Functional Matter Science, National Yang Ming Chiao Tung University, 1001 Daxue Rd, East District, Hsinchu City 300093, Taiwan

## Abstract

Herein, we report a metal–metal interaction–driven strategy for constructing extended tin chains without relying on preorganized ligand frameworks. By combining a nucleophilic Sn­(I) distannyne supported by a sterically undemanding dipyridylpyrrolate ligand with a neutral yet highly Lewis-acidic Sn­(II) synthon, stepwise chain growth from di- to tri- and ultimately to a linear penta-tin complex was achieved. Multinuclear NMR, UV–vis, Raman, and X-ray absorption spectroscopies, together with elemental analysis, establish the structures and mixed-valence nature of the tri- and penta-tin complexes. Complementary density functional theory (DFT) calculations support these assignments by identifying the centrally located Sn­(II) motif as the thermodynamically preferred arrangement and rationalizing the observed spectroscopic trends.

## Graphical abstract

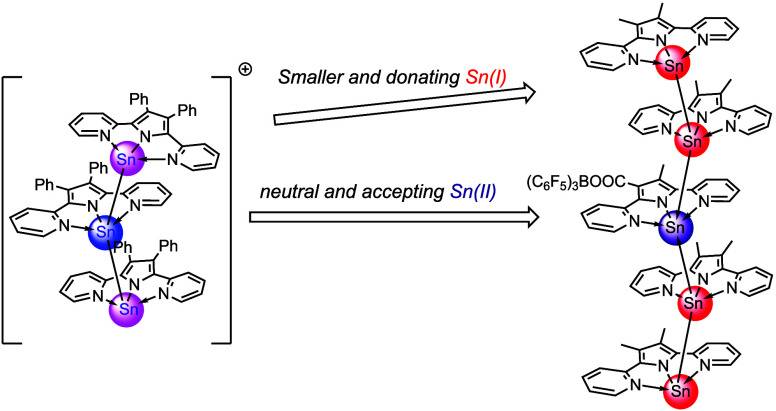

## Introduction

Extended metal atom chains (EMACs), also known as metal strings and originally coined by Cotton^1,2^ and Peng,^3,4^ are multimetallic frameworks featuring direct metal–metal bonding. Owing to electronic delocalization along metal–metal backbones, EMAC-type architectures may display distinctive conductive and magnetic properties, motivating interest in molecular electronics, molecular wires, and single-molecule magnets.^5–7^ Traditionally, EMAC synthesis has relied on the design—and often synthetically demanding preparation—of preorganized multidentate ligands, which, once obtained, readily enforce linear metal alignment and facilitate chain assembly. Representative examples include Peng’s 11-nickel chain supported by naphthyridine–amido linkers^8^ and Tsai’s cyclic octagermanium complex stabilized by long-chain multidentate ligands.^9,10^ Alternative approaches exploiting intrinsic metal–metal interactions, such as cupriphilicity in copper assemblies by Tsai and co-workers^11^ or donor–acceptor interactions in Pt­(II)–Tl­(I) chains by Martín, Moreno and co-workers,^12^ remain comparatively rare.

In the main-group domain, group 14 elements are well-known for forming catenated E–E chains (E = Si, Ge, Sn), as exemplified by polysilanes, polygermanes, and polystannanes.^13,14^ Beyond these classical tetravalent systems, low-valent group 14 species offer access to discrete, molecule-like metal chains owing to their ambiphilic character, arising from the coexistence of a stereochemically active lone pair and an empty *p* orbital. This donor–acceptor electronic structure underlies heavy alkene analogues, including trans-bent E = E motifs, and has enabled mixed-valence germanium assemblies such as Piskunov’s σ-bonded Ge­(I)/Ge­(II) tetranuclear complex^15^ and Aldridge’s tetra-germanium system featuring a central Ge = Ge unit.^16^ In tin chemistry, pincer-type ligands have stabilized Sn­(I) dimers featuring single Sn–Sn bonds,^17–19^ which readily coordinate Lewis acids (e.g., Fe­(CO)_4_ and W­(CO)_5_) through Sn → M interactions.^20,21^ These distannynes therefore represent promising building blocks for extended metal chains. Very recently, Shen et al. reported a rare mixed-valence tetratin chain featuring a diatomic Sn(0)–Sn(0) unit, stabilized by a double-layered N–P ligand, N­{CH_2_CH_2_NHP^i^Pr_2_}_3_. In this system, the nitrogen donors act as hard bases that coordinate to Sn­(III), while the softer phosphorus donors bind to Sn(0), giving rise to an extended tetratin framework of the type Sn­(III)–Sn(0)–Sn(0)–Sn­(III).^22^

Previously, our group reported mixed-valence, homocatenated tri-tin complexes (L^Ph^Sn)_3_X supported by planar dipyridylpyrrolate ligands L^Ph^ (Figure 1),^23,24^ formed via dehydrocoupling of a transient tin­(II) hydride and subsequent Sn–Sn bond formation with Lewis-acidic Sn­(II) centers.^25^ π–π interactions between planar ligand frameworks were identified as important stabilizing factors for the trinuclear structures. However, extension beyond three tin centers proved challenging likely due to steric congestion from phenyl substituents and the reduced donor strength of terminal Sn atoms within the cationic (L^Ph^Sn)_3_^+^ framework. To overcome these limitations, we sought an alternative strategy that does not rely on preorganized multidentate ligands, but instead exploits intrinsic metal–metal donor–acceptor interactions to control chain growth. Accordingly, we designed a less sterically demanding methyl-substituted ligand (L^Me^, see Figure 1a) to enhance the nucleophilicity of Sn­(I) building blocks, together with a zwitterionic, dianionic ligand framework (L^BArF^, Figure 1) capable of supporting a highly Lewis-acidic Sn­(II) center while maintaining overall charge neutrality.

**Figure 1:**
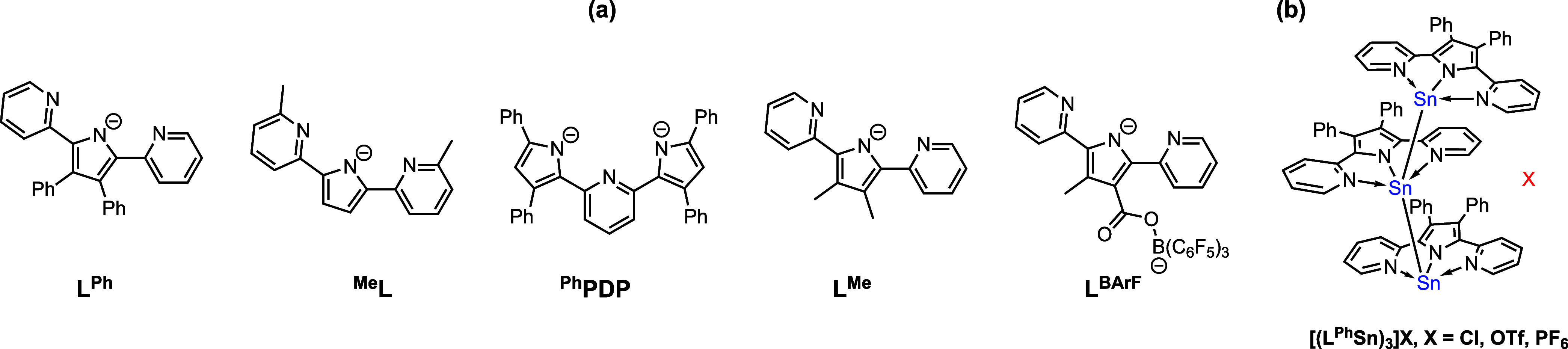
(a) NNN pincer-type ligands employed in our previous studies and in the present work; (b) the tri-tin complexes supported by L^Ph^ ligands.

Herein, we report the synthesis of a linear mixed-valence penta-tin complex featuring four consecutive Sn–Sn bonds, representing the longest ligand-unsupported tin chain reported to date. In contrast to transition-metal EMACs that rely on bridging ligands,^23^ this system constitutes a rare main-group analogue with a fully homocatenated Sn–Sn–Sn–Sn–Sn backbone. Its solution behavior, spectroscopic characterization, and formation pathway are described, together with related di- and tri-tin intermediates that illuminate how controlled metal–metal interactions can be harnessed to construct extended tin chains.

## Results and Discussion

### Synthesis and Structural Characterization of L^Me^Sn­(II)­Cl and Distannyne L^Me^Sn­(I)–Sn­(I)­L^Me^ (Sn_2_)

At room temperature, equimolar amounts of **L** ^**Me**^ **H** ^26^ and Sn­[N­(SiMe_3_)_2_]Cl (Sn­(HMDS)­Cl) were combined in diethyl ether, rapidly affording **L** ^**Me**^ **SnCl** as a yellow precipitate in 94% yield. The product was isolated as an air- and moisture-sensitive solid and characterized by multinuclear NMR spectroscopy. Reduction of **L** ^**Me**^ **SnCl** with one equivalent of K­[BH^S^Bu_3_] in THF resulted in a rapid color change from pale yellow to green, indicative of transient formation of L^Me^SnH, which gradually converted at room temperature into the distannyne L^Me^Sn–SnL^Me^ (abbreviated as “**Sn**_**2**_”), isolated as dark green crystals in 82% yield (Scheme 1). This reaction pathway closely parallels those previously reported for L^Ph^Sn–SnL^Ph^ and ^Me^LSn–Sn^Me^L.^25^ The ^119^Sn NMR resonance of **L** ^**Me**^ **SnCl** appears at δ = −356.3 ppm, while that of L^Me^Sn–SnL^Me^ (**Sn**_**2**_) is observed at δ = −71.1 ppm, consistent with reduction from Sn­(II) to Sn­(I). This downfield shift mirrors trends observed for related ligand systems, including L^Ph^SnCl/L^Ph^Sn–SnL^Ph^ and ^Me^LSn derivatives, and reflects the increased electron density at tin upon Sn–Sn bond formation (see Figure 2).^23–25^ Notably, the presence of electron-donating methyl substituents in the L^Me^ ligand framework shifts the ^119^Sn resonances further downfield relative to phenyl-substituted analogues, consistent with the enhanced σ-donor character of the L^Me^ ligand.

**Scheme 1:**
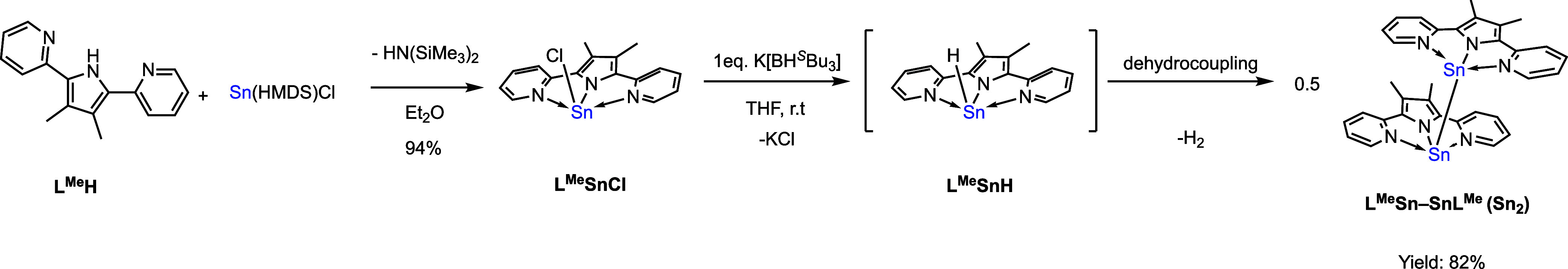
Synthesis of L^Me^SnCl and L^Me^Sn–SnL^Me^ (**Sn**_**2**_)

**Figure 2:**
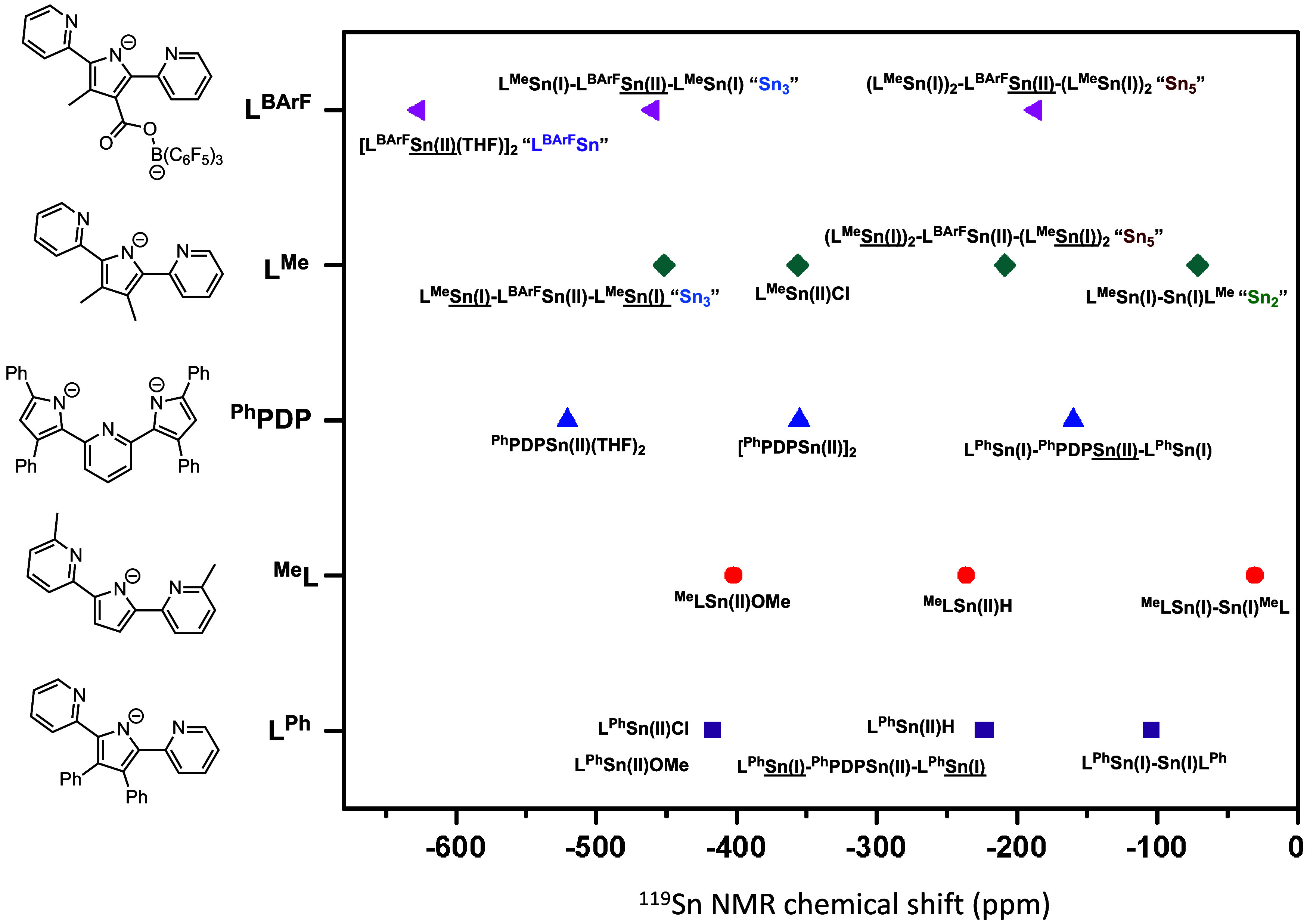
^119^Sn NMR chemical shifts of NNN pincer-supported tin complexes from our previous studies and the present work.

Single-crystal X-ray diffraction analysis of **L** ^**Me**^ **SnCl** (Figure 3a) confirms a four-coordinate Sn­(II) center supported by the dipyridylpyrrolate ligand. The observed bond lengths and angles closely match those of related complexes, including L^#^SnCl (L^#^ = 2,5-bis­{(pyrrolidino)­methyl}­pyrrolate)^27^ and L^Ph^SnCl,^23^ indicating that substitution of phenyl groups with methyl substituents does not significantly perturb the primary coordination environment around the tin center. In addition, L^Me^Sn–SnL^Me^ (**Sn**_**2**_) adopts a closely stacked double-decker structure, characterized by a small interplanar angle of 11.8° between the ligand planes and a Sn–Sn bond length of 2.954(4) Å, consistent with a single Sn–Sn bond and comparable to those reported for singly bonded distannynes in the literature (2.8981(9)–3.0486(6) Å, see Figure 3b).^18^ This nearly parallel arrangement, enabled by the reduced steric demand of the L^Me^ ligand, promotes interligand proximity and supports our design strategy toward the construction of extended polytin chain architectures.

**Figure 3:**
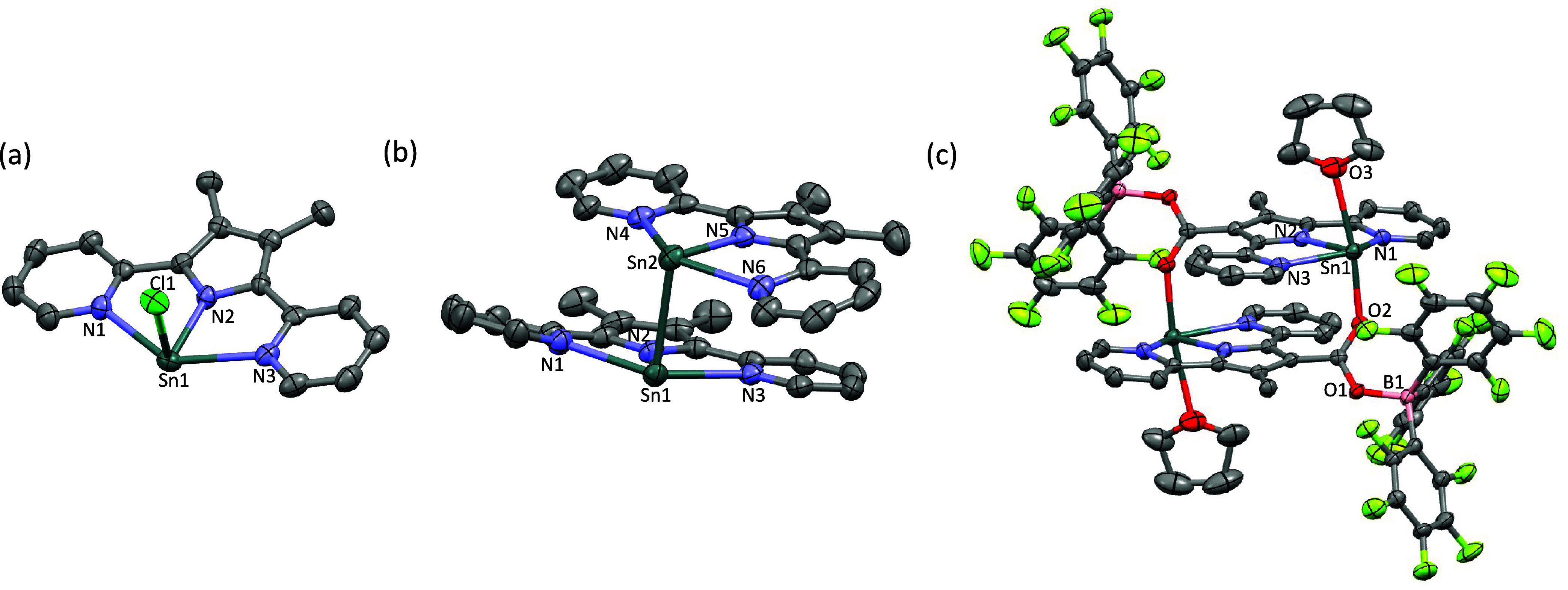
X-ray structures (30% probability ellipsoids) of (a) L^Me^SnCl, (b) L^Me^Sn–SnL^Me^ (**Sn**_**2**_), and (c) **[L** ^**BArF**^ **Sn­(THF)]**_**2**_. H atoms have been omitted for clarity.

### Synthesis and Structural Analysis of the **L^BArF^** Ligand and **[L** ^**BArF**^ **Sn­(THF)]**_**2**_

To access a charge-neutral yet strongly Lewis-acidic Sn­(II) synthon suitable for chain extension, we sought to develop a dianionic ligand framework based on the dipyridylpyrrolate scaffold. Incorporation of a carboxylate functionality was initially targeted to introduce an additional anionic donor site. Acid-catalyzed hydrolysis of the ester-functionalized ligand **L** ^**Me,COOMe**^ **H** ^26^ unexpectedly resulted in decarboxylation, yielding **L** ^**Me,H**^ **H** as the major product (66% yield). We attribute this outcome to initial ester hydrolysis followed by thermally induced decarboxylation under acidic conditions. In contrast, base-promoted hydrolysis of **L** ^**Me,COOMe**^ **H**, followed by neutralization, successfully afforded the desired carboxylic acid **L** ^**Me,COOH**^ **H** in 64% yield (Scheme 2). Subsequent treatment of **L** ^**Me,COOH**^ **H** with Sn­(HMDS)_2_ led to immediate precipitation of an insoluble yellow solid, suggestive of polymeric species possibly formed through bridging carboxylate–Sn interactions. To suppress such aggregation, the carboxylate functionality was capped using a suitable Lewis acid. Initial attempts using Si­(cat)_2_ (cat = catecholate) resulted in complex product mixtures. In contrast, reaction of **L** ^**Me,COOH**^ **H** with tris­(pentafluorophenyl)­borane in toluene cleanly afforded L^BArF^H_2_ as a fluorescent yellow solid in 95% yield after recrystallization from pentane (Scheme 2). Single-crystal X-ray diffraction of **L** ^**BArF**^ **H**_**2**_ revealed coordination of the B­(C_6_F_5_)_3_ fragment to one oxygen atom of the carboxyl group, while one pyridyl nitrogen engages in intramolecular hydrogen bonding with the acidic proton. This structural motif confirms the strong preference of B­(C_6_F_5_)_3_ for oxygen coordination and provides effective protection against carboxylate bridging (see Figure S29 for the crystal structure).

**Scheme 2:**
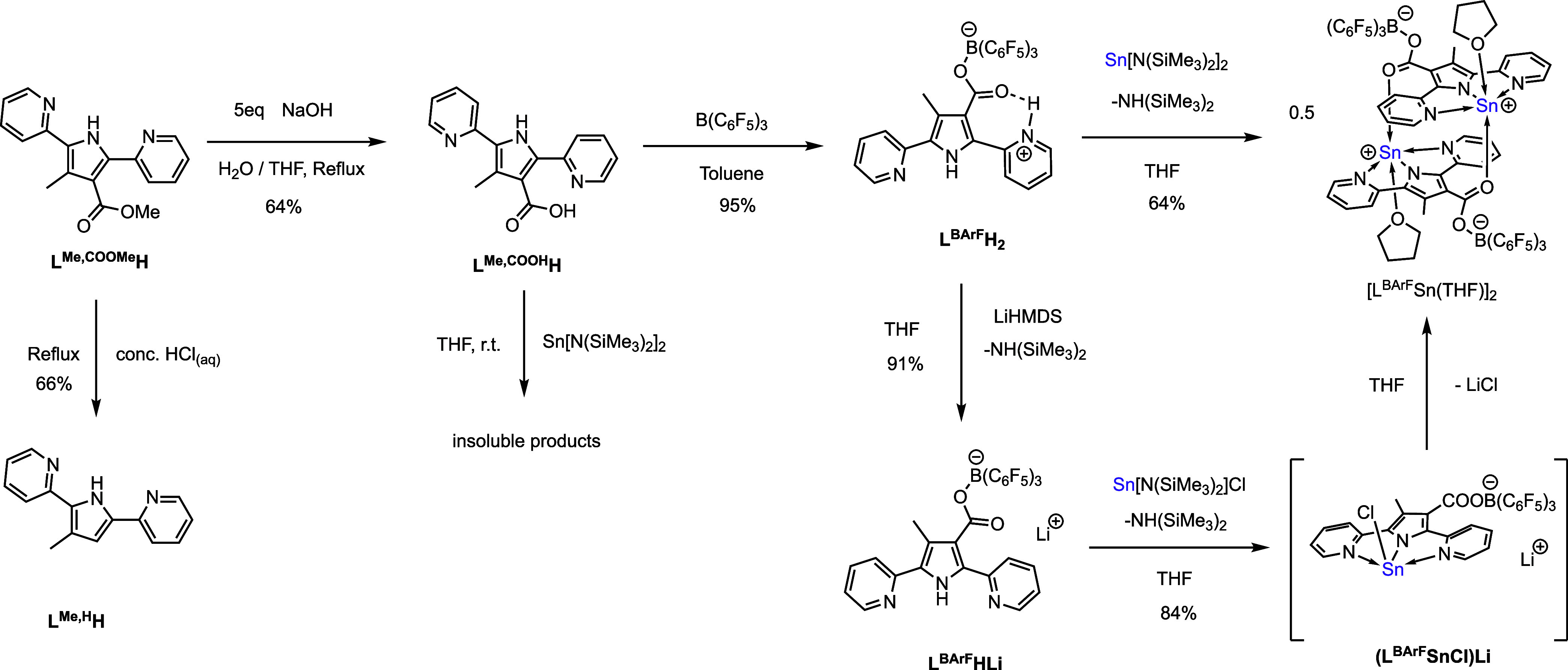
Synthesis of the **L^BArF^** Ligand and **[L** ^**BArF**^ **Sn­(THF)]**_**2**_

Metalation of **L** ^**BArF**^ **H**_**2**_ with Sn­[N­(SiMe_3_)_2_]_2_ in THF resulted in gradual color change from fluorescent yellow to yellow, yielding **[L** ^**BArF**^ **Sn­(THF)]**_**2**_ in 64% yield after crystallization (Scheme 2). An alternative route involving deprotonation of L^BArF^H_2_ with Li­[N­(SiMe_3_)_2_] (LiHMDS) followed by metathesis with Sn­[N­(SiMe_3_)_2_]Cl afforded the same product after elimination of LiCl. Single-crystal X-ray diffraction analysis shows that **[L** ^**BArF**^ **Sn­(THF)]**_**2**_ exists as a dimeric species featuring five-coordinate Sn­(II) centers (Figure 3c). Each tin atom adopts a distorted trigonal-bipyramidal geometry (τ ≈ 0.6), with the dipyridylpyrrolate ligand occupying the equatorial plane and axial coordination by a THF molecule and a carboxyl oxygen atom from the opposing ligand. Notably, the two planar dipyridylpyrrolate frameworks are arranged in a parallel fashion with an interplanar distance of 3.35 Å, indicative of π–π interactions that further stabilize the dimeric structure. Further insight is gained from comparison of the solvent-free dimer (L^BArF^Sn)_2_, crystallized from toluene (Figure S31), with its THF adduct **[L** ^**BArF**^ **Sn­(THF)]**_**2**_. Upon THF coordination, the Sn–N­(pyrrole) (2.137(3) vs 2.145 Å) and Sn–O­(carboxylate) (2.276(2) vs 2.3059(18) Å) bond lengths increase, whereas the Sn–N­(pyridine) distances (2.432(3)–2.479(3) vs 2.425(2)–2.479(2) Å) remain essentially unchanged. The Sn···Sn separation decreases slightly (6.725 to 6.711 Å). These structural changes indicate a weakening of internal Sn–ligand interactions upon coordination of the external THF donor. The ^119^Sn NMR spectrum of **[L** ^**BArF**^ **Sn­(THF)]**_**2**_ exhibits a resonance at δ = −626.1 ppm, significantly upfield-shifted relative to the related five-coordinate Sn­(II) complex ^Ph^PDPSn­(THF)_2_ (δ = −520.4 ppm).^24^ This shift reflects the unique electronic structure of the L^BArF^ ligand, which combines a {NNN} donor pocket that coordinates to the Sn­(II) center with a charge-separated framework incorporating a B­(C_6_F_5_)_3_-stabilized carboxylate. As a result, the Sn­(II) center remains significantly electron-deficient, consistent with its pronounced Lewis acidity (see Figure 2). With the nucleophilic Sn­(I) building block L^Me^Sn–SnL^Me^ (**Sn**_**2**_) and the neutral yet strongly Lewis-acidic Sn­(II) synthon **[L** ^**BArF**^ **Sn­(THF)]**_**2**_ in hand, we next explored their reactivity toward controlled metal–metal chain extension.

Initial attempts to access a tetra-tin complex via reaction of (L^Ph^Sn)_3_Cl with **[L** ^**BArF**^ **Sn­(THF)]**_**2**_ were unsuccessful, despite the presence of terminal tin centers in the cationic (L^Ph^Sn)_3_^+^ framework that, in principle, bear lone pairs capable of participating in chain-extension reactions. Instead, the reaction reproducibly afforded an ionic species formulated as [(L^Ph^Sn)_3_]­[L^BArF^SnCl], in which the trinuclear tin cation remains intact while the L^BArF^Sn fragment sequesters the chloride counterion. Owing to limited characterization data and the poor quality of the available crystallographic information, details of this species are provided in the Supporting Information (Scheme S1 and Figure S30), and it is discussed here only insofar as it highlights the intrinsic limitations of the L^Ph^-based system for further chain extension. This outcome likely arises from two factors: (i) the diminished donor strength of the terminal tin atoms within the cationic (L^Ph^Sn)_3_^+^ core, and (ii) steric congestion imposed by the phenyl substituents, which disfavors further coordination to the L^BArF^Sn unit. Collectively, these results underscore the limitations of extending the L^Ph^-based system through metal–metal interactions alone.

### Synthesis of Tri-Tin (**Sn**_**3**_) and Penta-Tin (**Sn**_**5**_) Complexes

We therefore turned to the less sterically encumbered and more nucleophilic distannyne **Sn**_**2**_. Addition of 0.5 equiv of **[L** ^**BArF**^ **Sn­(THF)]**_**2**_ to a THF solution of **Sn**_**2**_ resulted in an immediate color change from dark green to dark red, closely resembling the color observed for tri-tin species in related systems. After stirring at room temperature for 1 h, recrystallization from *n*-pentane afforded the trinuclear complex L^Me^Sn–L^BArF^Sn–L^Me^Sn (abbreviated as **Sn**_**3**_) as a dark red solid in 89% yield (Scheme 3). Encouraged by this result, we investigated whether further chain extension could be achieved by increasing the amount of Sn­(I) building block. Treatment of two equivalents of **Sn**_**2**_ with 0.5 equiv of **[L** ^**BArF**^ **Sn­(THF)]**_**2**_ in THF produced a rapid color change to dark brown. Workup and recrystallization from pentane yielded the penta-tin complex (L^Me^Sn)_2_–L^BArF^Sn–(L^Me^Sn)_2_ (abbreviated as **Sn**_**5**_) as a black solid in 85% yield. Consistent with this formulation, sequential addition of **Sn**_**2**_ to a preformed THF solution of the trinuclear complex **Sn**_**3**_ resulted in the same dark brown coloration and identical spectroscopic features, indicating formation of the same penta-tin product **Sn**_**5**_.

**Scheme 3:**
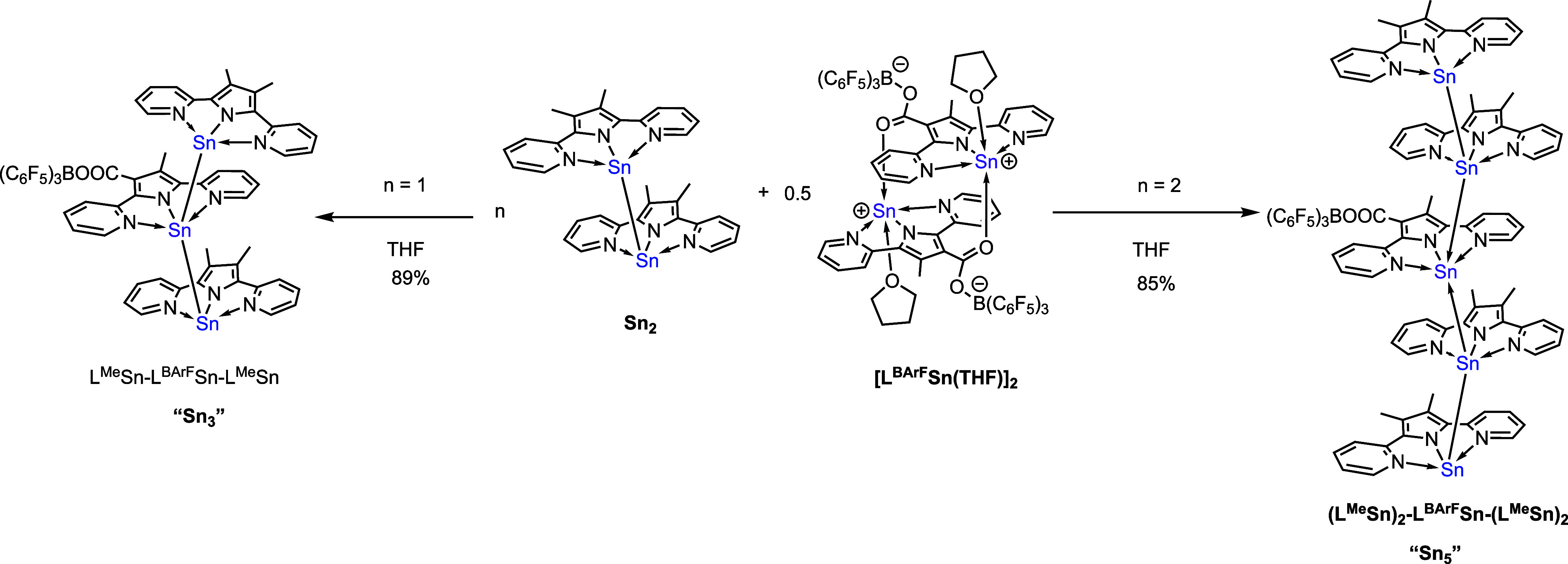
Synthesis of the Tri-Tin L^Me^Sn–L^BArF^Sn–L^Me^Sn (**Sn**_**3**_) and Penta-Tin (L^Me^Sn)_2_–L^BArF^Sn–(L^Me^Sn)_2_ (**Sn**_**5**_) Complexes

Despite extensive crystallization efforts, suitable single crystals of the tri- and penta-tin complexes could not be obtained for X-ray diffraction analysis. Accordingly, their structures were established on the basis of a complementary set of multinuclear NMR, UV–vis, Raman, and X-ray absorption spectroscopies together with elemental analysis, which collectively provide a consistent and internally corroborated picture of their molecular structures.

### NMR Spectroscopic Characterization of **Sn**_**3**_ and **Sn**_**5**_

The solution-state structures of the tri- and penta-tin complexes were examined by ^119^Sn NMR spectroscopy, which is highly sensitive to changes in tin oxidation state, coordination environment, and metal–metal bonding. The ^119^Sn NMR spectrum of the trinuclear complex **Sn**_**3**_, recorded in THF at room temperature, displays two resonances at δ = −451.6 ppm and −459.6 ppm (see Figure 4a). The presence of only two signals is consistent with a symmetric trinuclear structure, in which the two terminal L^Me^Sn units are chemically equivalent and distinct from the central L^BArF^Sn fragment. Both resonances are significantly shifted relative to the starting materials **Sn**_**2**_ (δ = −71.1 ppm) and **[L** ^**BArF**^ **Sn­(THF)]**_**2**_ (δ = −626.1 ppm), reflecting substantial electronic redistribution upon Sn–Sn–Sn chain formation, see Figure 2 for comparison. This behavior parallels that observed for previously reported mixed-valence tri-tin systems, such as (L^Ph^Sn)_3_Cl^23^ and L^Ph^Sn–^Ph^PDPSn–L^Ph^Sn,^24^ supporting assignment of **Sn**_**3**_ as a neutral mixed-valence tri-tin complex featuring a central Sn­(II) atom flanked by two Sn­(I) termini. In contrast, the ^119^Sn NMR spectrum of the penta-tin complex **Sn**_**5**_ exhibits two broad resonances at δ = −186.4 ppm and −208.6 ppm, with an integration ratio of approximately 1:4.4 (Figure 4b). Based on the proposed linear structure L^Me^Sn^1^–L^Me^Sn^2^–L^BArF^Sn^3^–L^Me^Sn^4^–L^Me^Sn^5^, three distinct tin environments would, in principle, be expected in a 2:2:1 ratio. The observation of only two resonances is therefore attributed to the very similar electronic environments of the terminal (Sn^1^/Sn^5^) and inner (Sn^2^/Sn^4^) Sn­(I) sites, whose signals likely coalesce into a single broadened resonance integrating to four tin atoms, while the remaining resonance is assigned to the central L^BArF^Sn^3^(II) unit. Variable-temperature ^119^Sn NMR experiments were performed to probe possible signal resolution; however, cooling to −10 °C produced no significant spectral changes, and further cooling to −40 °C resulted in signal loss due to limited solubility and partial precipitation of the penta-tin complex. Consequently, further differentiation of the Sn­(I) environments could not be achieved under accessible conditions.

**Figure 4:**
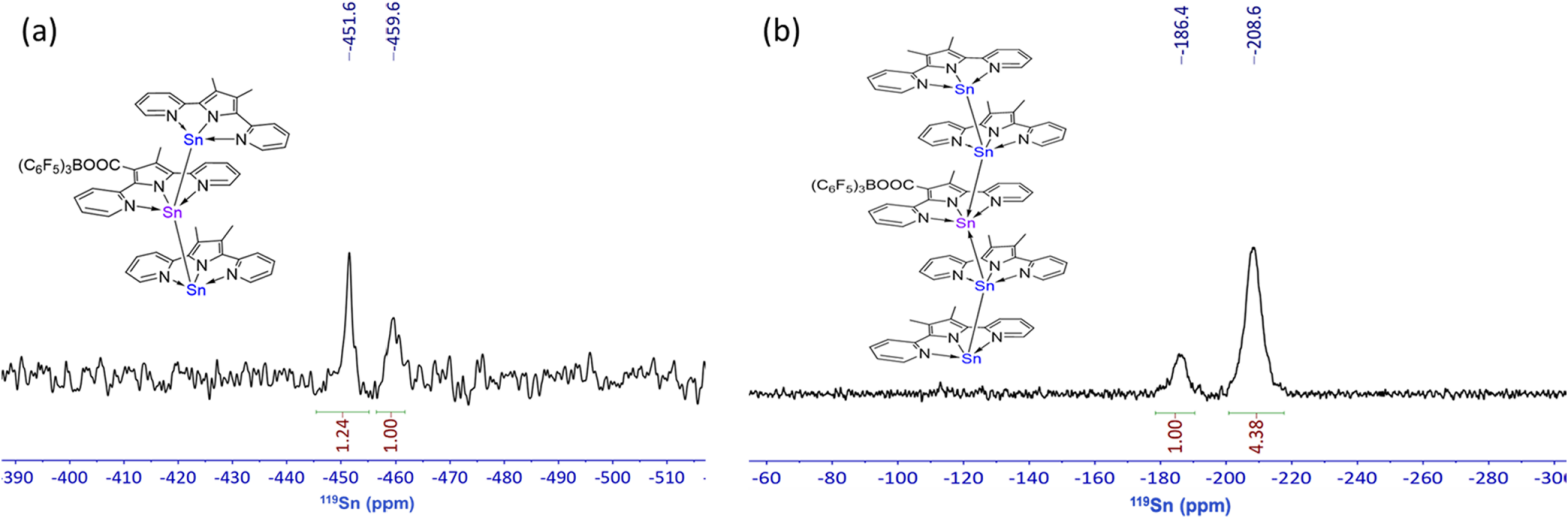
^119^Sn NMR spectra of (a) **Sn**_**3**_ and (b) **Sn**_**5**_.

Overall, the ^119^Sn NMR data clearly distinguish **Sn**_**2**_, **Sn**_**3**_, and **Sn**_**5**_ (also see Figure 2) at sufficiently high concentrations and provide strong evidence for the formation of discrete **Sn**_**3**_ and **Sn**_**5**_ mixed-valence chains. Importantly, the observed ^119^Sn chemical shifts and their overall trends across the di-, tri-, and penta-tin series are fully consistent with those observed in our previously reported tin complexes, in which lower-valent Sn­(I) sites resonate at more downfield chemical shifts relative to more electron-deficient Sn­(II) centers. Accordingly, the strongly upfield resonance of **[L** ^**BArF**^ **Sn­(THF)]**_**2**_ shifts downfield upon incorporation into **Sn**_**3**_ and **Sn**_**5**_ chains, consistent with increased electron donation from flanking Sn­(I) units, while the Sn­(I)-derived resonances shift upon chain extension, reflecting redistribution of electron density and enhanced metal–metal bonding along the extended tin backbone. These trends further support the proposed oxidation-state assignments and chain-growth model.

### UV–Vis Spectroscopy and Concentration-Dependent Solution Behavior

Given the intense coloration of the tin complexes, UV–vis spectroscopy was employed to probe their electronic structures and solution-state behavior. The absorption spectra of the **Sn**_**2**_, **Sn**_**3**_, and **Sn**_**5**_ were recorded in THF at room temperature using dilute solutions (ca. 10^–5^ M) to avoid saturation effects, see Figure 5.

**Figure 5:**
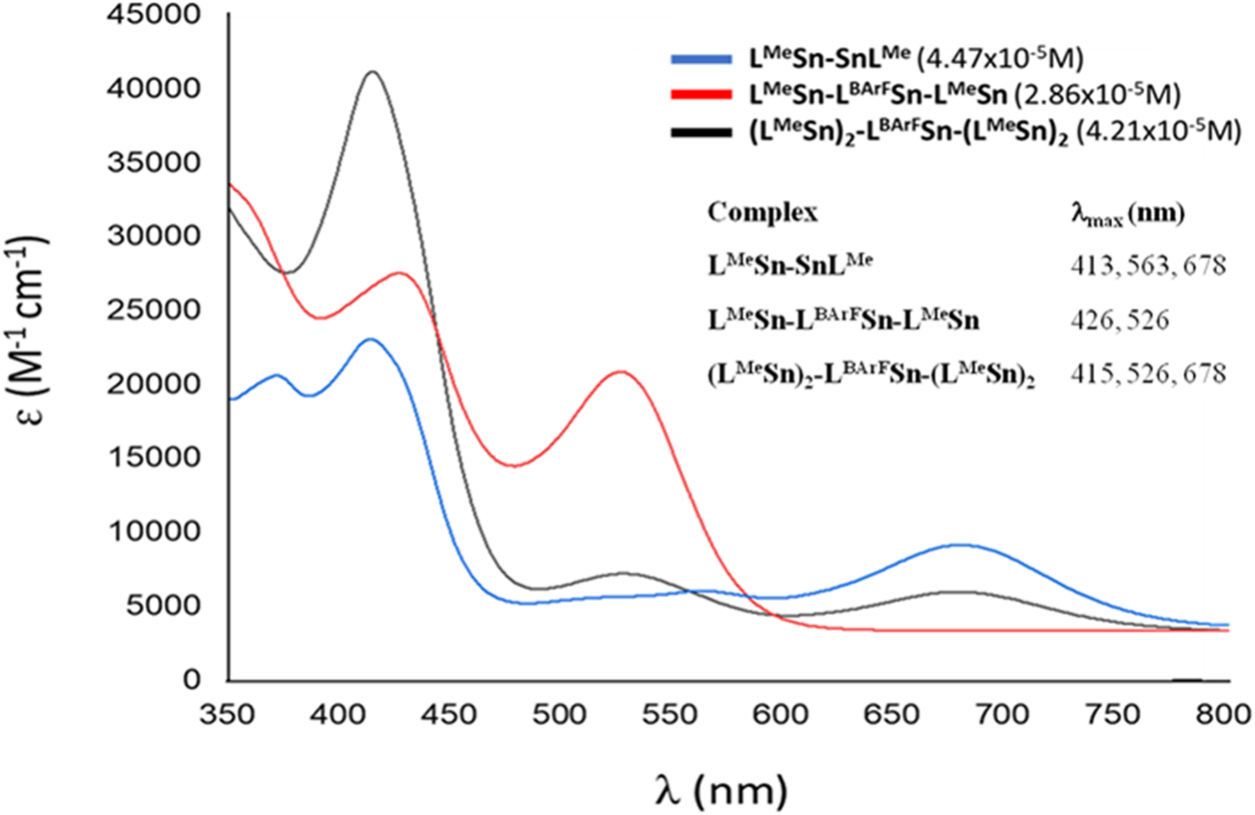
UV–vis spectra of L^Me^Sn-SnL^Me^ (**Sn**_**2**_, blue), L^Me^Sn-L^BArF^Sn-L^Me^Sn (**Sn**_**3**_, red) and (L^Me^Sn)_2_-L^BArF^Sn-(L^Me^Sn)_2_ (**Sn**_**5**_, black) in THF.

The distannyne **Sn**_**2**_ exhibits a strong absorption maximum at λ_max_ = 678 nm, consistent with its dark green color. This feature closely matches those observed for the related distannynes L^Ph^Sn–SnL^Ph^ (λ_max_ = 674 nm) and ^Me^LSn–Sn^Me^L (**Sn**_**2**_) (λ_max_ = 658 nm), which also display green coloration.^25^ These absorptions have previously been assigned, based on DFT calculations, to transitions from Sn–Sn σ-bonding orbitals to ligand-centered π* orbitals. The close correspondence among these systems indicates that peripheral ligand substitution has only a modest influence on the primary electronic transition associated with the Sn–Sn bond.

In contrast, the trinuclear complex **Sn**_**3**_ displays a markedly different absorption profile, with a dominant band at λ_max_ = 526 nm, consistent with its red color in solution. This feature closely resembles that of the previously reported cationic tri-tin complex (L^Ph^Sn)_3_PF_6_ (λ_max_ ≈ 515 nm). The pronounced blue shift relative to the distannynes reflects an increased HOMO–LUMO gap upon formation of the trinuclear Sn–Sn–Sn framework, arising from stabilization of the Sn–Sn σ-bonding orbitals through coordination to the central Sn­(II) unit. In contrast to (L^Ph^Sn)_3_Cl, which undergoes partial dissociation into L^Ph^Sn–SnL^Ph^ and L^Ph^SnCl in less polar solvents, including THF, **Sn**_**3**_ remains spectroscopically intact under identical conditions. This enhanced stability is consistent with its neutral charge and stronger Sn–Sn interactions enabled by the less sterically demanding L^Me^ ligand and the highly Lewis-acidic L^BArF^Sn fragment.

The UV–vis spectrum of the penta-tin complex **Sn**_**5**_, recorded under similarly dilute conditions, exhibits absorption features at 415, 526, and 678 nm. Notably, this spectrum closely resembles a superposition of those observed for **Sn**_**2**_ and **Sn**_**3**_. Given that the penta-tin complex appears dark brown in the solid state and in more concentrated solutions, whereas the trinuclear complex is red, the similarity of their UV–vis spectra suggest a concentration-dependent equilibrium in solution. Specifically, at very low concentrations (∼10^–5^ M, UV–vis conditions), the **Sn**_**5**_ complex undergoes partial dissociation into ditin **Sn**_**2**_ and tri-tin **Sn**_**3**_ fragments, giving rise to absorption features characteristic of both species.

This interpretation is further supported by the insensitivity of the UV–vis spectrum to solvent effects: almost identical spectra were obtained in THF and in 1,2-difluorobenzene, a more polar but weakly coordinating solvent. These results indicate that the partial dissociation observed under dilute conditions is governed primarily by concentration, rather than by solvent coordination. In contrast, at higher concentrations (∼10^–2^–10^–3^ M, NMR conditions), the **Sn**_**5**_ complex maintains its integrity, as evidenced by its distinct dark brown color and by the absence of resonances attributable to ditin or tri-tin species in the ^119^Sn NMR spectra. From a thermodynamic perspective, this behavior is consistent with a reversible association–dissociation equilibrium in solution, in which dilution favors dissociation due to the accompanying increase in the number of molecular species and the associated positive entropy change, while higher concentrations shift the equilibrium toward association, stabilizing the extended **Sn**_**5**_ chain.

Taken together, the UV–vis data reveal that while the penta-tin complex is structurally well-defined at higher concentrations, it exhibits concentration-dependent speciation in dilute solution. This behavior highlights the delicate balance between metal–metal bonding interactions and entropic effects in extended main-group metal chains and underscores the importance of complementary spectroscopic techniques in elucidating their solution-state structures.

### Raman Spectroscopy

Since all complexes synthesized in this study contain Sn–Sn bonds, Raman spectroscopy was employed to probe the presence of Sn–Sn stretching vibrations. The solid-state Raman spectra of L^Me^Sn–SnL^Me^ (**Sn**_**2**_), L^Me^Sn–L^BArF^Sn–L^Me^Sn (**Sn**_**3**_), and (L^Me^Sn)_2_–L^BArF^Sn–(L^Me^Sn)_2_ (**Sn**_**5**_) each exhibit a distinct vibrational band around 216 cm^–1^ (Figure 6). This feature closely matches the reported Sn–Sn stretching vibration at 208 cm^–1^ for Ph_3_Sn–SnEt_3_,^28^ confirming the presence of Sn–Sn σ-bonding in all three complexes. Notably, the Raman spectra of the **Sn**_**2**_, **Sn**_**3**_, and **Sn**_**5**_ species are highly similar, indicating that Raman spectroscopy is not sufficiently sensitive to differentiate chain length or nuclearity in these systems. Instead, the technique primarily reflects the presence of Sn–Sn bonding motifs, while distinctions among **Sn**_**2**_, **Sn**_**3**_, and **Sn**_**5**_ frameworks require complementary spectroscopic methods. These results are consistent with the localized nature of the Sn–Sn stretching mode and support the structural assignments derived from NMR, UV–vis, and X-ray absorption analyses.

**Figure 6:**
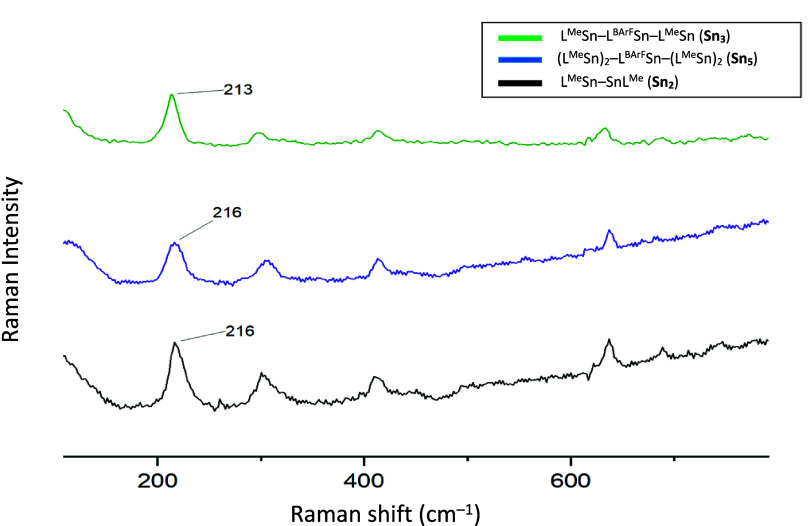
Raman spectra of L^Me^Sn–SnL^Me^ (**Sn**_**2**_), L^Me^Sn–L^BArF^Sn–L^Me^Sn (**Sn**_**3**_), and (L^Me^Sn)_2_–L^BArF^Sn–(L^Me^Sn)_2_ (**Sn**_**5**_).

### X-ray Absorption Spectroscopy (XAS)

To further elucidate the electronic structures and local coordination environments of the tin centers, X-ray absorption spectroscopy (XAS) measurements were performed at the Sn K-edge.^29–31^ Both X-ray absorption near-edge structure (XANES) and extended X-ray absorption fine structure (EXAFS) analyses were used to compare the tri- and penta-tin complexes with the Sn­(I) and Sn­(II) starting materials L^Me^Sn–SnL^Me^ (**Sn**_**2**_) and **[L** ^**BArF**^ **Sn­(THF)]**_**2**_, respectively. After normalization of the XANES spectra, differences in the white-line intensity provide insight into the relative oxidation states of the tin centers (Figure 7a).^31^ The divalent tin complex **L** ^**BArF**^ **Sn** exhibits the most intense white-line feature, while the univalent distannyne **Sn**_**2**_ shows the weakest. The trinuclear complex L^Me^Sn–L^BArF^Sn–L^Me^Sn (**Sn**_**3**_) displays a white-line intensity intermediate between these two extremes, consistent with an average tin oxidation state of +1.33, whereas the penta-tin complex (L^Me^Sn)_2_–L^BArF^Sn–(L^Me^Sn)_2_ (**Sn**_**5**_) exhibits a slightly lower average oxidation state of +1.2 (Figure 6d). These trends are in good agreement with the mixed-valence formulations proposed on the basis of NMR spectroscopy.

**Figure 7:**
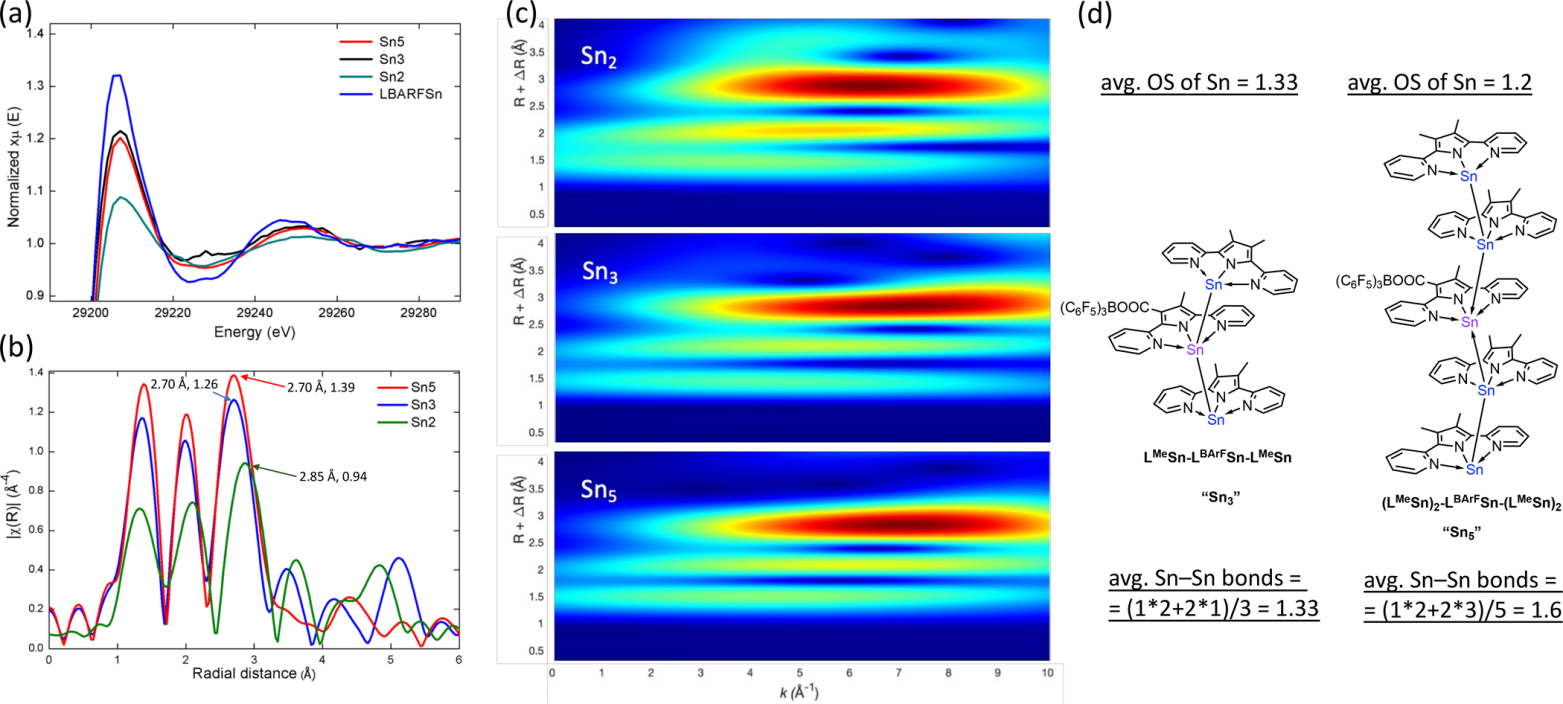
(a) Expanded Sn K-edge XANES spectra, (b) EXAFS spectra, and (c) wavelet transform analysis of the EXAFS data of the tri-tin L^Me^Sn–L^BArF^Sn–L^Me^Sn (**Sn**_**3**_), the penta-tin (L^Me^Sn)_2_–L^BArF^Sn–(L^Me^Sn)_2_ (**Sn**_**5**_) and selected reference compounds. (d) Schematic representations of **Sn**_**3**_ and **Sn**_**5**_ structures, together with their average oxidation states (avg. OS) and average numbers of Sn–Sn bonds per tin atom.

Fourier-transformed EXAFS spectra (Figure 7b) provide information on the local coordination environments of the tin atoms. For **Sn**_**2**_, three primary scattering features are observed between 1 and 3 Å, which can be assigned, based on the known crystal structure, to Sn–N­(pyrrole), Sn–N­(pyridine), and Sn–Sn interactions. **Sn**_**3**_ and **Sn**_**5**_ complexes exhibit similar features at comparable radial distances, indicating preservation of the local coordination geometry around tin upon chain extension. The Sn–Sn scattering contribution, manifested by the peak near ∼2.7 Å in the Fourier-transformed spectra (Figure 7b), increases in amplitude from **Sn**_**2**_ to **Sn**_**3**_ and **Sn**_**5**_, consistent with an increasing number of Sn–Sn interactions.

Further insight is provided by wavelet transform analysis of the EXAFS data (Figure 7c). This method correlates radial distance (*R*) with photoelectron wavevector (*k*), enabling discrimination of different scattering contributions based on the atomic mass of the scatterers: lighter atoms generate intensity at lower *k* values, whereas heavier atoms contribute at higher k values. In the wavelet contours of **Sn**_**2**_, **Sn**_**3**_ and **Sn**_**5**_, three prominent features are observed. The features centered at *R* ∼ 1.5 Å and ∼2.1 Å appear at relatively low k values and are characteristic of Sn–N scattering paths, while the feature at *R* ∼ 2.7 Å corresponds to scattering from heavy atoms, namely Sn–Sn interactions. Notably, the center of the Sn–Sn-related feature shifts progressively toward higher k values from **Sn**_**2**_ to **Sn**_**3**_ and **Sn**_**5**_, reflecting an increased contribution from Sn–Sn scattering as the number of Sn–Sn bonds along the chain increases.

Consistent with these observations, the |χ­(*R*)| intensity associated with Sn–Sn scattering is lowest for the distannyne **Sn**_**2**_, higher for the trinuclear **Sn**_**3**_, and highest for the penta-tin **Sn**_**5**_, although the difference between the latter two is modest.^29^ This limited increase is expected, as EXAFS predominantly probes local coordination environments and is inherently less sensitive to long-range chain length. These trends qualitatively mirror the increasing average Sn–Sn connectivity per tin atom upon chain extension: 1.0 in **Sn**_**2**_, 1.33 in **Sn**_**3**_, and 1.6 in **Sn**_**5**_ (Figure 7d). Collectively, the Fourier-transformed EXAFS, wavelet analysis, and |χ­(*R*)| trends corroborate the proposed **Sn**_**3**_ and **Sn**_**5**_ chain structures and confirm the presence of multiple Sn–Sn interactions within extended linear frameworks. Importantly, the XAS results are fully consistent with the mixed-valence formulations inferred from ^119^Sn NMR spectroscopy and with the concentration-dependent solution behavior revealed by UV–vis spectroscopy, providing a coherent and internally corroborated picture of the electronic structures and bonding motifs in these extended tin chains.

### DFT Calculations and Structural Analysis of **Sn_3_** and **Sn_5_**

To further substantiate the structural assignments of the tri- and penta-tin complexes in the absence of crystallographic data, density functional theory (DFT) calculations were performed to evaluate plausible isomers and their relative stabilities, as well as to provide insight into the bonding, charge distribution, and reaction mechanisms (see Computational Details in the Experimental Section). To validate this computational approach, the optimized structures of L^Me^Sn–SnL^Me^ (**Sn**_**2**_), (L^Ph^Sn)_3_Cl, and L^Ph^Sn–^Ph^PDPSn–SnL^Ph^ were compared with their experimentally determined X-ray crystal structures. The calculated bond metrics show excellent agreement with crystallographic data, particularly for Sn–Sn bond lengths (deviations <1%; see Tables S7–S9 in the Supporting Information), indicating that this level of theory provides a reliable description of dipyridylpyrrolate-supported tin clusters.

For the trinuclear and pentanuclear systems, multiple structural isomers were considered, differing in the position of the L^BArF^Sn­(II) motif along the metal chain. In addition to the experimentally proposed structures of **Sn**_**3**_ and **Sn**_**5**_ featuring a central L^BArF^Sn­(II) unit, alternative isomers (denoted **Sn**_**3**_ **_B1**, **Sn**_**5**_ **_B1**, and **Sn**_**5**_ **_B2**; see Figures S35–S37) were examined, in which the L^BArF^Sn­(II) fragment occupies terminal or off-center positions within the chain. In all cases, the isomers with the L^BArF^Sn­(II) unit located at the central position are calculated to be the most stable (Figure 8b). Specifically, **Sn**_**3**_ is more stable than **Sn**_**3**_ **_B1** by 7.1 kcal·mol^–1^, while **Sn**_**5**_ is more stable than **Sn**_**5**_ **_B1** and **Sn**_**5**_ **_B2** by 38.0 and 2.9 kcal·mol^–1^, respectively. These results are fully consistent with the symmetry observed in the ^119^Sn NMR spectra and the averaged structural features inferred from XAS, thereby providing strong computational support for the proposed linear, homocatenated tri- and penta-tin frameworks. Analysis of the optimized geometries further supports these assignments. In the trinuclear complex Sn_3_, the Sn–Sn distances (2.92–2.94 Å) are slightly shorter than those in the corresponding isomer **Sn**_**3**_ **_B1** (2.94–2.99 Å), indicating stronger metal–metal interactions in the centrally coordinated structure, with all values falling within the typical range for single Sn–Sn bonds (ca. 2.80–3.05 Å).^32^ In the pentanuclear system, the preferred structure **Sn**_**5**_ exhibits four Sn–Sn contacts in the range of 2.97–3.23 Å, whereas the higher-energy isomers **Sn**_**5**_ **_B1** and **Sn**_**5**_ **_B2** each contain an elongated Sn–Sn contact exceeding 3.40 Å, indicative of significantly weakened or nonbonding interactions. These unfavorable distortions likely contribute to the higher energies of these isomers and further rationalize the preference for the centrally positioned L^BArF^Sn­(II) motif. Importantly, the observation of only two ^119^Sn resonances with an approximate 1:4 ratio is inconsistent with asymmetric isomers and strongly supports a symmetric structure featuring a central Sn­(II) unit.

**Figure 8:**
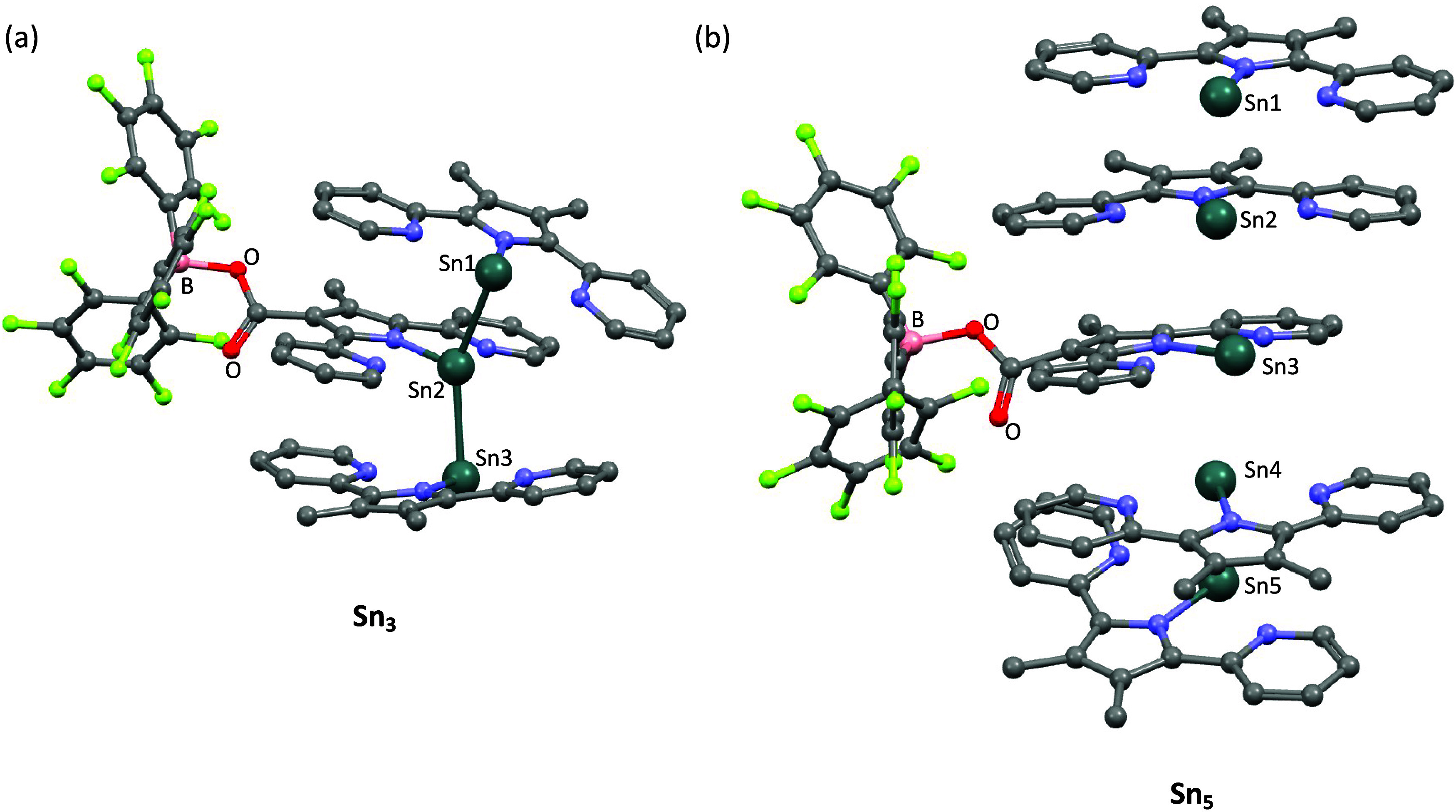
Optimized structures of (a) **Sn**_**3**_ and (b) **Sn**_**5**_. Selected interatomic distances (Å) and angles (°): (a) Sn(1)–Sn(2) 2.942, Sn(2)–Sn(3) 2.917, Sn(1)–Sn(2)–Sn(3) 150.70; (b) Sn(1)–Sn(2) 3.025, Sn(2)–Sn(3) 3.227, Sn(3)–Sn(4) 3.101, Sn(4)–Sn(5) 2.973, Sn(1)–Sn(2)–Sn(3) 159.45, Sn(2)–Sn(3)–Sn(4) 157.86, Sn(3)–Sn(4)–Sn(5) 156.98. H atoms have been omitted for clarity.

To gain insight into the formation mechanism, natural bond orbital (NBO) and frontier molecular orbital analyses were performed. The L^BArF^Sn­(II) fragment features a vacant *p* orbital oriented perpendicular to the ligand plane, which constitutes the LUMO (Figure 9a) and accounts for its pronounced Lewis acidity. Consistent with this, the Sn center carries a relatively high positive charge (NPA = +1.48). In contrast, the distannyne **Sn**_**2**_ possesses lone pairs at each Sn­(I) center (NPA = +0.65), rendering it a potential Lewis donor. The HOMO of **Sn**_**2**_ corresponds primarily to a σ-type Sn–Sn bonding orbital, while the HOMO – 3 and HOMO – 4 are localized lone pairs on the tin centers, further supporting its nucleophilic character (Figure 9b,c).

**Figure 9:**
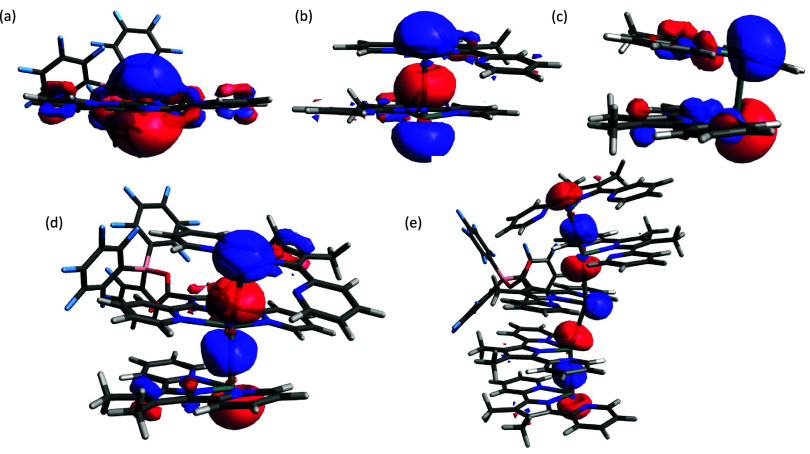
(a) LUMO of L^BArF^Sn­(II); (b) HOMO and (c) HOMO – 3 of **Sn**_**2**_; HOMOs of (d) **Sn**_**3**_, and (e) **Sn**_**5**_.

Based on our previous studies on triple-decker tri-tin complexes, such as (L^Ph^Sn)_3_Cl and L^Ph^Sn–^Ph^PDPSn–SnL^Ph^, which arise from reactions of distannynes with Lewis-acidic Sn­(II) precursors, a similar pathway is proposed here (Scheme 4). The formation of the trinuclear complex **Sn**_**3**_ can be rationalized by an initial donor–acceptor interaction between Sn_2_ and L^BArF^Sn­(II), likely leading to an intermediate isomer (**Sn**_**3**_ **_B1**), followed by structural reorganization, potentially via dissociation–association processes, to yield the thermodynamically favored linear structure. While a two-electron donor–acceptor pathway is plausible, the relatively weak Sn–Sn bond in **Sn**_**2**_ suggests that pathways involving partial homolytic dissociation cannot be excluded. Indeed, studies by Power and co-workers have demonstrated reversible Sn–Sn bond cleavage in distannynes to generate Sn-centered radicals, which may similarly participate in the formation of **Sn**_**3**_.^33^

**Scheme 4:**
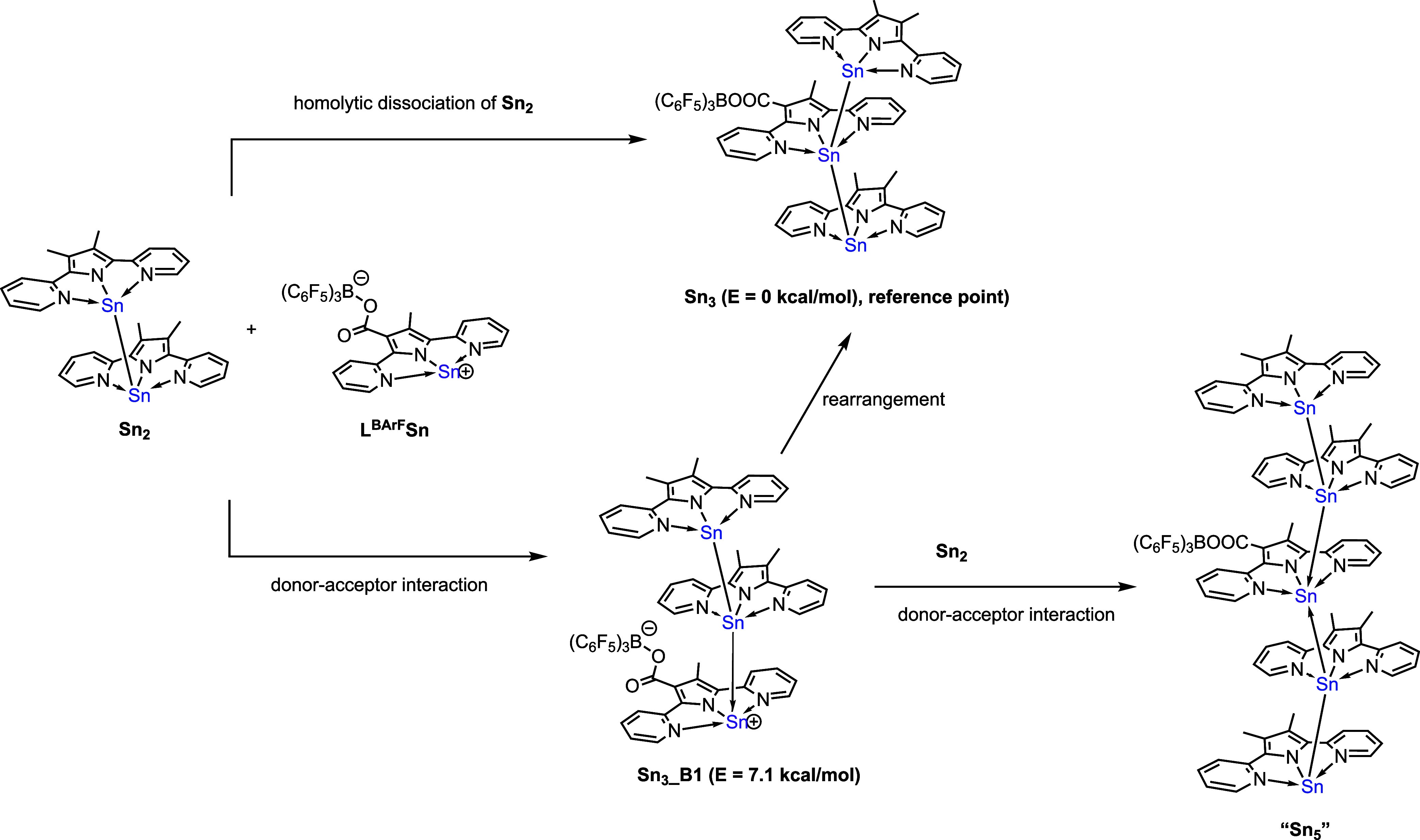
Proposed Pathways for the Formation of **Sn**_**3**_ and **Sn**_**5**_

Additional insight is provided by natural population analysis (NPA). Upon formation of **Sn**_**3**_, the NPA charge of the L^Me^Sn fragments increases from +0.65 in Sn_2_ to +0.89, whereas the charge at the L^BArF^Sn­(II) center decreases markedly from +1.48 to +0.83. These changes indicate net electron donation from the Sn_2_ fragment to the L^BArF^Sn­(II) unit, consistent with oxidation at the Sn­(I) centers and concomitant reduction at the Sn­(II) center. This charge redistribution parallels the trends observed in the ^119^Sn NMR spectra, where the L^Me^Sn-derived resonances shift upfield, while the L^BArF^Sn­(II) resonance shifts downfield upon incorporation into **Sn**_**3**_.

Orbital analysis further reveals that the HOMOs of both **Sn**_**3**_ and **Sn**_**5**_ are delocalized σ-type orbitals extending along the Sn–Sn backbone, indicating electronic communication across the metal chain. In addition to direct Sn–Sn bonding interactions, the optimized structures of **Sn**_**3**_ and **Sn**_**5**_ exhibit multiple π–π interactions between the pyridyl and pyrrolyl moieties, with centroid–centroid distances in the range of 3.47–3.93 Å (Figures S33 and S34). Despite the steric demand of the COOB­(C_6_F_5_)_3_ substituent on the L^BArF^Sn­(II) fragment, the pincer ligand frameworks remain arranged in a nearly parallel fashion, facilitating these stabilizing interactions. These distances fall within the typical range for π–π stacking interactions, indicating that interligand interactions contribute to stabilization of the extended framework.

Extending this analysis to the pentanuclear system, a plausible chain-extension pathway may involve reaction of a trinuclear intermediate, such as **Sn**_**3**_ **_B1**, with an additional equivalent of **Sn**_**2**_ to form **Sn**_**5**_ (Scheme 4). Although **Sn**_**3**_ is calculated to be more stable than **Sn**_**3**_ **_B1**, the energy difference is modest (7.1 kcal mol^–1^), suggesting that access to **Sn**_**3**_ **_B1** under the reaction conditions may be feasible. Notably, the Sn center of the L^BArF^Sn fragment in **Sn**_**3**_ **_B1** remains relatively electron-deficient (NPA charge = +0.87), which could allow further interaction with an additional **Sn**_**2**_ unit and promote chain extension via donor–acceptor interaction. However, the present data do not allow us to distinguish definitively whether the terminal **Sn**_**2**_ fragments in **Sn**_**5**_ are incorporated as intact **Sn**_**2**_ units or formed through partial dissociation of **Sn**_**2**_ followed by reassembly. Therefore, the proposed pathway should be regarded as a plausible mechanistic scenario rather than a fully established mechanism. Consistent with this picture, NPA analysis shows that the Sn center of the L^BArF^Sn fragment is slightly further reduced in **Sn**_**5**_ (charge = +0.82) relative to **Sn**_**3**_, while the four L^Me^Sn fragments exhibit NPA charges in the range of +0.76–0.79, intermediate between those observed in **Sn**_**2**_ and **Sn**_**3**_. This distribution reflects partial delocalization of electron density along the extended Sn–Sn chain. Overall, the evolution of NPA charges across **Sn**_**2**_, **Sn**_**3**_, and **Sn**_**5**_ is consistent with progressive charge redistribution accompanying stepwise chain growth and aligns well with the corresponding trends observed in the ^119^Sn NMR spectra. Taken together, these results suggest that electronic factors, together with secondary interligand interactions, contribute to the formation and stabilization of the extended Sn–Sn chain.

## Conclusions

We report ligand-unsupported, linear mixed-valence tri-tin and penta-tin complexes assembled through controlled metal–metal donor–acceptor interactions. By combining a nucleophilic Sn­(I) distannyne **Sn**_**2**_ supported by a sterically undemanding dipyridylpyrrolate ligand with a neutral yet highly Lewis-acidic Sn­(II) synthon, stepwise chain growth from **Sn**_**2**_ to **Sn**_**3**_ and ultimately to **Sn**_**5**_ featuring four consecutive Sn–Sn bonds was achieved. To our knowledge, this compound represents the longest homocatenated molecular tin chain reported to date. Multinuclear NMR, UV–vis, Raman, and X-ray absorption spectroscopies, together with elemental analysis, provide a consistent picture of the **Sn**_**3**_ and **Sn**_**5**_ structures and reveal a concentration-dependent association–dissociation equilibrium in solution. Complementary DFT calculations further support these structural assignments by identifying the centrally located L^BArF^Sn­(II) motif as the thermodynamically preferred arrangement and by rationalizing the observed spectroscopic trends. This work demonstrates that extended main-group metal chains can be constructed without preorganized multidentate ligands, offering a new strategy for EMAC synthesis based on intrinsic metal–metal interactions.

## Experimental Section

### General Information

All manipulations with moisture and oxygen-sensitive materials were performed in a nitrogen-filled glovebox. Solvents were dried and deaerated using a solvent system (AsiaWong Enterprise co., Ltd.) before use. Benzene-d_6_ and tetrahydrofuran-d_8_ (THF-*d*_8_) were dried over sodium and benzophenone, degassed by three freeze–pump–thaw cycles, and stored under nitrogen over 3 Å molecular sieves. The compounds L^Me^H,^26^ L^MeCOOMe^H,^26^ SnN­(SiMe_3_)_2_Cl,^34^ and Sn­[N­(SiMe_3_)_2_]_2_ ^35^ were synthesized according to literature procedures. The NMR spectra were recorded using Varian 400, and Varian 600 MHz spectrometers. The NMR spectra were referenced to residual protonated solvent for ^1^H NMR (3.58 ppm for compound in THF-*d*_8_; 7.16 ppm for compound in benzene-*d*_6_), to deuterated solvent for ^13^C NMR (67.21 ppm for compound in THF-*d*_8_ and 128.06 for compound in benzene-*d*_6_), ^119^Sn NMR spectra were referenced to tetrabutyl tin at–11.7 ppm. All NMR measurements were conducted at 25 °C unless otherwise noted. Complex multiplets are noted as “m” and broad resonances as “br”. Elemental analyses were performed using an Elementar vario EL CUBE (CHN-OS Rapid, German). UV–visible spectra were recorded using a Hitachi U-3010 spectrophotometer. 532 nm-excited Raman spectra were acquired using a laboratory-built spectrometer (see Supporting Information for details).

The XAS measurements were conducted using a linear accelerator with an electron energy of 50 MeV and a booster ring operating at 1.5 GeV. The storage ring also operated at 1.5 GeV with a Triple Band Achromat (TBA) magnetic lattice configuration. X-ray absorption spectroscopy (XAS) measurements, including X-ray absorption near edge spectra (XANES) and extended X-ray absorption fine structure (EXAFS) at the Sn K-edge, were performed using a Lytle detector at the 01C1 beamline of the Taiwan Light Source (TLS), National Synchrotron Radiation Research Center (NSRRC). The samples were sealed in airtight tubes under an argon atmosphere during data collection. The pre-edge baseline was subtracted, and the spectra were normalized to the postedge region. EXAFS analysis was conducted by Fourier transforming the k^3^-weighted EXAFS oscillations to evaluate the contribution of different coordination shells to the Fourier transform peaks.

### Safety Notification

Great attention must be exercised when working with pyrophoric chemicals like *n*-BuLi and K­[BH^S^Bu_3_]. The experiments were carried out using these substances under inert conditions, employing the Schlenk technique or gloveboxes.

### Synthesis of All Compounds

#### Synthesis of **L** ^**MeCOOH**^ **H**

A solution of L^MeCOOMe^H (1.758 g, 6.0 mmol) in THF (50 mL) was combined with an aqueous solution of NaOH (1.2 g, 30 mmol, 5 equiv) in water (200 mL). The resulting mixture was heated at 85 °C for 3 days. After cooling to room temperature, the reaction mixture was neutralized by the addition of saturated aqueous NH_4_Cl. The product was extracted with dichloromethane, and the organic layer was separated, dried over anhydrous MgSO_4_, and filtered. Removal of the solvent under reduced pressure afforded a yellow solid (1.078 g, 64% yield).

^**1**^ **H NMR** (400 MHz, CDCl_3_, 25 °C): δ 10.55 (s, 1H), 8.53 (dd, *J* = 12.6, 4.7 Hz, 2H), 7.91 (t, *J* = 7.9 Hz, 1H), 7.81–7.71 (m, 3H), 7.37–7.28 (m, 1H), 7.19 (t, *J* = 5.8 Hz, 1H), 2.77 (s, 3H) ppm. ^**13**^ **C­{** ^**1**^ **H} NMR** (101 MHz, CDCl_3_, 25 °C): δ 166.1, 149.3, 148.6, 146.1, 139.5, 137.2, 130.7, 129.0, 126.0, 122.6, 122.0, 121.2, 119.9, 12.9 ppm. Anal. Calcd for **L** ^**MeCOOH**^ **H** (C_16_H_13_N_3_O_2_): C, 68.81; H, 4.69; N, 15.05. Found: C, 69.29; H, 4.46; N, 15.23.

#### Synthesis of **L^BArF^H_2_**

A solution of B­(C_6_F_5_)_3_ (0.236 g, 0.50 mmol) in toluene (3 mL) was added dropwise to a toluene suspension of L^MeCOOH^H (0.139 g, 0.50 mmol). Owing to the poor solubility of L^MeCOOH^H in toluene, the reaction mixture was initially heterogeneous. Upon addition of B­(C_6_F_5_)_3_, the mixture gradually became a clear fluorescent yellow solution, followed by the rapid formation of a large amount of fluorescent yellow precipitate. After stirring at room temperature for 3 h, the solid was isolated by recrystallization from *n*-pentane to afford a fluorescent yellow solid (0.388 g, 98% yield).

^**1**^ **H NMR** (400 MHz, THF-*d*_8_, 25 °C): δ 11.75 (s, 1H), 8.78 (d, *J* = 5.6 Hz, 1H), 8.66 (d, *J* = 4.3 Hz, 1H), 8.55 (d, *J* = 8.4 Hz, 1H), 8.43 (t, *J* = 8.0 Hz, 1H), 7.87 (t, *J* = 7.7 Hz, 1H), 7.79 (d, *J* = 7.9 Hz, 1H), 7.72 (t, *J* = 6.6 Hz, 1H), 7.37–7.26 (m, 1H), 2.61 (s, 3H) ppm. ^**13**^ **C­{** ^**1**^ **H} NMR** (101 MHz, THF-*d*_8_, 25 °C): δ 171.7, 150.5, 150.2, 147.8, 145.6, 144.8, 141.5, 141.1, 138.7, 137.7, 136.3, 136.1, 126.7, 126.2, 124.3, 123.6, 123.1, 12.5 ppm. ^**19**^ **F­{** ^**1**^ **H} NMR** (376 MHz, THF-*d*_8_, 25 °C): δ−132.6,–161.3,–165.9 ppm. Analytical calcd for (L^BArF^H_2_)_2_·pentane, (C_73_H_38_B_2_F_30_N_6_O_4_): Calcd: C, 52.99; H, 2.31; N, 5.08. Found: C, 53.25; H, 2.51; N, 5.28.

#### Synthesis of L^Me^SnCl

A solution of Sn­[N­(SiMe_3_)_2_]­Cl (0.315 g, 1.0 mmol) in diethyl ether (3 mL) was added dropwise to a diethyl ether solution of L^Me^H (0.249 g, 1.0 mmol) (5 mL). Upon addition, the reaction mixture gradually turned yellow, accompanied by the formation of a yellow precipitate. After stirring at room temperature for 3 h, the solid was collected by filtration, washed several times with *n*-pentane, and dried under vacuum to afford L^Me^SnCl as a yellow solid (0.379 g, 94% yield). Yellow single crystals suitable for X-ray diffraction were obtained by diffusion of *n*-pentane into a THF solution of the product.

^**1**^ **H NMR** (400 MHz, THF-*d*_8_, 25 °C): δ 8.49 (d, *J* = 4 Hz, 2H), 7.82 (t, *J* = 7.7 Hz, 2H), 7.76 (d, *J* = 8.0 Hz, 2H), 7.14 (t, *J* = 6.2 Hz 2H), 2.35 (s, 6H) ppm. ^**13**^ **C­{** ^**1**^ **H} NMR** (101 MHz, THF-*d*_8_, 25 °C): δ 147.3, 139.7, 120.5, 120.0, 11.4 ppm. ^**119**^ **Sn­{** ^**1**^ **H} NMR** (224 MHz, THF-*d*_8_, 25 °C): δ−356.3 ppm. Anal. Calcd for L^Me^SnCl (C_16_H_14_ClN_3_Sn): C, 47.75; H, 3.51; N, 10.44. Found: C, 47.87; H, 3.31; N, 10.46.

#### Synthesis of L^Me^Sn–SnL^Me^ (Sn_2_)

A solution of L^Me^SnCl (0.202 g, 0.50 mmol) in THF (5 mL) was treated with a diluted THF solution of K­[BH^S^Bu_3_] (0.50 mL of a 1 M solution, 0.50 mmol, diluted with 1 mL of THF), which was added dropwise. During the addition, the color of the reaction mixture gradually changed from light green to dark green. After stirring at room temperature for 1 h, the solvent was removed under reduced pressure. The residue was extracted with toluene and filtered, and the filtrate was concentrated to the minimum volume required for complete dissolution. Dark green crystals of L^Me^Sn–SnL^Me^ were obtained by layering the solution with *n*-pentane at −35 °C (0.151 g, 82% yield).

^**1**^ **H NMR** (400 MHz, C_6_D_6_, 25 °C): δ 7.75 (d, *J* = 4.7 Hz, 4H), 7.29 (d, *J* = 8.3 Hz, 4H), 6.95 (t, *J* = 7.7 Hz, 4H), 6.25–6.12 (m, 4H), 2.25 (s, 12H) ppm. ^**13**^ **C­{** ^**1**^ **H} NMR** (101 MHz, C_6_D_6_, 25 °C): δ 150.8, 145.2, 134.8, 129.3, 125.7, 121.4, 118.9, 116.0, 12.1 ppm. ^**119**^ **Sn­{** ^**1**^ **H} NMR** (224 MHz, THF-*d*_8_, 25 °C): δ −71.1 ppm. Anal. Calcd for L^Me^Sn–SnL^Me^ (C_32_H_28_N_6_Sn_2_): C, 52.36; H, 3.85; N, 11.45. Found: C, 52.63; H, 3.91; N, 11.02.

#### Synthesis of **[L** ^**BArF**^ **Sn­(THF)]**_**2**_

A solution of Sn­[N­(SiMe_3_)_2_]_2_ (0.141 g, 0.32 mmol) in THF (3 mL) was added dropwise to a THF solution of L^BArF^H_2_ (0.255 g, 0.32 mmol) (5 mL). During the addition, a yellow solid gradually precipitated. After stirring at room temperature for 3 h, the reaction mixture was concentrated to the minimum volume of THF required for complete dissolution. Fluorescent yellow crystals of **[L** ^**BArF**^ **Sn­(THF)]**_**2**_ were obtained by layering the solution with *n*-pentane (0.202 g, 64% yield).

^**1**^ **H NMR** (400 MHz, THF-*d*_8_, 25 °C): δ 8.47 (d, *J* = 4.6 Hz, 2H), 8.42 (d, *J* = 4.7 Hz, 2H)., 8.00 (t, *J* = 8.5 Hz, 2H), 7.53 (d, *J* = 6.0 Hz, 2H), 7.45–7.35 (m, 2H), 7.31–7.23 (m, 2H), 6.76 (br, 2H), 6.42 (br, 2H), 2.25 (s, 6H) ppm. ^**13**^ **C­{** ^**1**^ **H} NMR** (101 MHz, THF-*d*_8_, 25 °C): δ 151.2, 150.1, 149.0, 147.6, 146.7, 141.3, 138.9, 138.2, 136.4, 134.6, 125.7, 123.6, 123.3, 122.5, 120.7, 120.3, 12.4 ppm. ^**19**^ **F­{** ^**1**^ **H} NMR** (376 MHz, THF-*d*_8_, 25 °C): δ −131.9, −160.9, −165.2 ppm. ^**119**^ **Sn­{** ^**1**^ **H} NMR** (224 MHz, THF-*d*_8_, 50̊C): δ−626.09 ppm. Anal. Calcd for **[L** ^**BArF**^ **Sn­(THF)]**_**2**_ (C_76_H_38_B_2_F_30_N_6_O_6_Sn_2_): C, 46.57; H, 1.95; N, 4.29. Found: C, 46.09; H, 1.72; N, 4.71.

#### Synthesis of L^Me^Sn–L^BArF^Sn–L^Me^Sn (Sn_3_)

A solution of **[L** ^**BArF**^ **Sn­(THF)]**_**2**_ (0.020 g, 0.010 mmol) in THF (3 mL) was added dropwise to a THF solution of L^Me^Sn–SnL^Me^ (0.0147 g, 0.020 mmol) (5 mL). Upon addition, the solution color changed immediately from dark green to deep red. After stirring at room temperature for 1 h, the solvent was reduced under vacuum to the minimum volume required to maintain complete dissolution. Addition of *n*-pentane induced recrystallization, affording the trinuclear tin complex as a black solid (0.029 g, 89% yield).

^**1**^ **H NMR** (400 MHz, THF-*d*_8_, 25 °C): δ 8.58 (br, 1H), 7.45 (m, 5H), 7.32 (m, 5H), 7.20 (m, 3H), 7.10 (m, 5H), 6.67 (m, 1H), 6.60 (m, 4H), 2.17 (s, 3H), 1.84 (s, 12H) ppm. ^**13**^ **C­{** ^**1**^ **H} NMR** (101 MHz, THF-*d*_8_, 25 °C): δ 151.3, 151.3, 150.7, 150.5, 149.9, 148.4, 145.8, 145.7, 144.4, 140.2, 138.7, 138.6, 138.5, 138.4, 138.2, 136.4, 133.7, 133.6, 132.2, 128.3, 124.8, 124.7, 123.7, 123.7, 120.7, 120.2, 119.9, 119.8, 119.5, 13.3, 11.2 ppm. ^**19**^ **F­{** ^**1**^ **H} NMR** (376 MHz, THF-*d*_8_, 25 °C): δ −131.8, −162.8, −166.6 ppm. ^**119**^ **Sn­{** ^**1**^ **H} NMR** (224 MHz, THF-*d*_8_, 25 °C): δ−451.6,–459.6 ppm. Anal. Calcd for **L** ^**Me**^ **Sn–L** ^**BArF**^ **Sn–L** ^**Me**^ **Sn** (C_66_H_39_BF_15_N_9_O_2_Sn_3_): C, 48.28; H, 2.39; N, 7.68. Found: C, 48.46; H, 2.53; N, 7.43

#### Synthesis of (L^Me^Sn)_2_–L^BArF^Sn–(L^Me^Sn)_2_ (Sn_5_)

A solution of **[L** ^**BArF**^ **Sn­(THF)]**_**2**_ (0.020 g, 0.010 mmol) in THF (3 mL) was added dropwise to a THF solution of L^Me^Sn–SnL^Me^ (0.029 g, 0.040 mmol) (5 mL). Upon addition, the solution color changed immediately from dark green to dark brown. The reaction mixture was stirred at room temperature for 1 h, after which THF was removed under reduced pressure to the minimum volume required to maintain complete dissolution. Diffusion of *n*-pentane into the concentrated solution induced recrystallization, affording the penta-tin complex as a black solid (0.041 g, 85% yield).

^**1**^ **H NMR** (400 MHz, THF-*d*_8_, 25 °C): δ 8.70 (d, J = 8.2 Hz, 1H), 7.35 (s, 2H), 7.29 (dd, J = 15.0, 7.4 Hz, 8H), 7.24 (br, 8H), 7.18 (d, J = 8.2 Hz, 1H), 7.02 (d, J = 8.0 Hz, 8H), 6.43 (d, J = 21.7 Hz, 10H), 2.20 (s, 3H), 1.83 (s, 24H) ppm. ^**13**^ **C­{** ^**1**^ **H} NMR** (101 MHz, THF-*d*_8_, 25 °C): δ 167.2, 151.1, 150.8, 150.5, 150.0, 148.4, 145.5, 144.9, 144.1, 140.2, 138.6, 138.2, 137.6, 137.3, 136.5, 136.3, 133.1, 131.9, 129.7, 129.0, 128.9, 128.1, 124.7, 124.2, 122.8, 120.0, 119.5, 118.7, 118.3, 13.6, 11.4 ppm. ^**19**^ **F­{** ^**1**^ **H} NMR** (376 MHz, THF-*d*_8_, 25 °C): δ−131.59,–162.97,–166.67 ppm. ^**119**^ **Sn­{** ^**1**^ **H} NMR** (224 MHz, THF-*d*_8_, 25 °C): δ−186.40,–208.56 ppm. Anal. Calcd for **(L** ^**Me**^ **Sn)**_**2**_ **–L** ^**BArF**^ **Sn–(L** ^**Me**^ **Sn)**_**2**_ (C_98_H_67_BF_15_N_15_O_2_Sn_5_): C, 49.54; H, 2.84; N, 8.84. Found: C, 49.33; H, 2.86; N, 8.41.

### Computational Details

Density functional theory (DFT) calculations were performed using the Gaussian 16 suite of programs.^36^ Geometry optimizations were carried out with the PBE0 hybrid functional,^37^ including Grimme’s D3 dispersion correction with Becke–Johnson damping.^38,39^ A mixed basis set was employed, using 6–31G­(d,p)^40,41^ for light atoms and the def2-TZVPP basis set for Sn.^42^ All geometries were fully optimized without symmetry constraints. Natural bond orbital (NBO) analyses were performed using the NBO 7.0 program.^43^

## Supplementary Material













## Notes

General considerations, synthetic procedures, compound characterizations, theoretical calculation details, and crystallographic data for all compounds (PDF)
**Sn_3__B1** calculated (XYZ)
**Sn_3_** calculated (XYZ)
**Sn_5__B1** calculated (XYZ)
**Sn_5__B2** calculated (XYZ)
**Sn_5_** calculated (XYZ)
