# Supplementary material for: Ligand-Unsupported Assembly of a Linear Penta-Tin Chain via Metal–Metal Donor–Acceptor Interactions
Source: Inorg Chem. 2026 Jun 23;65(26):14514–27. doi: 10.1021/acs.inorgchem.6c00602 (PMC13343519; doi:10.1021/acs.inorgchem.6c00602)
Supplement: Supplementary file 1 [file ic6c00602_si_001.pdf]

Supporting Information

Ligand-Unsupported Assembly of a Linear Penta-Tin Chain via Metal–Metal Donor–Acceptor Interactions

Yi-Chen Lin,[a] Kuheli Das,[a] Ankit Raj,[a] Li-Ching Shen,[a] You-Song Cheng,[a] Ting-Shen Kuo,[b] Han-Jung Li,[c]* Hirotsugu Hiramatsu,[a]* Sung-Fu Hung,[a],[d]* and Hsueh-Ju Liu,[a],[d]*

[a] Department of Applied Chemistry, National Yang Ming Chiao Tung University, Hsinchu City 300093, Taiwan

[b] Department of Chemistry, National Taiwan Normal University, Taipei 11677, Taiwan

[c] Department of Chemistry, Chung Yuan Christian University, Taoyuan City 320314, Taiwan

[d] Center for Emergent Functional Matter Science, National Yang Ming Chiao Tung University, Hsinchu City 300093, Taiwan

Corresponding author's E-mail: hsuehjuliu@nycu.edu.tw; sungfuhung@nycu.edu.tw; hiramatu@nycu.edu.tw; hjli@cycu.edu.tw

Table of content:

1. NMR SPECTRA OF ALL COMPOUNDS 2
2. DETAILS ON RAMAN MEASUREMENT AND DATA ANALYSIS....14
3. SINGLE-CRYSTAL X-RAY DIFFRACTION CRYSTAL STRUCTURE FIGURES AND DATA TABLES 15
4. X-RAY ABSORPTION SPECTROSCOPY 28
5. DFT CALCULATIONS 30

1. NMR spectra of all compounds

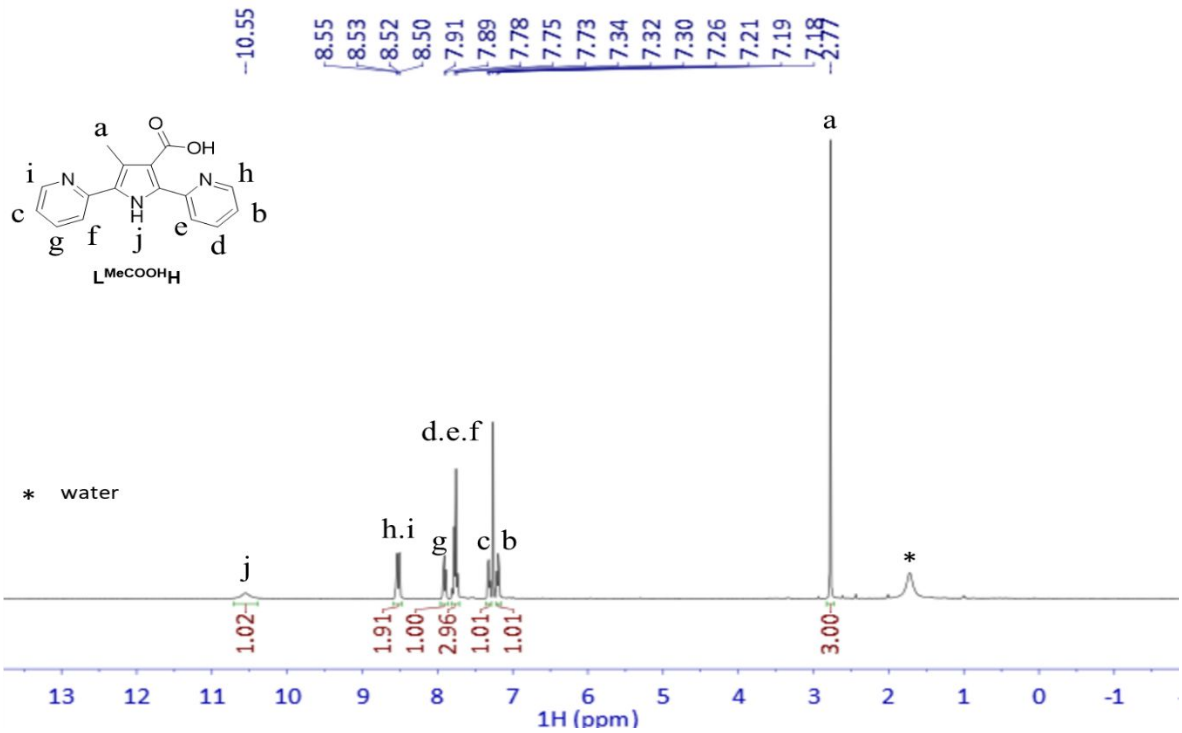

Figure S1. ^{1}H NMR spectrum of **L^{MeCOOH}H** in $CDCl_3$.

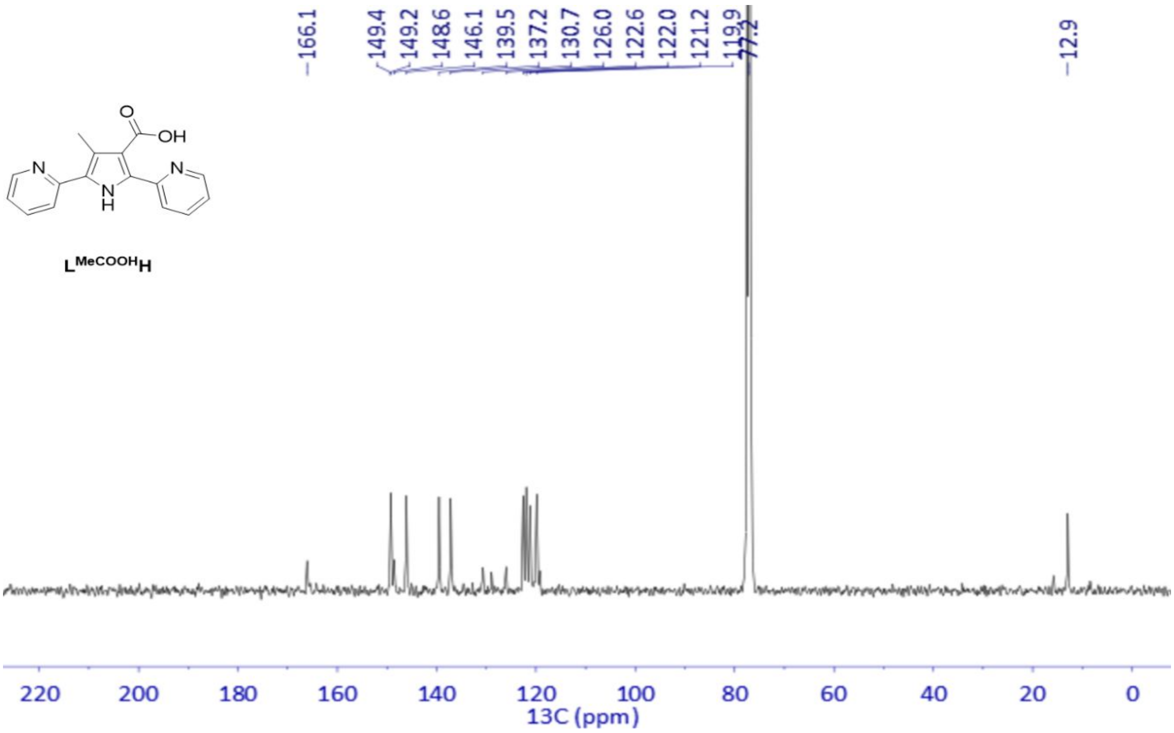

Figure S2. ^{13}C{^{1}H} NMR spectrum of **L^{MeCOOH}H** in $CDCl_3$

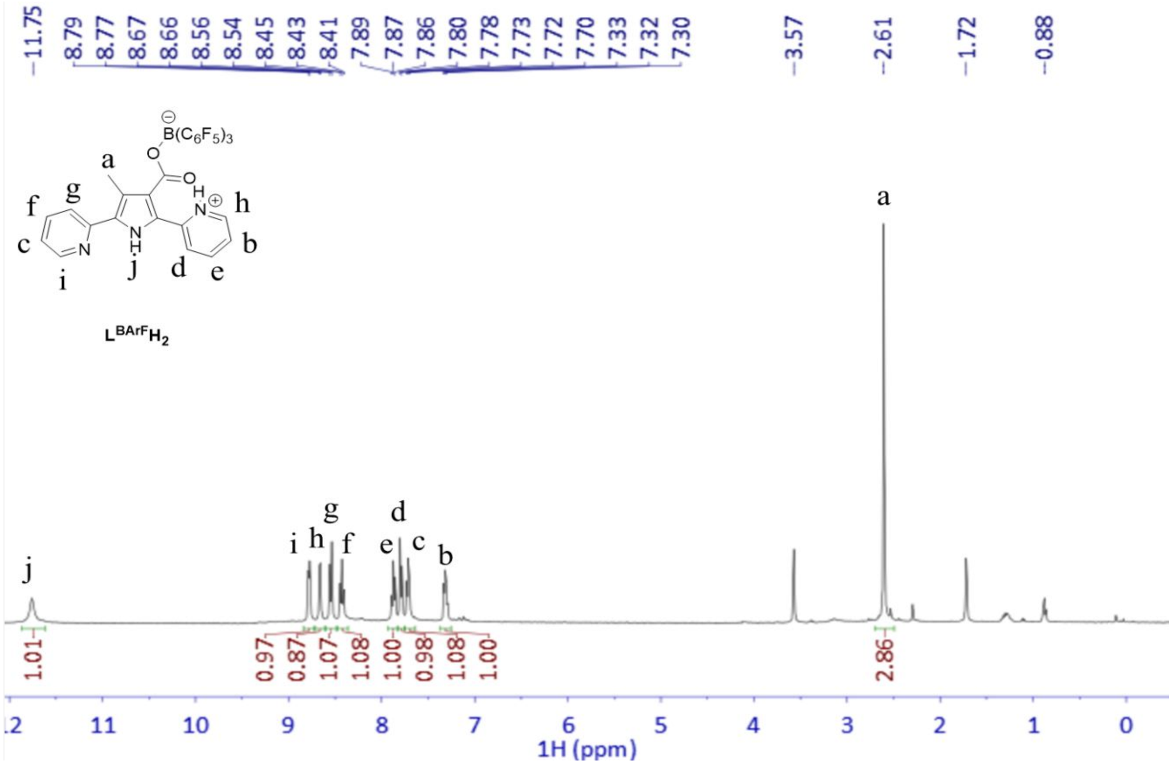

Figure S3. ^{1}H NMR spectrum of $\mathbf{L^{BARF}H_2}$ in THF-d_8

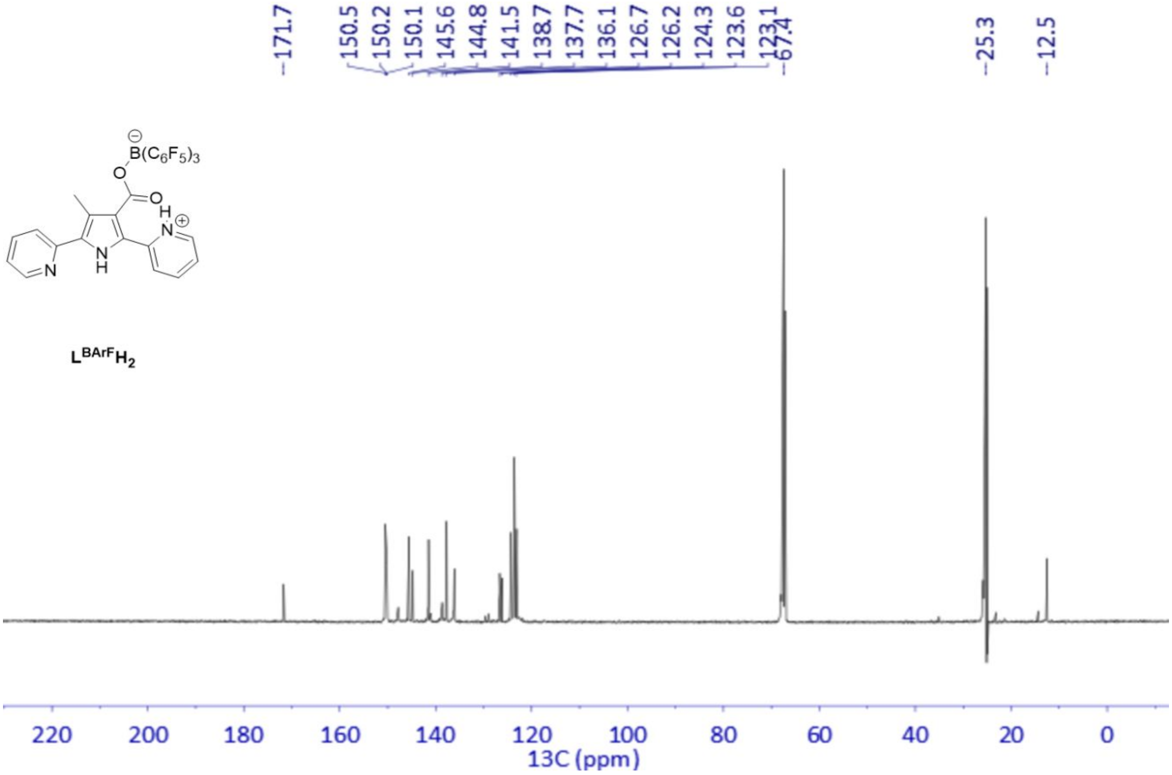

Figure S4. ^{1}H NMR spectrum of $\mathbf{L^{BARF}H_2}$ in THF-d_8

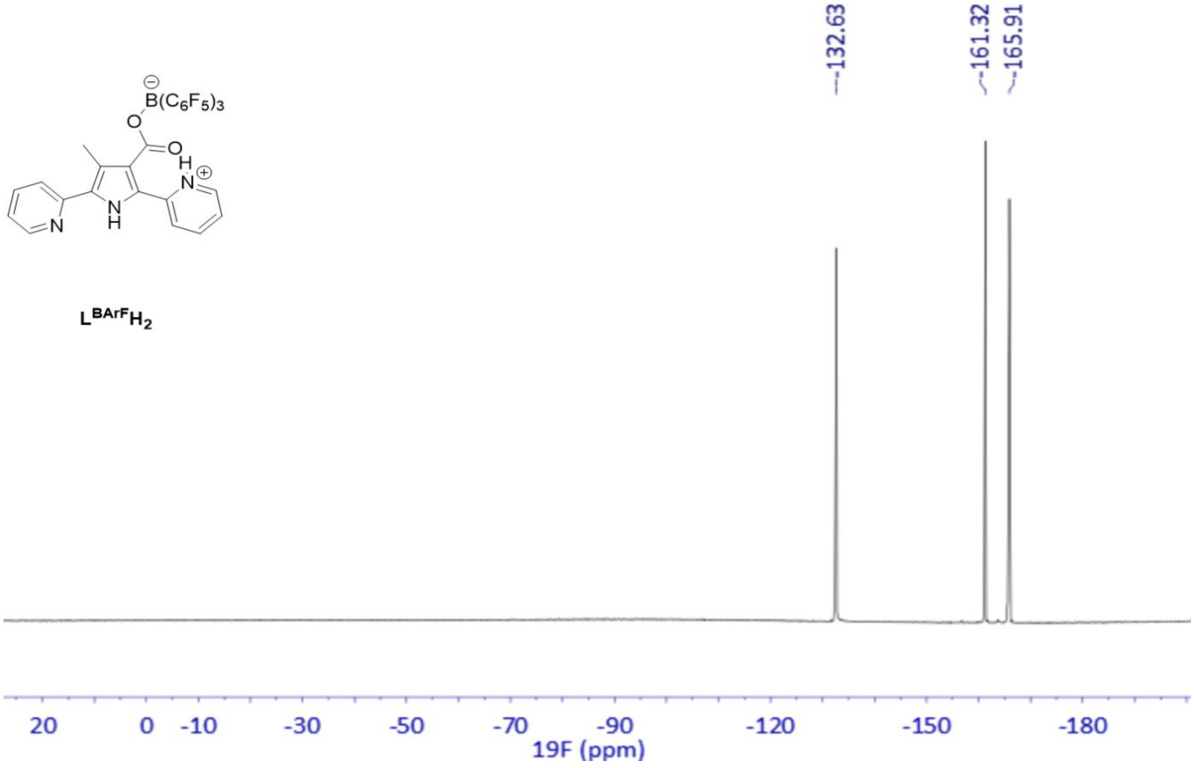

Figure S5. $^{19}F\{^{1}H\}$ NMR spectrum of $\mathbf{L^{BARF}H_2}$ in THF-d_8

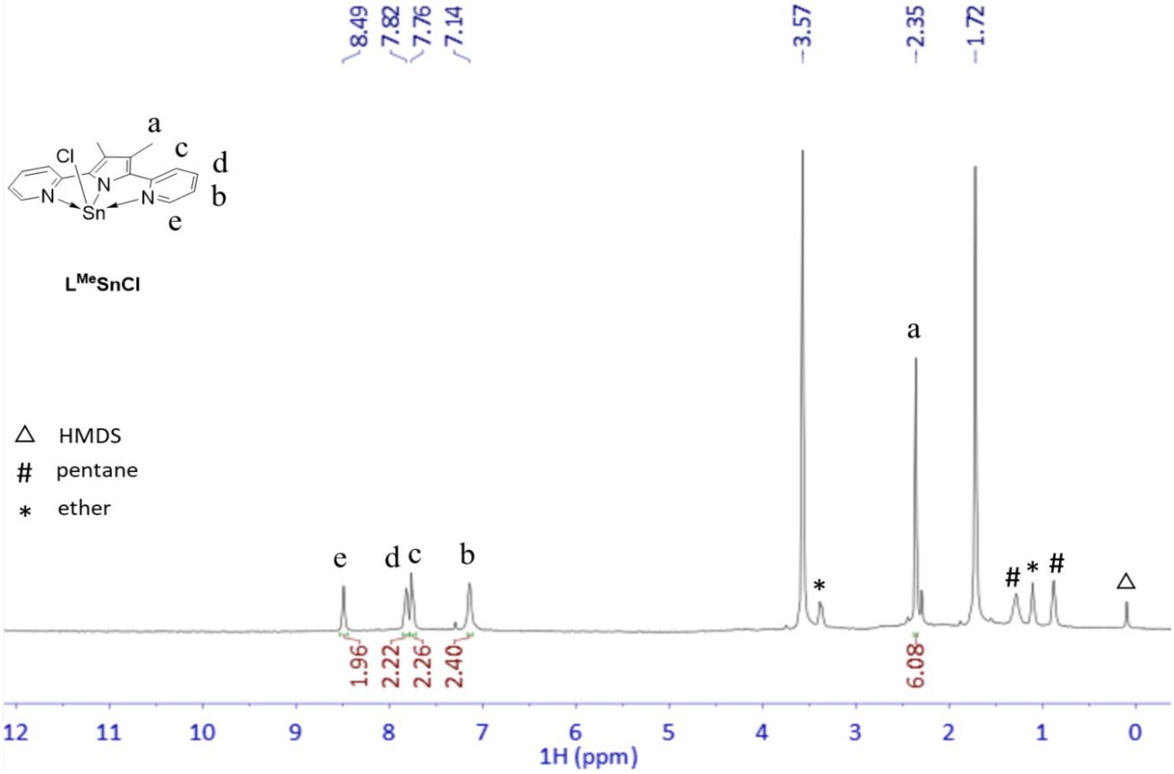

Figure S6. ^{1}H NMR spectrum of $\mathbf{L^{Me}SnCl}$ in THF-d_8

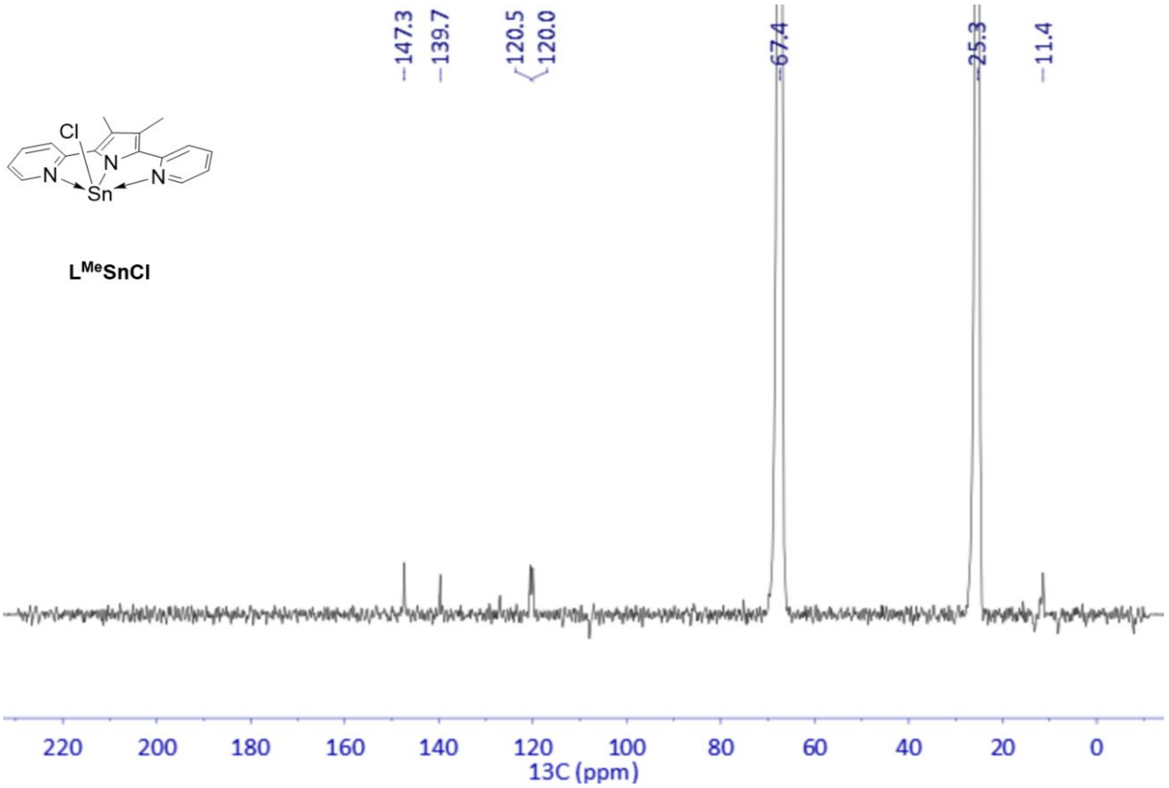

Figure S7. $^{13}C\{^{1}H\}$ NMR spectrum of $\mathbf{L^{Me}SnCl}$ in THF-d_8

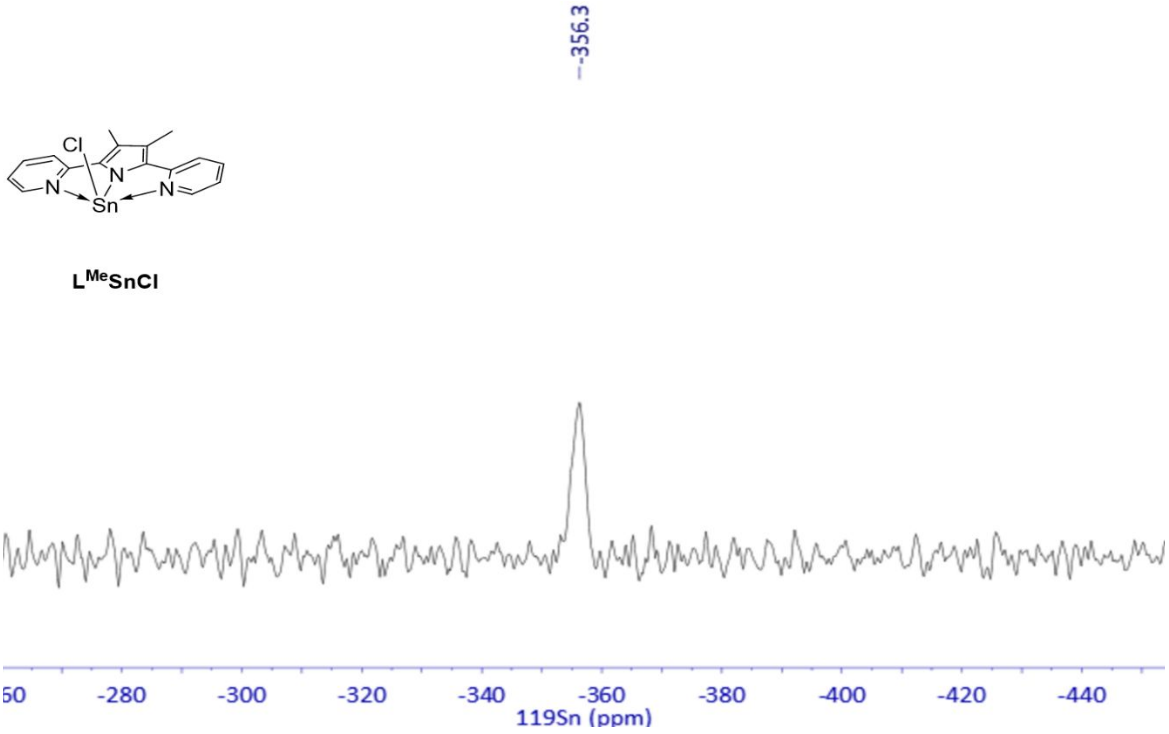

Figure S8. $^{119}Sn\{^{1}H\}$ NMR spectrum of $\mathbf{L^{Me}SnCl}$ in THF-d_8

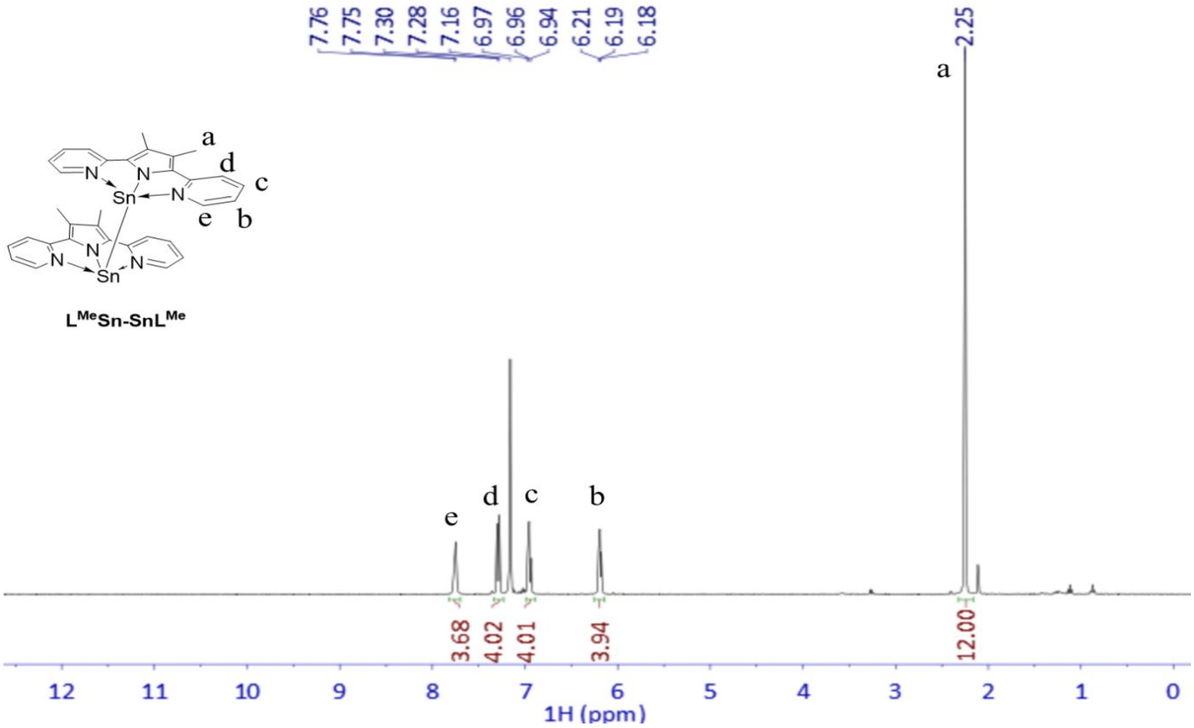

Figure S9. ^{1}H NMR spectrum of **L^{Me}Sn–SnL^{Me} (Sn$_2$)** in C_6D_6

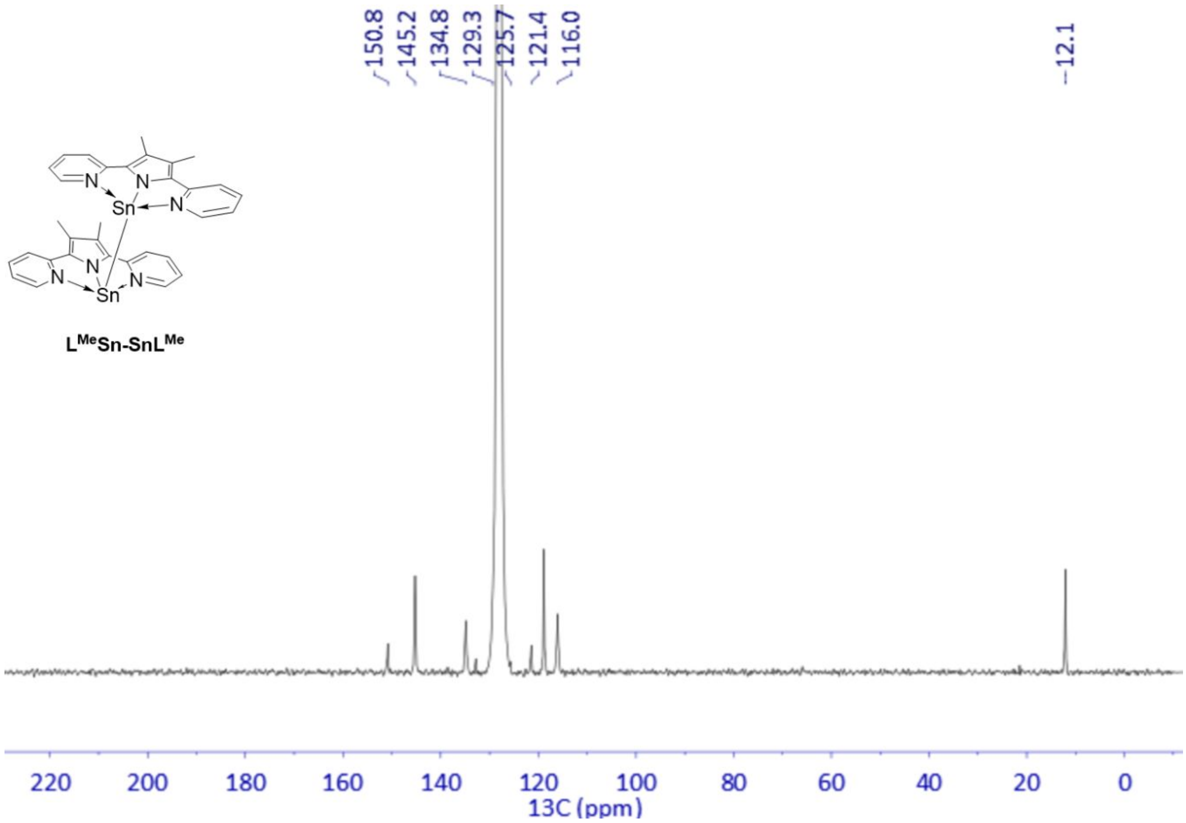

Figure S10. ^{13}C{^{1}H} NMR spectrum of **L^{Me}Sn–SnL^{Me} (Sn$_2$)** in C_6D_6

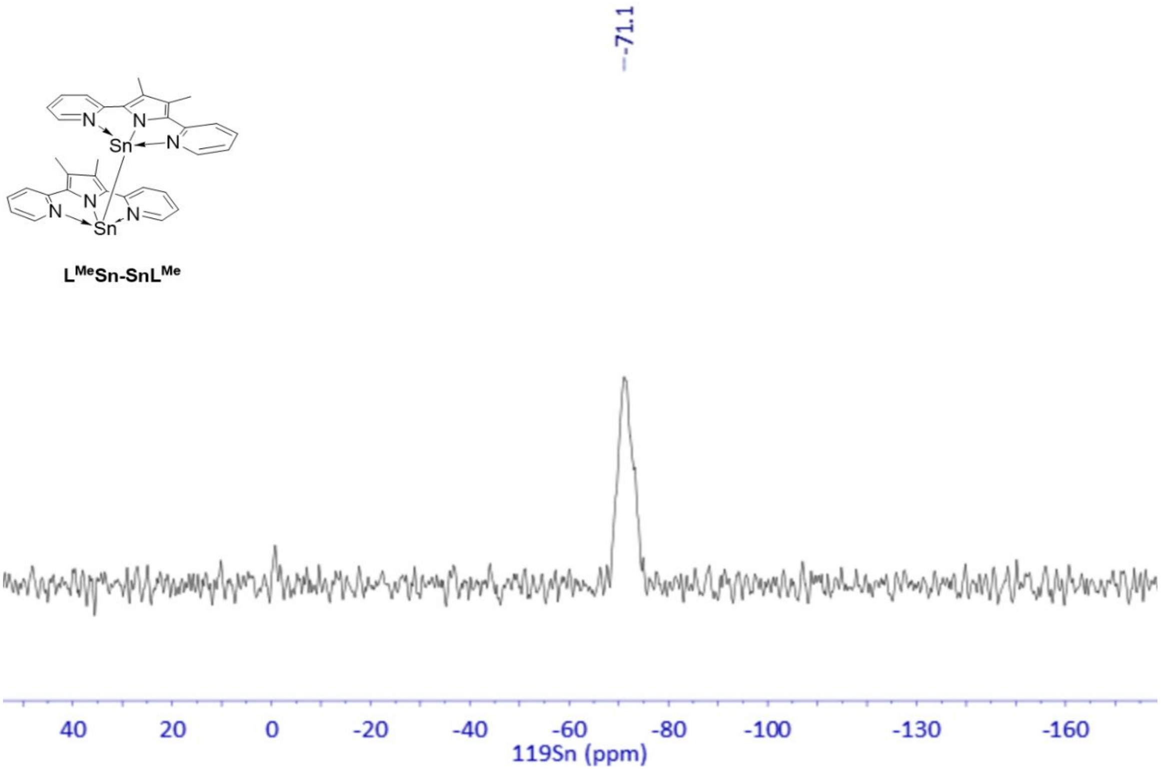

Figure S11: ^{119}Sn{^{1}H} NMR spectrum of **L^{Me}Sn–SnL^{Me} (Sn$_2$)** in THF-d_8

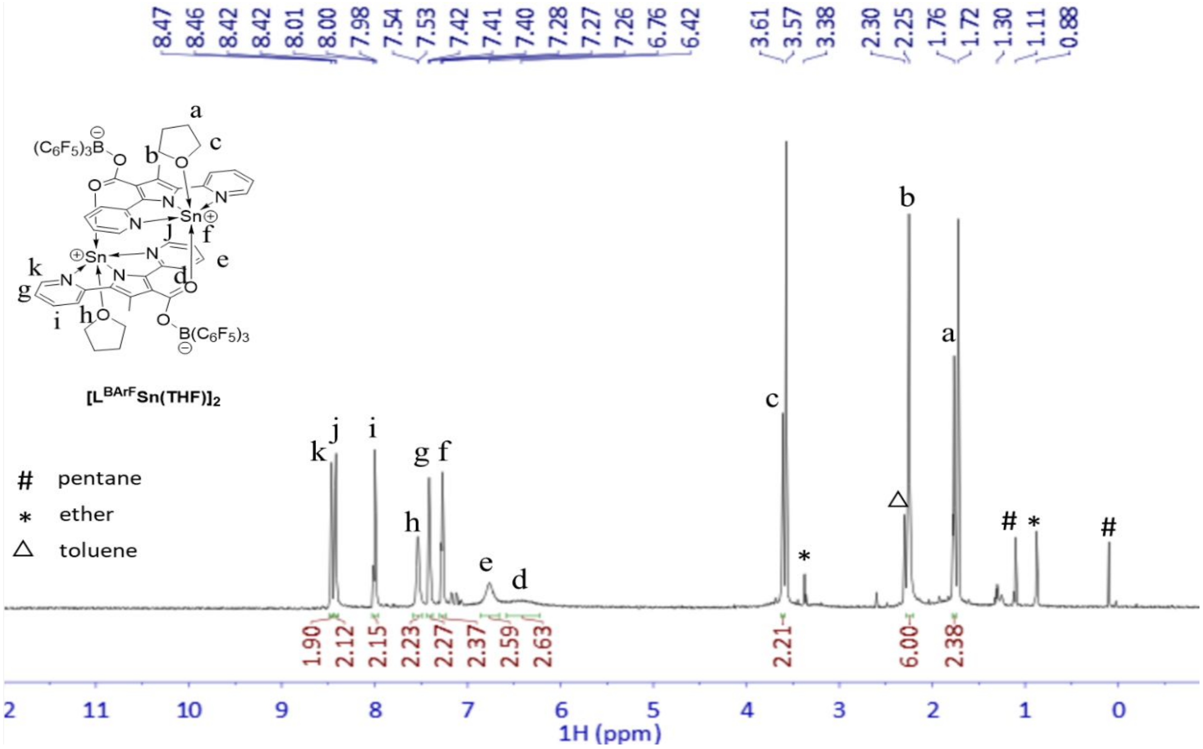

Figure S12. ^{1}H NMR spectrum of **[L^{BArF}Sn(THF)]$_2$ (L^{BArF}Sn)** in THF-d_8

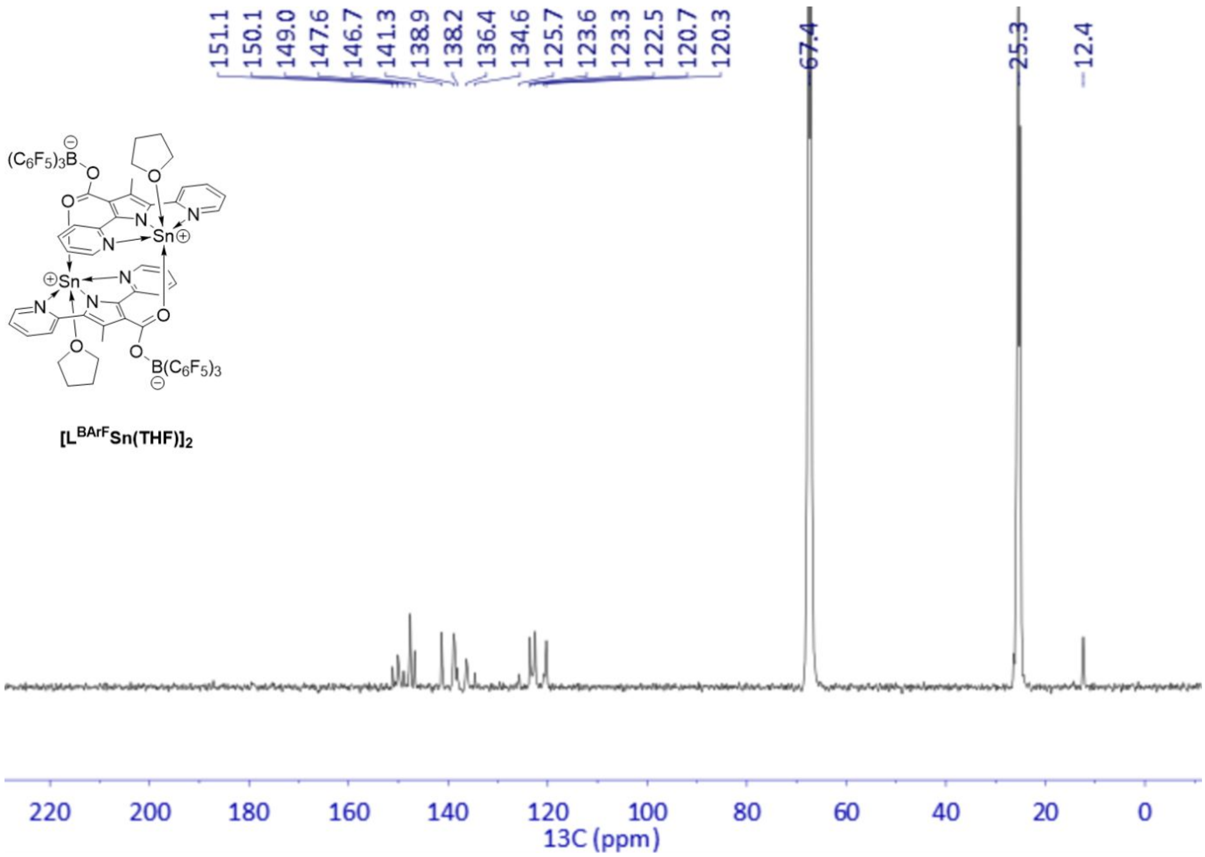

Figure S13. $^{13}C\{^{1}H\}$ NMR spectrum of **$[L^{BArF}Sn(THF)]_2$ ($L^{BArF}Sn$)** in THF-d_8

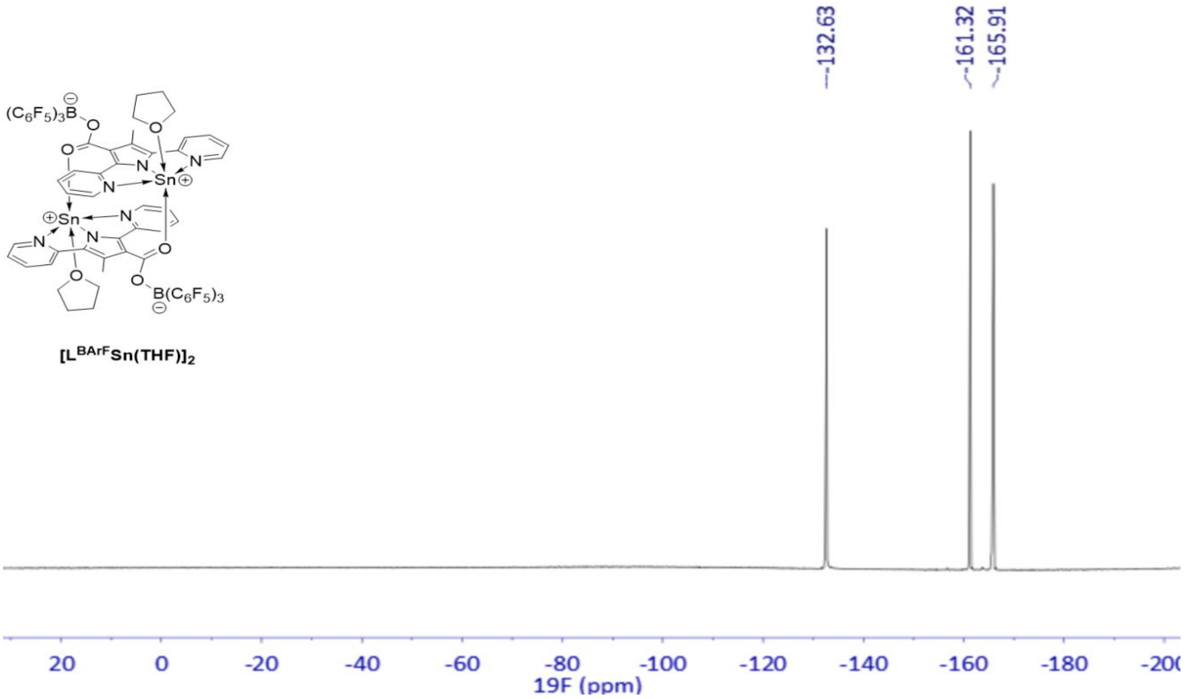

Figure S14. $^{19}F\{^{1}H\}$ NMR spectrum of **$[L^{BArF}Sn(THF)]_2$ ($L^{BArF}Sn$)** in THF-d_8

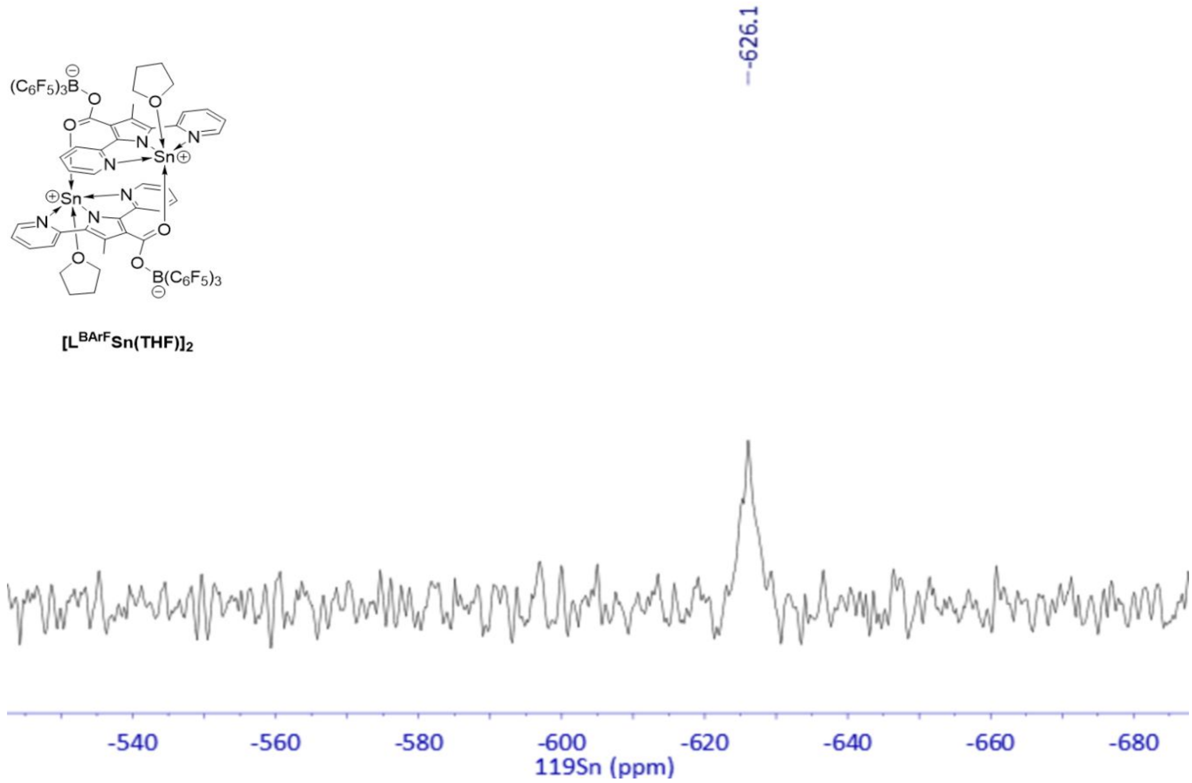

Figure S15. ^{119}Sn{^{1}H} NMR spectrum of **[L^{BArF}Sn(THF)]$_2$ (L^{BArF}Sn)** in THF-d_8

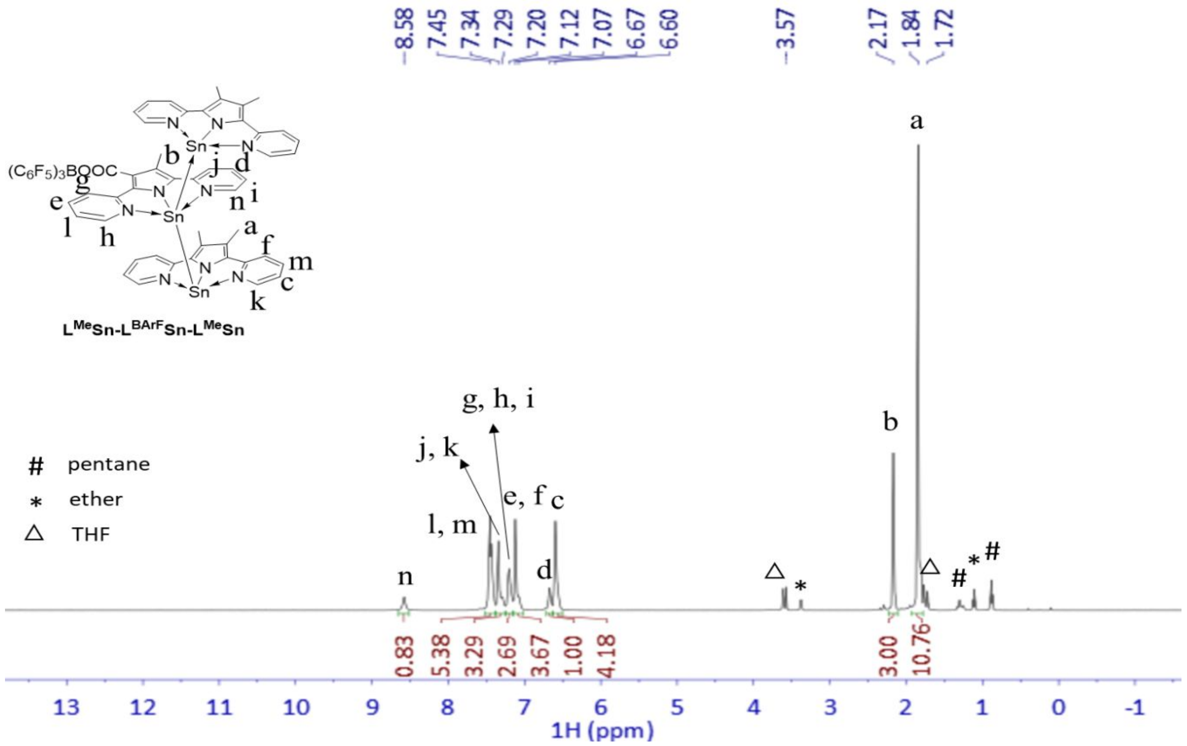

Figure S16. ^{1}H NMR spectrum of **L^{Me}Sn–L^{BArF}Sn–L^{Me}Sn (Sn$_3$)** in THF-d_8

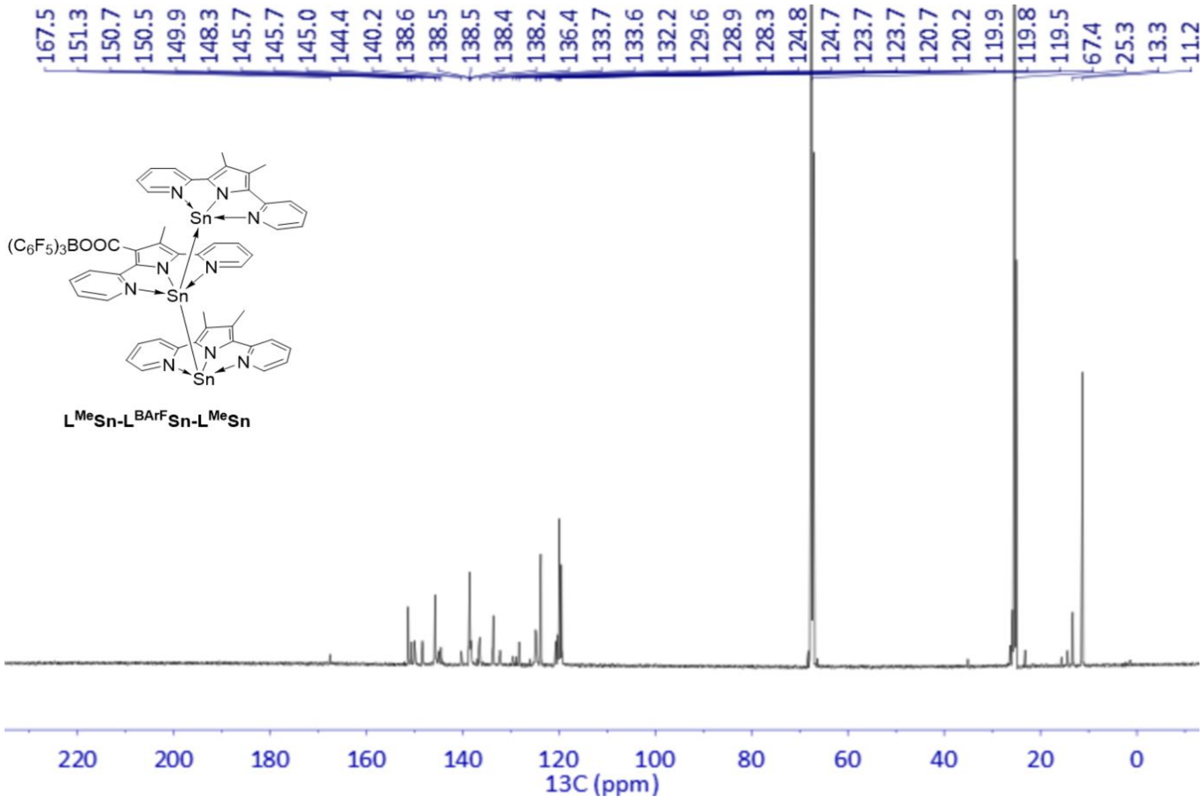

Figure S17. $^{13}C\{^{1}H\}$ NMR spectrum of **L^{Me}Sn–L^{BArF}Sn–L^{Me}Sn (Sn_3)** in THF-d_8

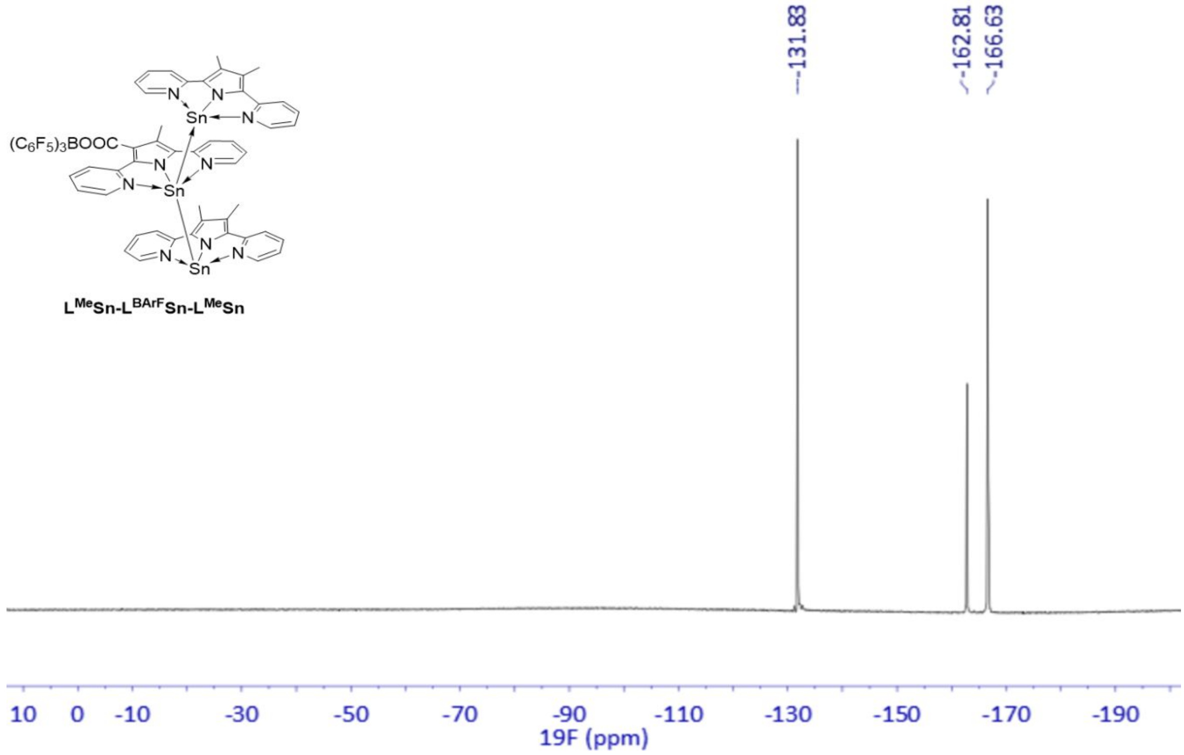

Figure S18. $^{19}F\{^{1}H\}$ NMR spectrum of of **L^{Me}Sn–L^{BArF}Sn–L^{Me}Sn (Sn_3)** in THF-d_8

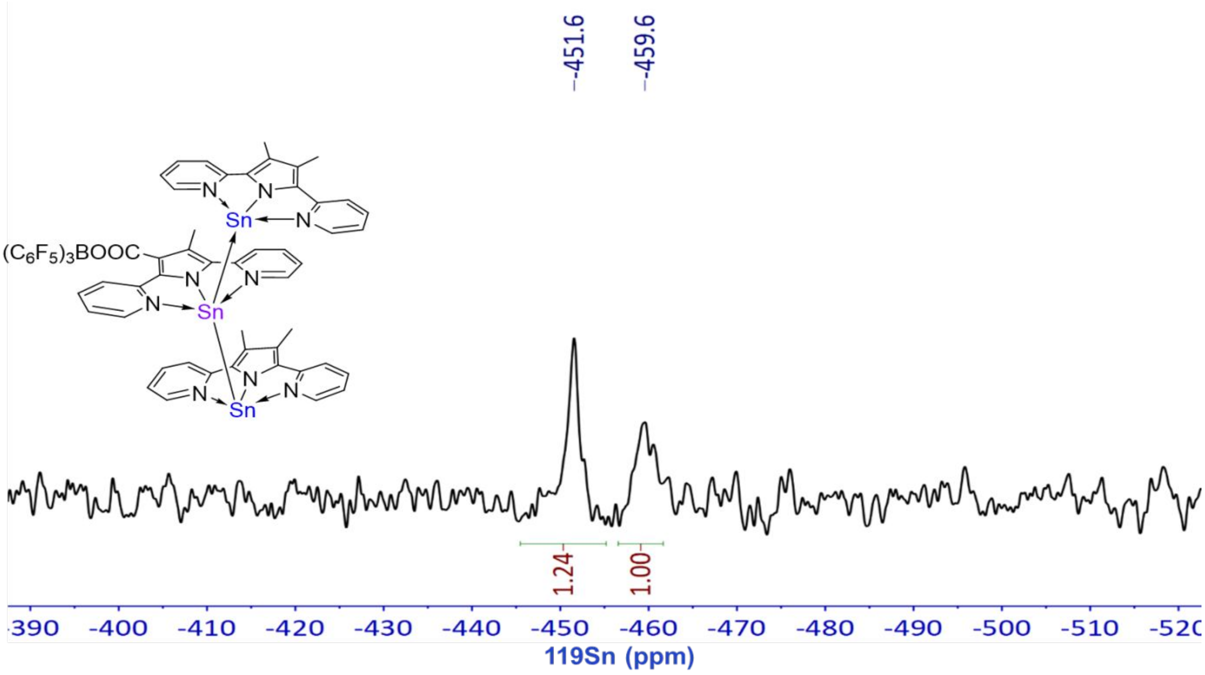

Figure S19. ^{119}Sn{^{1}H} NMR spectrum of **L^{Me}Sn–L^{BArF}Sn–L^{Me}Sn (Sn$_3$)** in THF-*d*$_8$

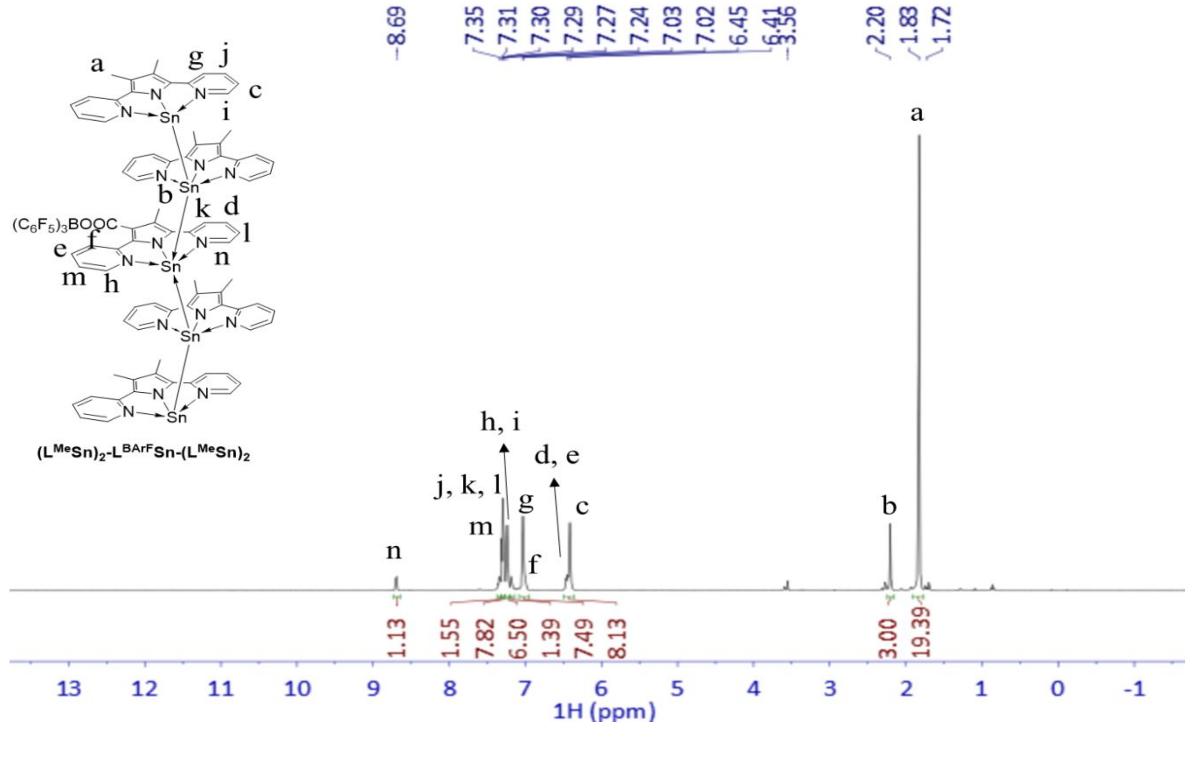

Figure S20. ^{1}H NMR spectrum of **(L^{Me}Sn)$_2$–L^{BArF}Sn–(L^{Me}Sn)$_2$ (Sn$_5$)** in THF-*d*$_8$

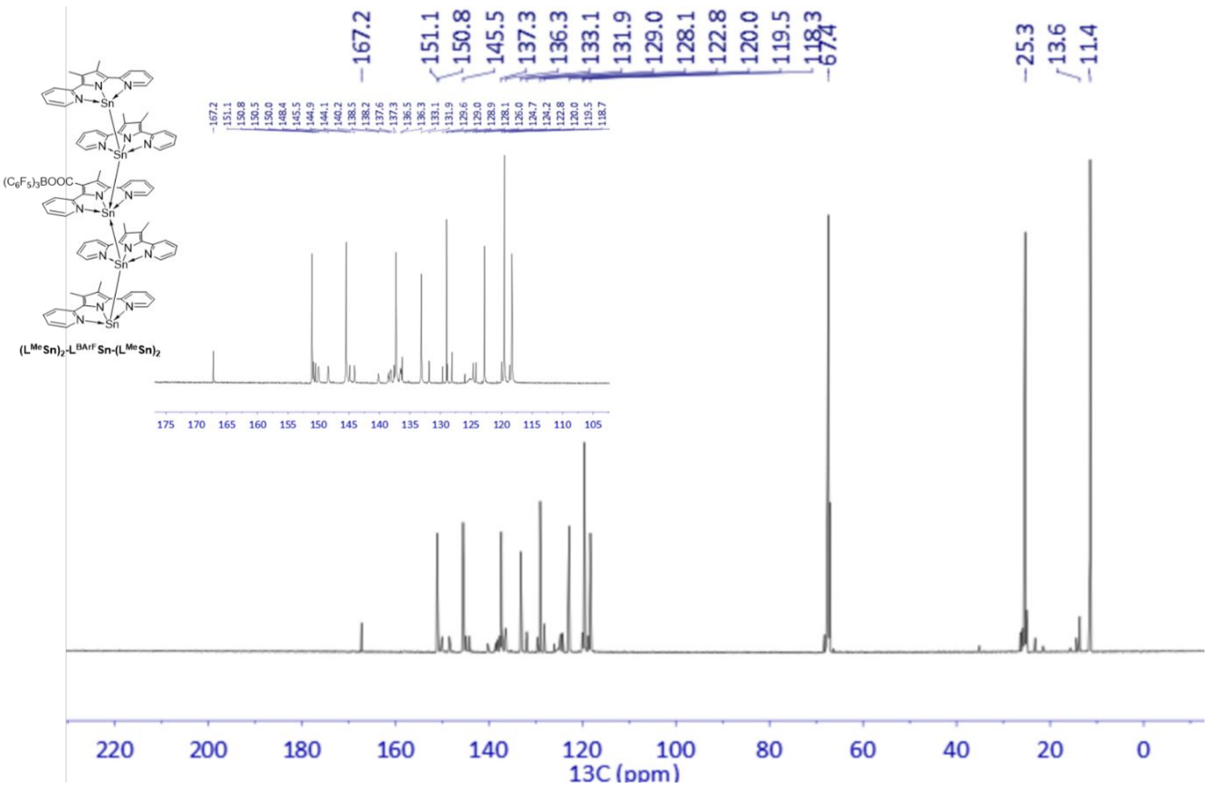

Figure S21. $^{13}C\{^{1}H\}$ NMR spectrum of **$(L^{Me}Sn)_2$–$L^{BArF}Sn$–$(L^{Me}Sn)_2$ (Sn_5)** in THF-d_8

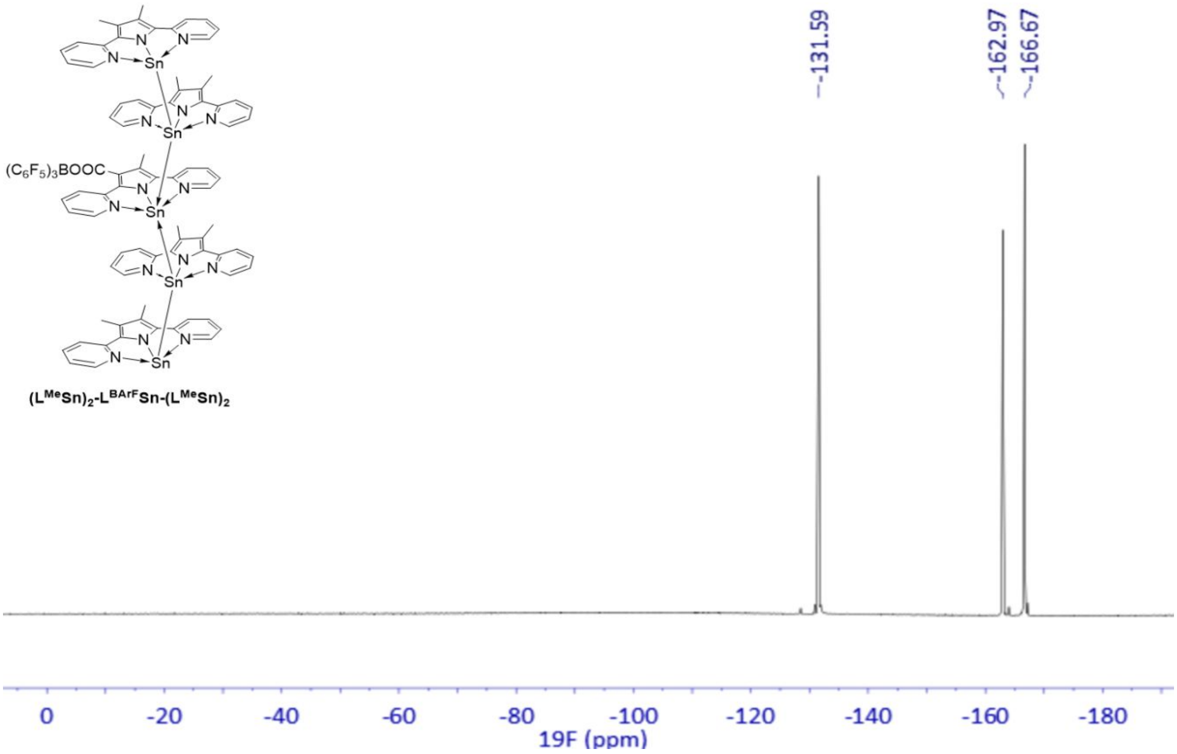

Figure S22. $^{19}F\{^{1}H\}$ NMR spectrum of of **$(L^{Me}Sn)_2$–$L^{BArF}Sn$–$(L^{Me}Sn)_2$ (Sn_5)** in THF-d_8

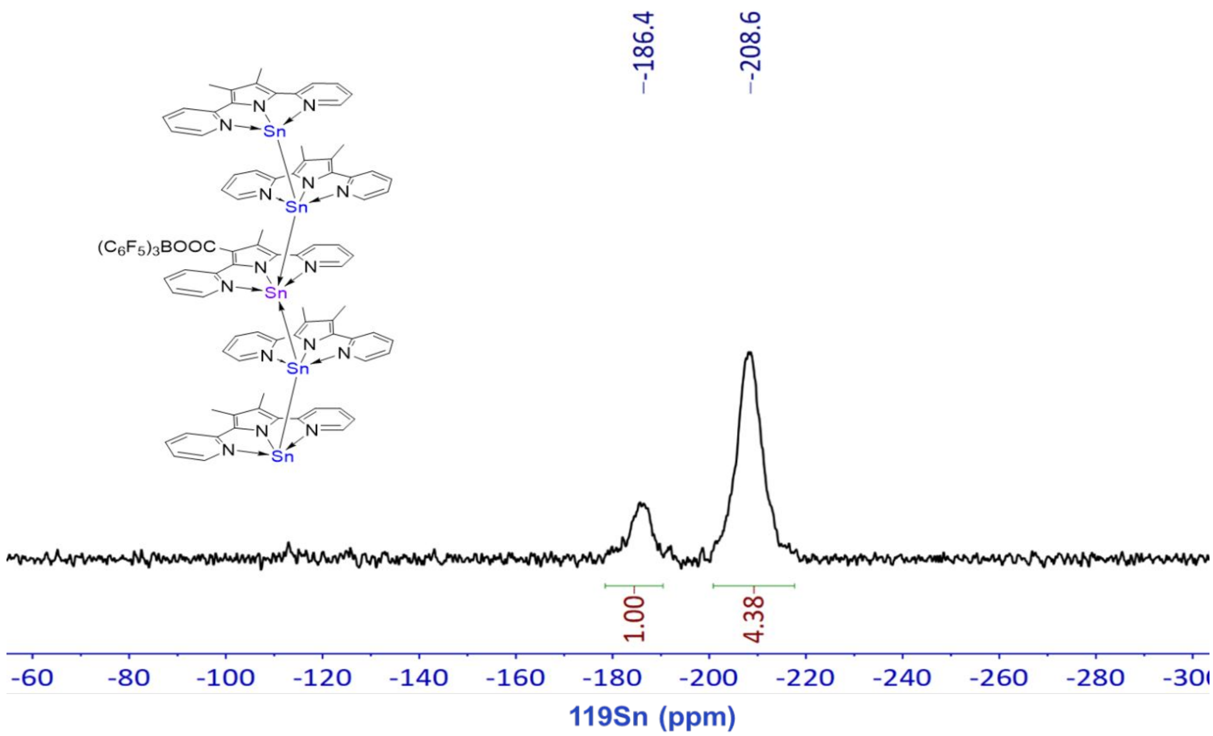

Figure S23. ^{119}Sn{^{1}H} NMR spectrum of **($L^{Me}Sn)_2$–$L^{BArF}Sn$–($L^{Me}Sn)_2$ (Sn_5)** in THF-d_8

2. Details on Raman measurement and data analysis

A laboratory constructed confocal Raman micro-spectrometer operating with a stabilized 532 nm laser (CW Nd:YVO_4; Verdi-V5, Coherent) was used for experiments (Fig. S24). The laser wavelength was stabilized using a high-resolution wavelength meter (WS-7, HighFinesse) along with a piezo-controller at 18789.995 (±0.003) cm^{-1}. The excitation beam was directed into an inverted microscope (iX71, Olympus) after passing through a Faraday isolator, laser line filter and a linear polarizer (Glan Taylor prism). A long working distance objective (20×, NA 0.25, *f* = 25 mm, Olympus SLMPLN20x) was used to focus laser beam inside the sample. Laser power of around 2 mW for all the samples. Backscattered light collected using the same objective lens passed through a confocal setup consisting of a 100 μm pinhole. After the confocal setup, Rayleigh scattering was removed using three Volume Bragg-notch filters (OptiGrate) allowing for low frequency Raman measurement down to 10 cm^{-1} checked using L-cysteine. Collimated beam of Raman scattered photons were focused on the polychromator slit (f=50 cm, *f*/6.5, 600 gr/mm grating, SP-2500i, Princeton Instruments, slit width=100 μm) using an achromatic convex lens. Spectral resolution was determined to be ~3.5 cm^{-1}. The spectra were recorded using a Peltier cooled CCD (DU970N-BV, Andor) operating at -85°C.

Wavenumber calibration was performed using emission lines from neon and vacuum wavelength data from Atomic Spectra Database, NIST. Collected Raman spectra were reduced by multiplying $\tilde{\nu}_0{}^{-1}(\tilde{\nu}_0 - \tilde{\nu}_s)^{-3}[1 - \exp(-hc\tilde{\nu}/k_B T)]$, in order to remove the contribution of the Boltzmann population and the frequency factor to the Raman intensities.

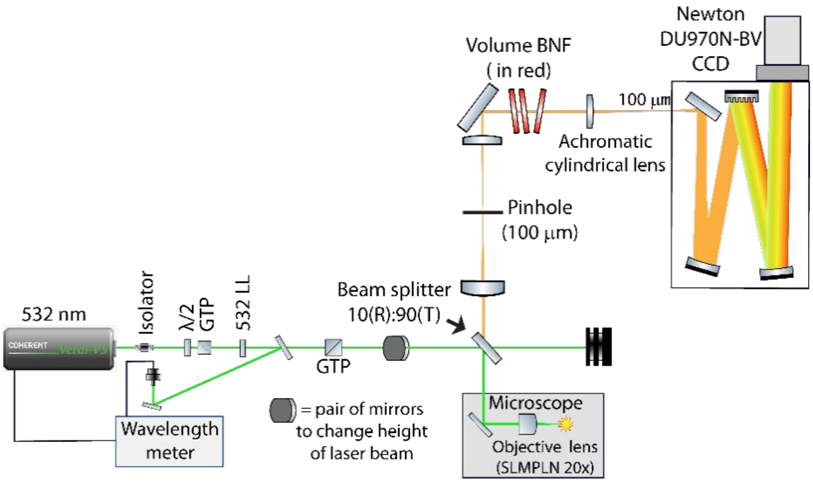

Figure S24. Optical setup of the Raman spectrometer used in the present work. In brief, a narrow band laser beam is directed in the microscope. Sample is placed on the microscope stage where it is exposed to the excitation laser. Back scattered Raman photons from the focal point are collected utilizing a confocal setup. Rayleigh scattering is rejected using narrow pass Bragg Notch Filters giving low wavenumber measurement capability. High sensitivity photon detection is performed using cooled CCD.

3. Single-crystal X-ray diffraction crystal structure figures and data tables

Single-crystal X-ray diffraction were performed on a Bruker APEX DUO diffractometer with APEX II 4K and multi-layer mirror monochromated Mo K_{α} radiation (λ = 0.71073 Å) at 200(2) K. Data collection and reduction were performed with Bruker APEX II software. All of non-hydrogen atoms are refined anisotropically. Hydrogen atoms attached to the carbons were fixed at calculated positions and refined using a riding mode. Multiple disordered solvent molecules were observed in the crystal structures of all complexes. Whenever possible, co-crystallizing solvent molecules were modeled. Otherwise, SQUEEZE was employed to treat diffuse solvent contribution in the voids.

Four cif files have been deposited on CCDC (2526629 ([L^{BarF}Sn(THF)]$_2$, **L^{BarF}Sn**), 2526632 (L^{MeCOOH}H), 2526633 (L^{Me}Sn–SnL^{Me}, **Sn$_2$**), 2526634 (L^{Me}SnCl)). Owing to the limited quality of the diffraction data, the crystal structures of $L^{BArF}H_2$ and [(L^{Ph}Sn)$_3$][L^{BArF}SnCl] are provided in the Supporting Information, and the corresponding cif files are included therein.

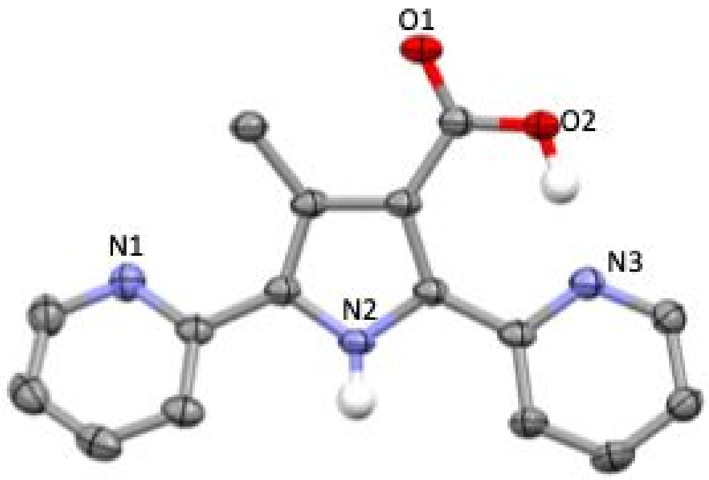

Figure S25. ORTEP of **L^{MeCOOH}H.** (50% thermal ellipsoids; hydrogen atoms omitted for clarity). Selected interatomic distances (Å): C(10)-O(1) 1.235(2), C(10)-O(2) 1.299(2), O(2)-N(3) 2.504.

Table S1. Crystal data and structure refinement for **L^{MeCOOH}H** (CCDC 2526632)

Empirical formula	$C_{16}H_{13}N_3O_2$	
Formula weight	279.29	
Temperature	200(2) K	
Wavelength	0.71073 Å	
Crystal system	Monoclinic	
Space group	P 21/n	
Unit cell dimensions	a = 10.7131(7) Å	α = 90°.
	b = 9.2890(6) Å	β = 107.985(2)°.
	c = 13.6526(9) Å	γ = 90°.
Volume	1292.24(15) Å^3	
Z	4	
Density (calculated)	1.436 Mg/m^3	
Absorption coefficient	0.098 mm^{-1}	
F(000)	584	
Crystal size	0.180 x 0.130 x 0.030 mm^3	
Theta range for data collection	2.696 to 25.059°.	
Index ranges	-12≤h≤12, -11≤k≤11, -16≤l≤16	
Reflections collected	19555	
Independent reflections	2273 [R(int) = 0.0639]	
Completeness to theta = 25.059°	99.4 %	
Refinement method	Full-matrix least-squares on F^2	
Data / restraints / parameters	2273 / 0 / 191	
Goodness-of-fit on F^2	1.111	
Final R indices [I>2sigma(I)]	R1 = 0.0413, wR2 = 0.0915	
R indices (all data)	R1 = 0.0627, wR2 = 0.1092	

Extinction coefficient	n/a
Largest diff. peak and hole	0.186 and -0.211 e.$Å^{-3}$

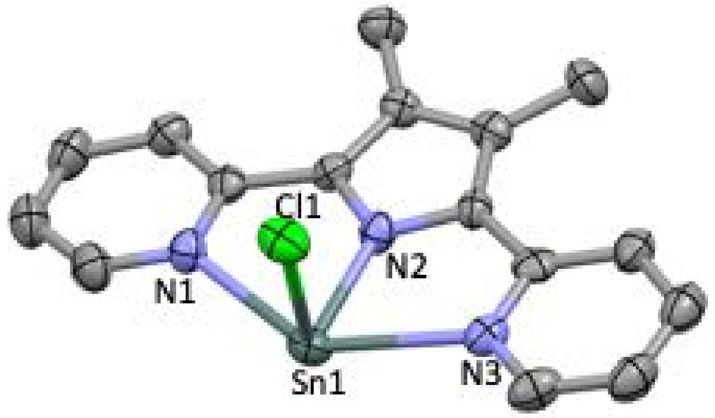

Figure S26. ORTEP of **L^{Me}SnCl.** (50% thermal ellipsoids; hydrogen atoms omitted for clarity). Selected interatomic distances (Å): N(2)-Sn(1) 2.131(3), N(1)-Sn(1) 2.492(4), N(3)-Sn(1) 2.481(4), Sn(1)-Cl(1) 2.493(12). N(2)–Sn(1)–Cl(1) 93.18(10).

Table S2. Crystal data and structure refinement for **L^{Me}SnCl** (CCDC 2526634)

Empirical formula	C16 H14 Cl N3 Sn	
Formula weight	402.44	
Temperature	200(2) K	
Wavelength	0.71073 Å	
Crystal system	Monoclinic	
Space group	C 2/c	
Unit cell dimensions	a = 21.1303(18) Å	α = 90°.
	b = 8.5648(7) Å	β = 90.315(3)°.
	c = 19.9597(19) Å	γ = 90°.
Volume	3612.2(5) Å^3	
Z	8	
Density (calculated)	1.480 Mg/m^3	
Absorption coefficient	1.559 mm^{-1}	
F(000)	1584	
Crystal size	0.07 x 0.06 x 0.01 mm^3	
Theta range for data collection	2.76 to 25.06°.	
Index ranges	-25≤h≤25, -10≤k≤9, -23≤l≤23	
Reflections collected	23349	
Independent reflections	3205 [R(int) = 0.0654]	
Completeness to theta = 25.06°	99.8 %	
Absorption correction	multi-scan	
Max. and min. transmission	0.9846 and 0.8987	
Refinement method	Full-matrix least-squares on F^2	
Data / restraints / parameters	3205 / 0 / 192	
Goodness-of-fit on F^2	1.046	
Final R indices [I>2sigma(I)]	R1 = 0.0366, wR2 = 0.0856	

R indices (all data)	R1 = 0.0515, wR2 = 0.0911
Largest diff. peak and hole	0.367 and -0.515 e.Å^{-3}

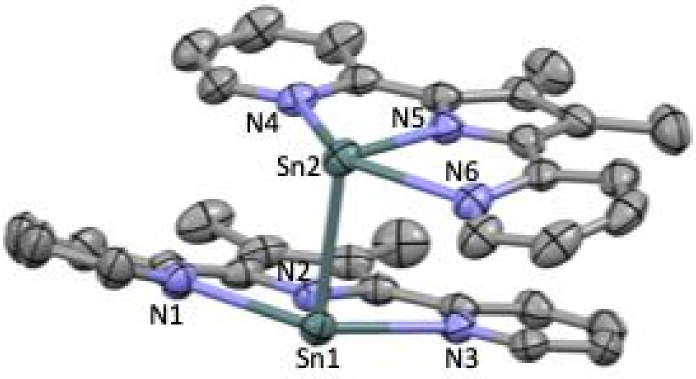

Figure S27. ORTEP of **L^{Me}Sn–SnL^{Me} (Sn_2).** (50% thermal ellipsoids; hydrogen atoms omitted for clarity). Selected interatomic distances (Å) and angles (°): Sn(1)-Sn(2) 2.9537(4), N(2)-Sn(1) 2.150(3), N(5)-Sn(2) 2.135(3), N(2)-Sn(1)-Sn(2) 96.10(8), N(5)-Sn(2)-Sn(1) 96.33(8).

Table S3. Crystal data and structure refinement for **(L^{Me}Sn)$_2$** (CCDC 2526633)

Empirical formula	$C_{32}H_{28}N_6Sn_2$	
Formula weight	733.98	
Temperature	200(2) K	
Wavelength	0.71073 Å	
Crystal system	Monoclinic	
Space group	C 2/c	
Unit cell dimensions	a = 34.4902(17) Å	α = 90°.
	b = 9.7951(4) Å	β = 99.4960(10)°.
	c = 17.0466(6) Å	γ = 90°.
Volume	5680.0(4) Å^3	
Z	8	
Density (calculated)	1.717 Mg/m^3	
Absorption coefficient	1.792 mm^{-1}	
F(000)	2896	
Crystal size	0.470 x 0.070 x 0.020 mm^3	
Theta range for data collection	2.164 to 25.032°.	
Index ranges	-40≤h≤40, -11≤k≤11, -20≤l≤18	
Reflections collected	40509	
Independent reflections	5017 [R(int) = 0.0719]	
Completeness to theta = 25.032°	99.8 %	
Refinement method	Full-matrix least-squares on F^2	
Data / restraints / parameters	5017 / 0 / 365	
Goodness-of-fit on F^2	1.040	
Final R indices [I>2sigma(I)]	R1 = 0.0289, wR2 = 0.0584	
R indices (all data)	R1 = 0.0487, wR2 = 0.0674	
Extinction coefficient	n/a	
Largest diff. peak and hole	0.475 and -0.555 e.Å^{-3}	

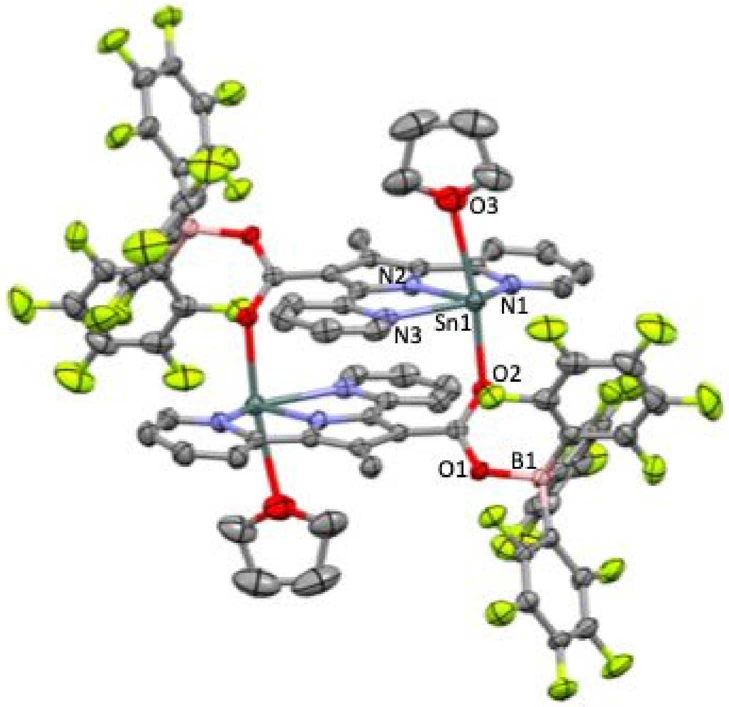

Figure S28. ORTEP of **[L^{BArF}Sn(THF)]$_2$**. (**L^{BArF}Sn**) (50% thermal ellipsoids; hydrogen atoms omitted for clarity). Selected interatomic distances (Å) and angles (°): N(1)-Sn(1) 2.425(2), N(2)-Sn(1) 2.145(2), N(3)-Sn(1) 2.479(2), O(2)-Sn(1) 2.3059(18), O(3)-Sn(1) 2.700, C(10)-O(2) 1.255(3), C(10)-O(1) 1.295(3), N(2)-Sn(1)-O(2) 92.68(8), C(10)-O(2)-Sn(1) 130.40(16).

Table S4. Crystal data and structure refinement for **[L^{BArF}Sn(THF)]$_2$** (CCDC 2526629)

Empirical formula	$C_{38}H_{19}BF_{15}N_3O_3Sn$	
Formula weight	980.06	
Temperature	200(2) K	
Wavelength	0.71073 Å	
Crystal system	Monoclinic	
Space group	P 21/n	
Unit cell dimensions	a = 9.9048(4) Å	α = 90°.
	b = 18.2845(6) Å	β = 94.1580(10)°.
	c = 21.4336(7) Å	γ = 90°.
Volume	3871.5(2) Å3	
Z	4	
Density (calculated)	1.681 Mg/m^3	
Absorption coefficient	0.774 mm^{-1}	
F(000)	1928	
Crystal size	0.120 x 0.020 x 0.010 mm^3	
Theta range for data collection	1.905 to 25.068°.	
Index ranges	-11≤h≤11, -21≤k≤21, -25≤l≤25	
Reflections collected	78741	
Independent reflections	6862 [R(int) = 0.0653]	

Completeness to theta = 25.068°	99.9 %
Refinement method	Full-matrix least-squares on F^2
Data / restraints / parameters	6862 / 0 / 551
Goodness-of-fit on F^2	1.156
Final R indices [I>2sigma(I)]	R1 = 0.0287, wR2 = 0.0808
R indices (all data)	R1 = 0.0438, wR2 = 0.1014
Extinction coefficient	n/a
Largest diff. peak and hole	0.578 and -0.632 e.Å^{-3}

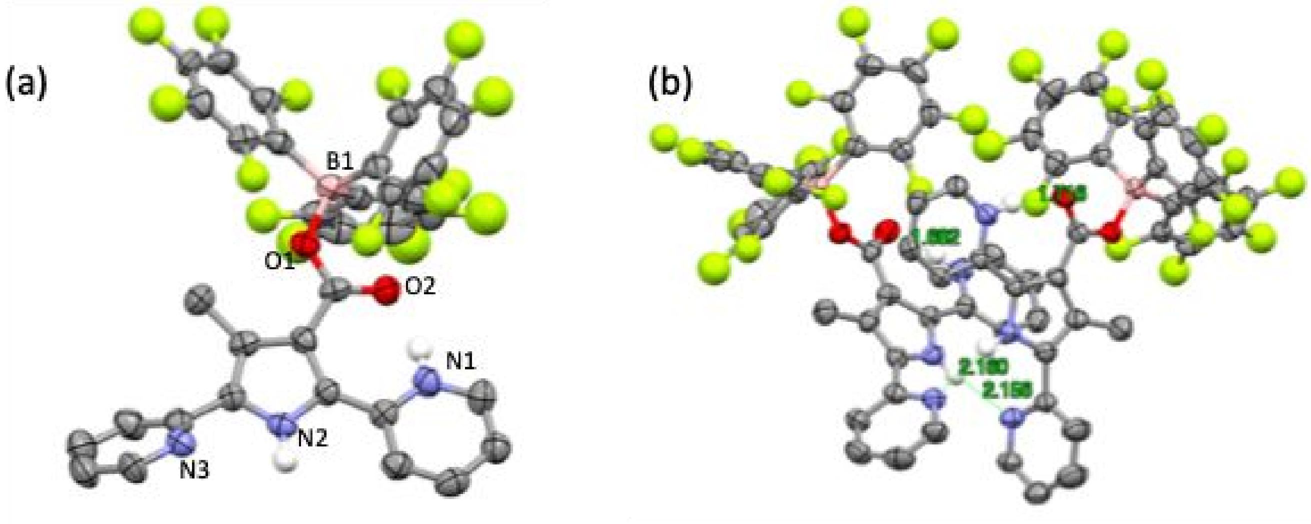

Figure S29. ORTEP of **$L^{BArF}H_2$.** (50% thermal ellipsoids; hydrogen atoms on carbons and one Et_2O molecule omitted for clarity). (a) One of the two crystallographically independent $L^{BArF}H_2$ molecules present in the unit cell. Selected interatomic distances (Å): C(1)–O(2) 1.250(6), C(1)–O(1) 1.294(6), O(2)···N(1) 2.538. (b) View showing the two $L^{BArF}H_2$ molecules in the unit cell with intermolecular hydrogen-bonding interactions between them.

Table S5. Crystal data and structure refinement for **$L^{BArF}H_2$**

Empirical formula	$C_{72}H_{36}B_2F_{30}N_6O_5$	
Formula weight	1656.69	
Temperature	200(2) K	
Wavelength	0.71073 Å	
Crystal system	Monoclinic	
Space group	P 21/n	
Unit cell dimensions	a = 17.9836(8) Å	α = 90°.
	b = 27.5436(13) Å	β = 96.2750(10)°.
	c = 18.5815(8) Å	γ = 90°.
Volume	9148.9(7) $Å^3$	
Z	4	
Density (calculated)	1.203 Mg/m^3	
Absorption coefficient	0.118 mm^{-1}	
F(000)	3320	
Crystal size	0.42 x 0.18 x 0.06 mm^3	
Theta range for data collection	2.10 to 25.06°.	
Index ranges	-20≤h≤21, -32≤k≤32, -22≤l≤22	
Reflections collected	135891	
Independent reflections	16195 [R(int) = 0.0962]	
Completeness to theta = 25.06°	99.8 %	
Absorption correction	multi-scan	
Max. and min. transmission	0.9930 and 0.9523	
Refinement method	Full-matrix least-squares on F^2	

Data / restraints / parameters	16195 / 0 / 890
Goodness-of-fit on F^2	1.213
Final R indices [I>2sigma(I)]	R1 = 0.1113, wR2 = 0.3319
R indices (all data)	R1 = 0.1645, wR2 = 0.3660
Largest diff. peak and hole	0.865 and -0.790 e.Å-3

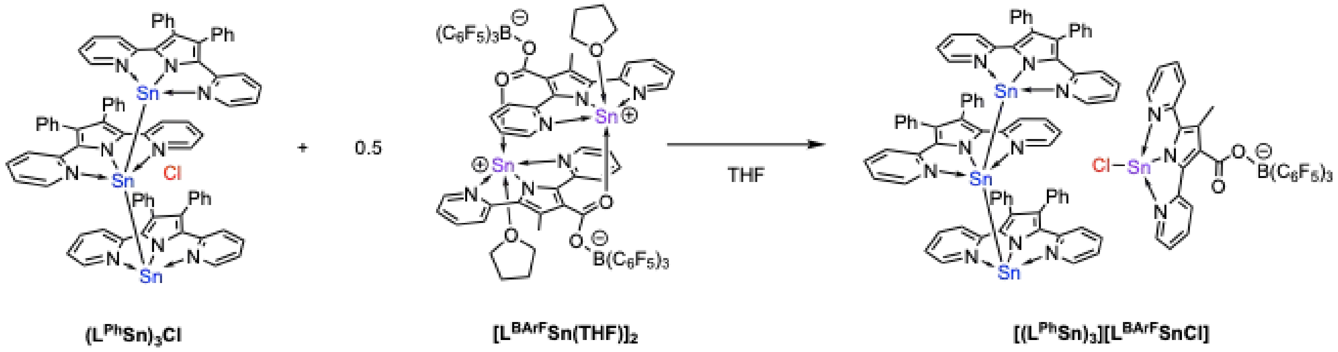

Scheme S1. The reaction of $(L^{Ph}Sn)_3Cl$ with $\mathbf{L^{BArF}Sn}$ in THF.

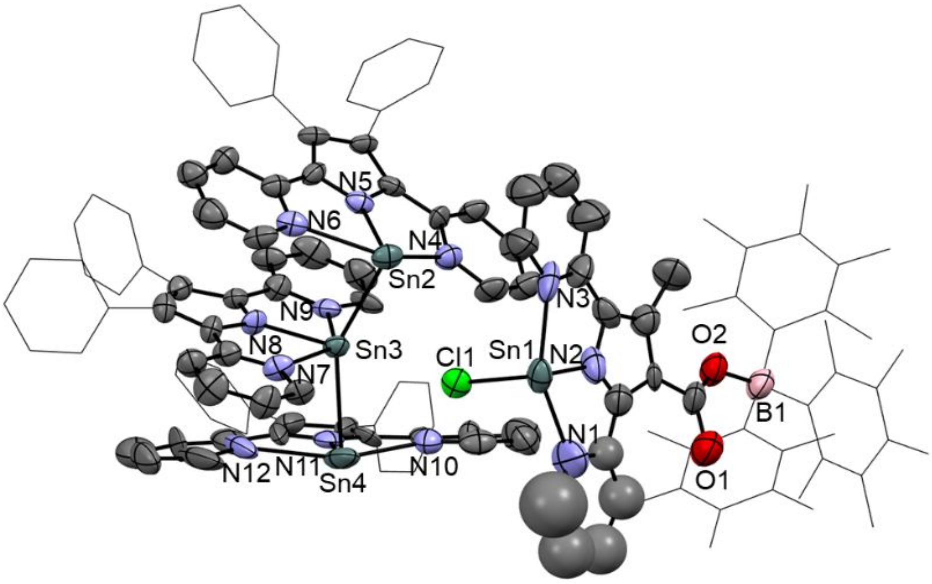

Figure S30. ORTEP of $[(L^{Ph}Sn)_3][L^{BArF}SnCl]$**.** (50% thermal ellipsoids; hydrogen atoms omitted for clarity).

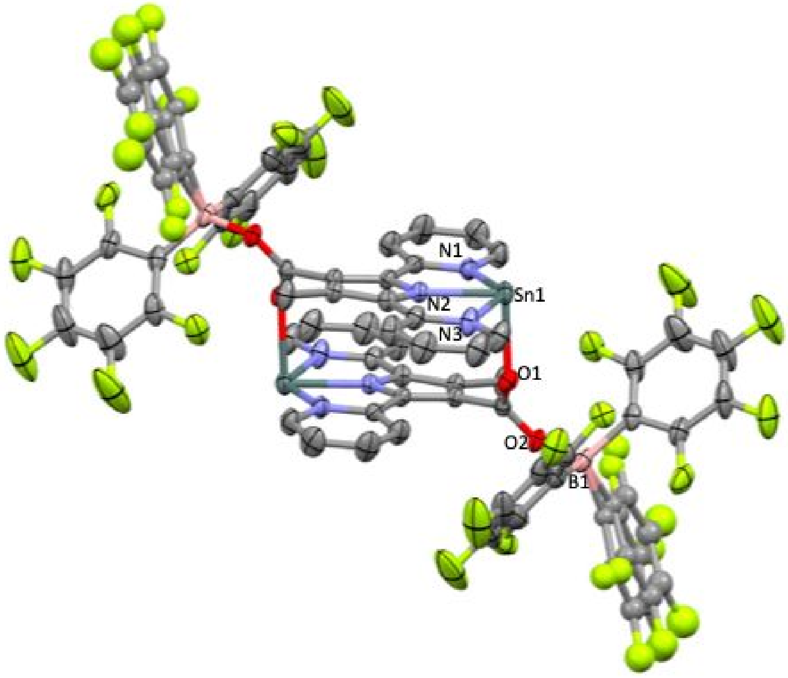

Figure S31. ORTEP of **($L^{BArF}Sn)_2$**. (50% thermal ellipsoids; hydrogen atoms omitted for clarity). Selected interatomic distances (Å) and angles (°): N(1)-Sn(1) 2.479(3), N(2)-Sn(1) 2.137(3), N(3)-Sn(1) 2.432(3), O(2)-Sn(1) 2.3059(18), O(1)-Sn(1) 2.276(2), C(8)-O(1) 1.245(4), C(8)-O(2) 1.289(4), N(2)-Sn(1)-O(1) 92.48(10).

Table S6. Crystal data and structure refinement for **($L^{BArF}Sn)_2$**

Empirical formula	$C_{68}H_{22}B_2F_{30}N_6O_4Sn_2$	
Formula weight	1815.92	
Temperature	200(2) K	
Wavelength	0.71073 Å	
Crystal system	Monoclinic	
Space group	P 21/c	
Unit cell dimensions	a = 12.5829(5) Å	α= 90°.
	b = 18.7830(8) Å	β= 106.6190(10)°.
	c = 19.4852(8) Å	γ = 90°.
Volume	4412.9(3) Å^3	
Z	2	
Density (calculated)	1.367 Mg/m^3	
Absorption coefficient	0.672 mm^{-1}	
F(000)	1768	
Crystal size	0.44 x 0.42 x 0.23 mm^3	
Theta range for data collection	2.43 to 25.07°.	
Index ranges	-14≤h≤14, -22≤k≤22, -22≤l≤23	
Reflections collected	83133	

Independent reflections	7791 [R(int) = 0.0507]
Completeness to theta = 25.07°	99.5 %
Absorption correction	multi-scan
Max. and min. transmission	0.8608 and 0.7565
Refinement method	Full-matrix least-squares on F^2
Data / restraints / parameters	7791 / 11 / 494
Goodness-of-fit on F2	1.052
Final R indices [I>2sigma(I)]	R1 = 0.0465, wR2 = 0.1191
R indices (all data)	R1 = 0.0522, wR2 = 0.1231
Largest diff. peak and hole	0.968 and -0.718 e.Å^{-3}

4. X-ray Absorption Spectroscopy

X-ray absorption spectroscopy (XAS) measurements, including X-ray absorption near edge spectra (XANES) and extended X-ray absorption fine structure (EXAFS) at the Sn K-edge, were performed using a Lytle detector at the 01C1 beamline of the Taiwan Light Source (TLS), National Synchrotron Radiation Research Center (NSRRC). The samples were sealed in airtight tubes under an argon atmosphere during data collection. The pre-edge baseline was subtracted, and the spectra were normalized to the post-edge region. EXAFS analysis was conducted by Fourier transforming the k^3-weighted EXAFS oscillations to evaluate the contribution of different coordination shells to the Fourier transform peaks.

(a)

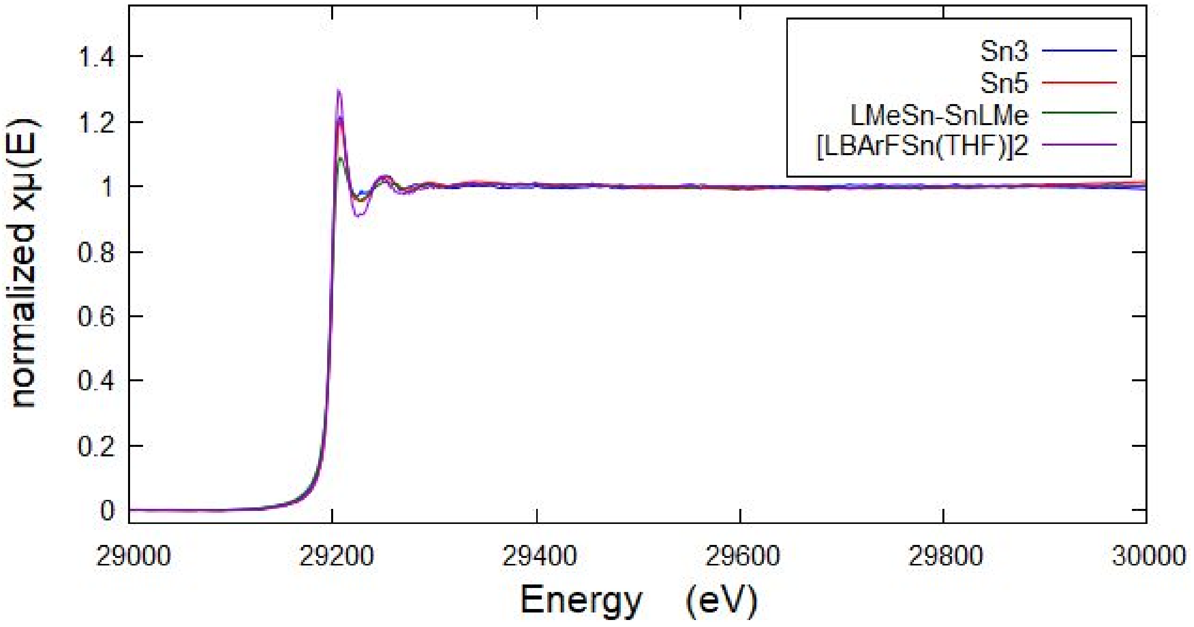

(b)

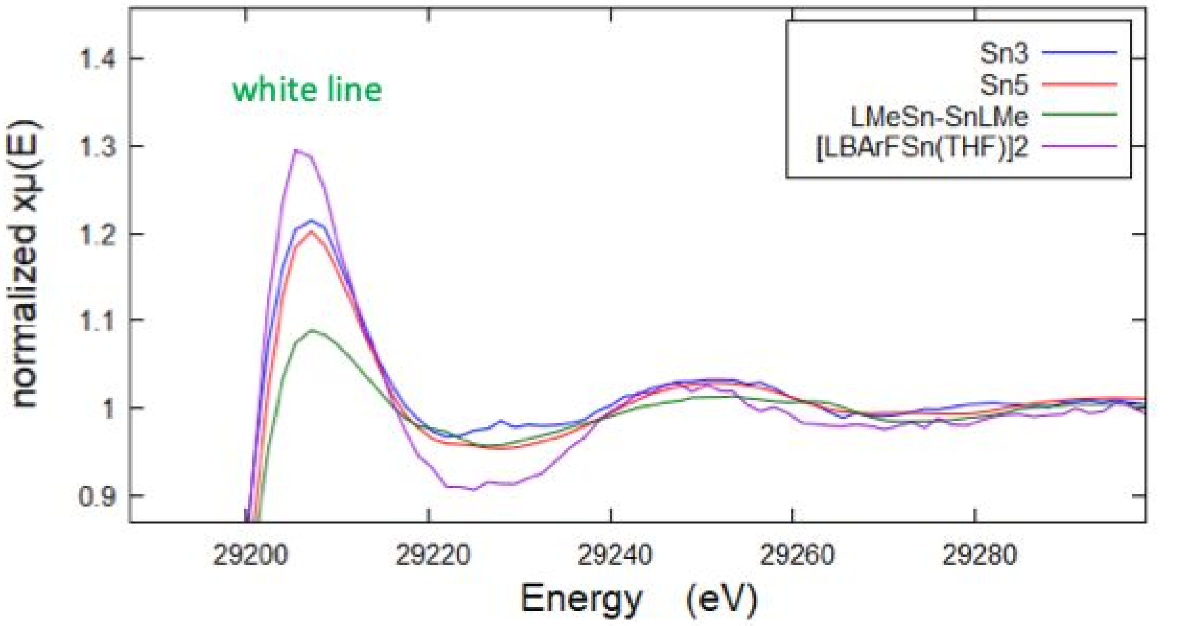

(c)

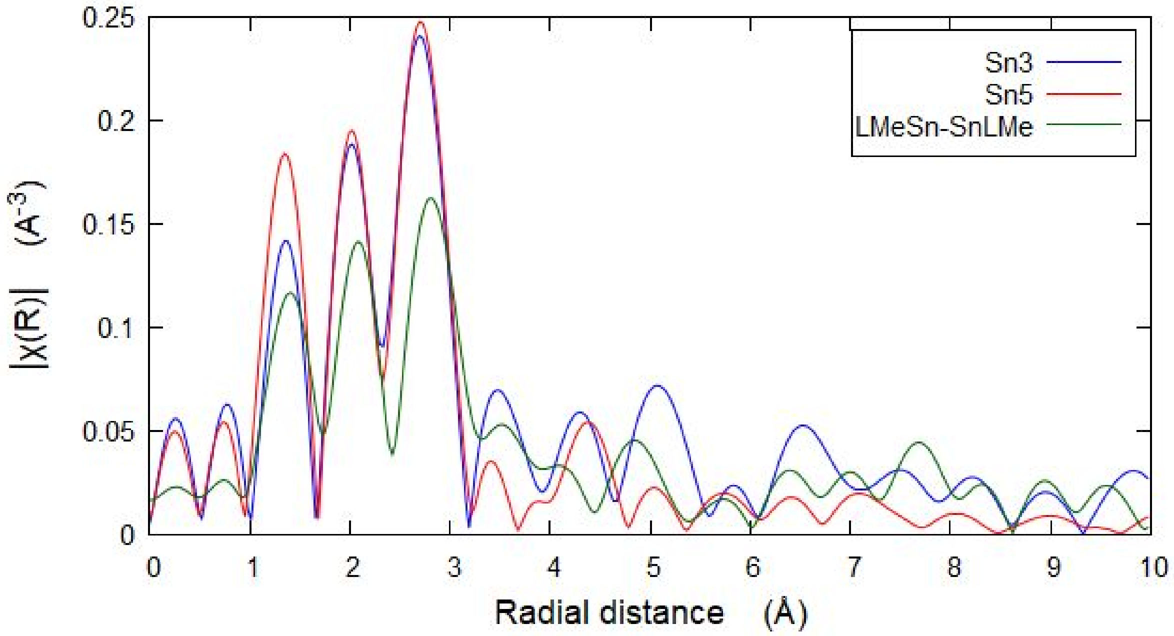

Figure S32. Solid-state Sn K-edge X-ray absorption spectra of tin complexes from this study and selected reference compounds: (a) normalized XANES spectra, (b) expanded XANES region, and (c) EXAFS spectra.

5. DFT calculations

Table S7. Comparison of selected bond lengths and bond angles between solid state structure and calculated structure of $L^{Me}SnSnL^{Me}$ (**Sn_2**)

Bond Parameters	Experimental (Crystal structure) data	Calculated structural Data
Sn(1) -Sn(2)	2.9537(4)Å	2.985 Å
$N_{Pyrrole}$(2)-Sn(1)	2.150(3)Å	2.162 Å
$N_{Pyrrole}$(5)-Sn(2)	2.135(3) Å	2.163 Å
$N_{Pyridine}$(1)-Sn(1)	2.446(3) Å	2.481 Å
$N_{Pyridine}$(4)-Sn(2)	2.469(3)Å	2.467 Å
$N_{Pyridine}$(1)-Sn(1)-Sn(2)	89.33(8)°	86.98°
$N_{Pyridine}$(4)-Sn(2)-Sn(1)	91.34(7)°	91.68°
$N_{Pyrrole}$(2)-Sn(1)-Sn(2)	96.10(8)°	93.84°
$N_{Pyrrole}$(5)-Sn(2)-Sn(1)	96.33(8)°	94.34°
$N_{Pyrrole}$(2)-Sn(1)-Sn(2)-$N_{pyrrole}$(5)	-52.67°	-50.63°

Table S8. Comparison of selected bond lengths and bond angles between solid state structure and calculated structure of $(L^{Ph}Sn)_3Cl$

Bond Parameters	Experimental (Crystal structure) data (Å) $(L^{Ph}Sn)_3Cl$	Calculated structural Data of $(L^{Ph}Sn)_3Cl$
$N_{pyrrole}$(2)–Sn(1)	2.151(4) Å	2.204 Å
$N_{pyrrole}$(5)–Sn(2)	2.129(4) Å	2.292 Å
$N_{pyrrole}$(8)–Sn(3)	2.152(5) Å	2.201 Å
Sn(1)–Sn(2)	2.8521(6) Å	2.868 Å
Sn(2)–Sn(3)	2.8632(6) Å	2.882 Å
Cl(1)–Sn(1)	3.557(1) Å	3.490 Å
Cl(1)–Sn(2)	3.231(2) Å	3.036 Å
Cl(1)–Sn(3)	3.713(2) Å	3.657 Å
Sn(1)–Sn(2)–Sn(3)	145.070(16)°	147.590°
$N_{pyrrole}$(2)–Sn(1)–Sn(2)	91.97(11)°	89.658°
$N_{pyrrole}$(5)–Sn(2)–Sn(1)	106.50(12)°	104.996°
$N_{pyrrole}$(5)–Sn(2)–Sn(3)	107.57(12)°	106.802°
$N_{pyrrole}$(8)–Sn(3)–Sn(2)	92.50(12)°	92.815°

Table S9. Comparison of selected bond lengths and bond angles between solid state structure and calculated structure of $L^{Ph}Sn$-$^{Ph}PDPSn$-SnL^{Ph}

Bond Parameters	Experimental (Crystal structure) data of $L^{Ph}Sn$-$^{Ph}PDPSn$-SnL^{Ph}	Calculated structural Data of $L^{Ph}Sn$-$^{Ph}PDPSn$-SnL^{Ph}
$N_{pyrrole}$(2)–Sn(1)	2.154(8) Å	2.181 Å
$N_{pyridine}$(5)–Sn(2)	2.232(7) Å	2.256 Å
$N_{pyrrole}$(8)–Sn(3)	2.232(7) Å	2.181 Å
Sn(1)–Sn(2)	2.945(8) Å	2.936 Å
Sn(2)–Sn(3)	2.960(8) Å	2.936 Å
N(2)-Sn(1)-N(1)	68.1(2)°	68.172°
N(8)-Sn(3)-N(9)	68.8(3)°	68.170°
N(5)-Sn(2)-N(4)	71.6(2)°	71.444°
Sn(1)–Sn(2)–Sn(3)	158.5(3)°	152.925°
$N_{pyrrole}$(2)–Sn(1)–Sn(2)	94.60(2)°	93.82°
$N_{pyridine}$(5)–Sn(2)–Sn(1)	98.42(16)°	103.538°
$N_{pyridine}$(5)–Sn(2)–Sn(3)	103.02(16)°	103.536°
$N_{pyrrole}$(8)–Sn(3)–Sn(2)	91.53(19)°	93.823°

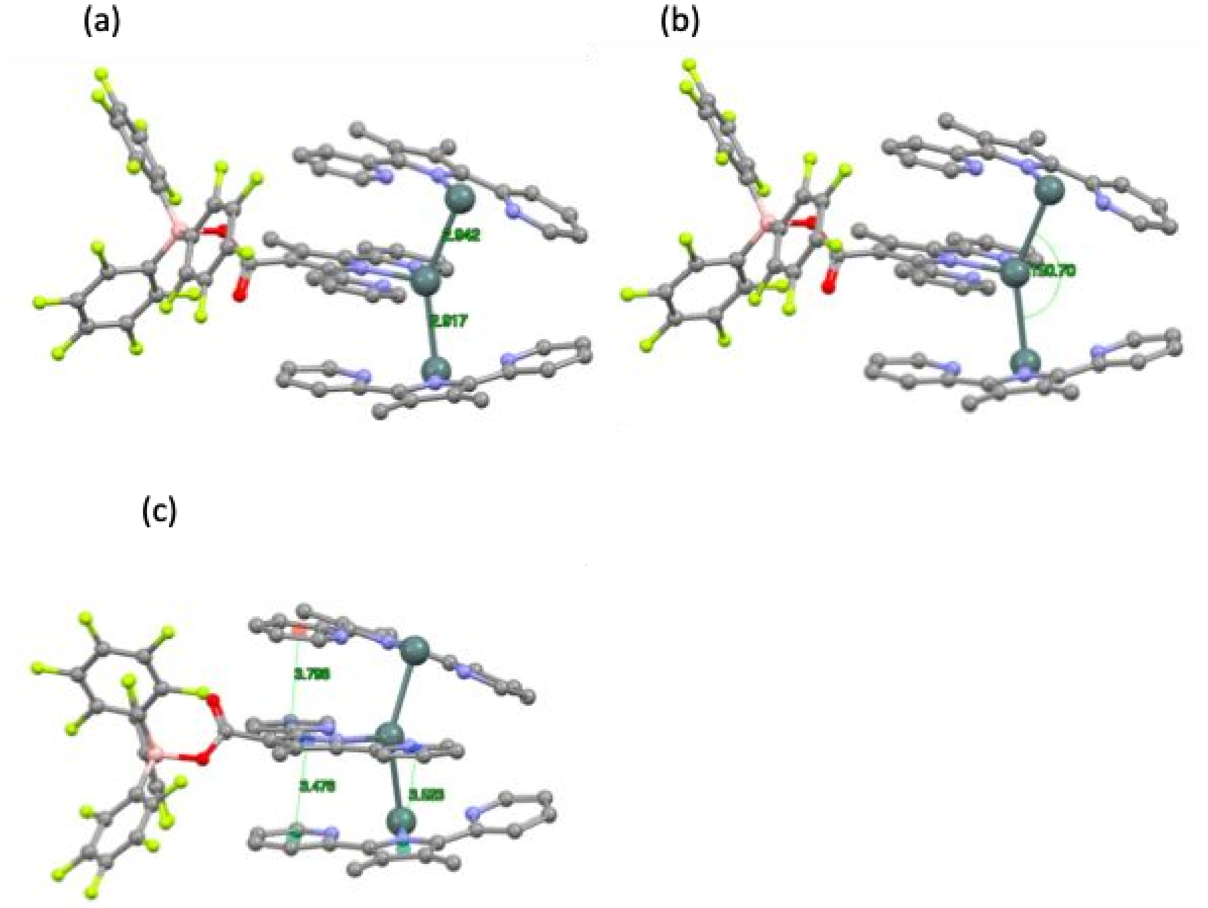

Figure S33. Optimized structure of Sn_3 with (a) Sn–Sn bond distances (Å), (b) bond angles (º), and (c) centroid–centroid distances between the pyridyl and pyrrolyl moieties. H atoms have been omitted for clarity.

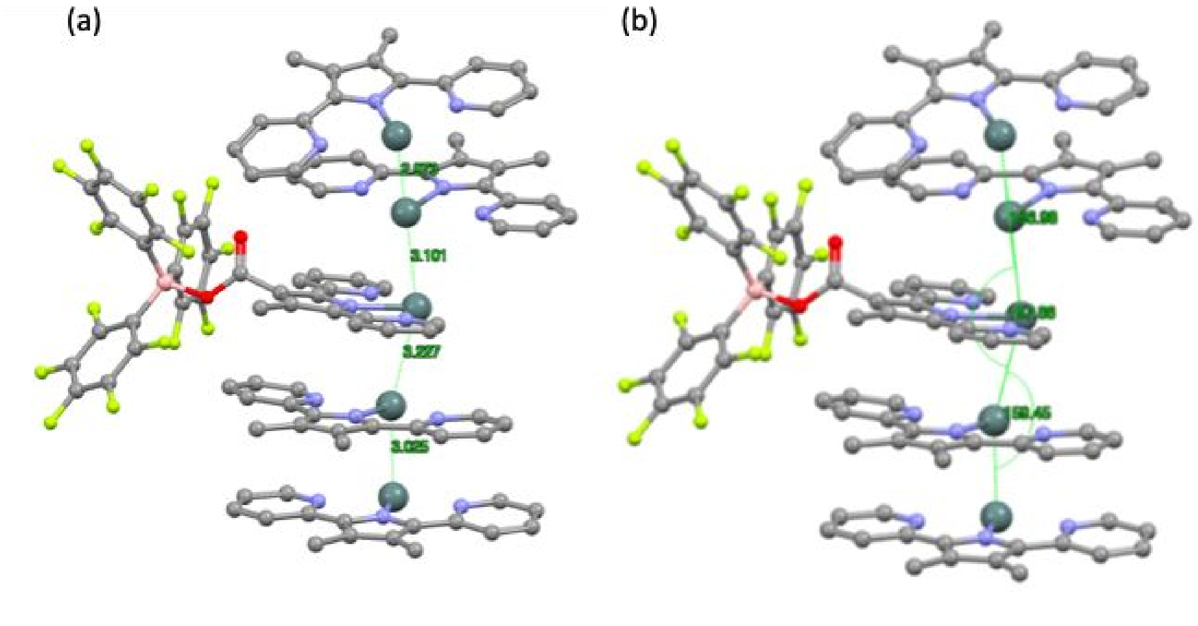

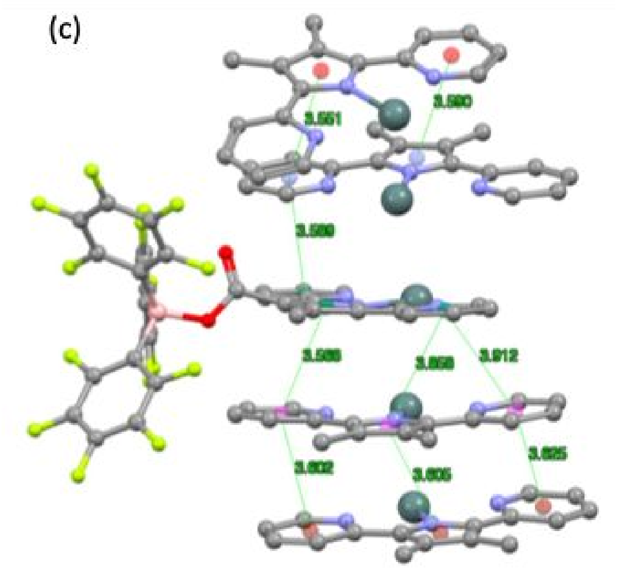

Figure S34. Optimized structure of Sn_5 with (a) Sn–Sn bond distances (Å), (b) bond angles (º), and (c) centroid–centroid distances between the pyridyl and pyrrolyl moieties. H atoms have been omitted for clarity.

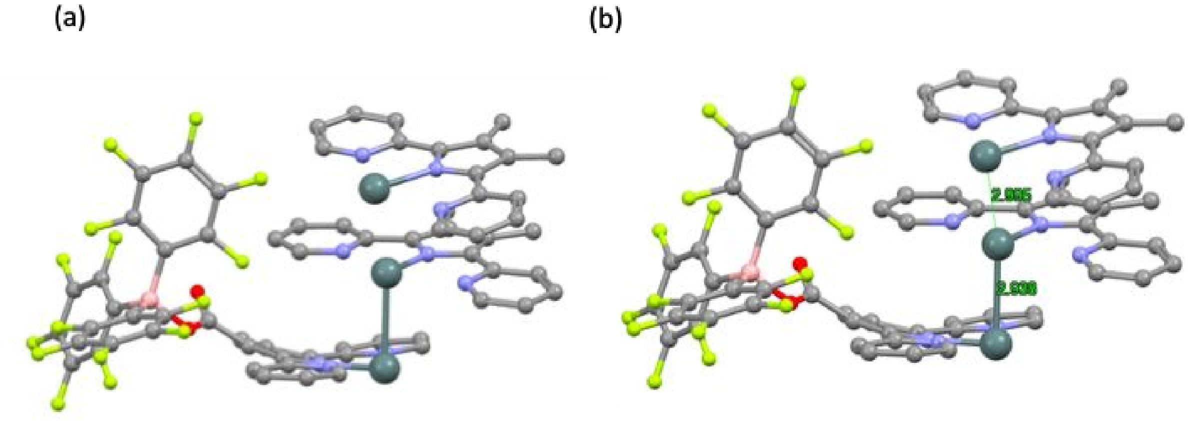

Figure S35. (a) Optimized structure of Sn_3_B1 with (b) Sn–Sn bond distances (Å). H atoms have been omitted for clarity.

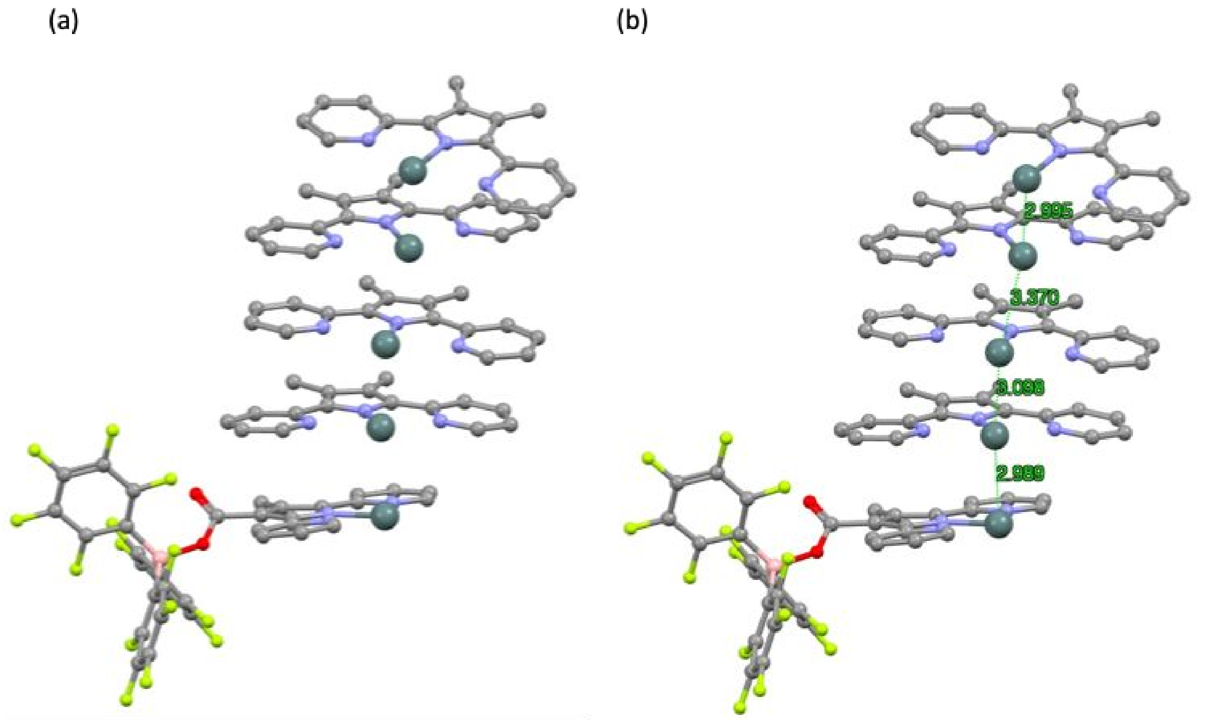

Figure S36. (a) Optimized structure of Sn_5_B1 with the (b) Sn–Sn bond distances (Å). H atoms have been omitted for clarity.

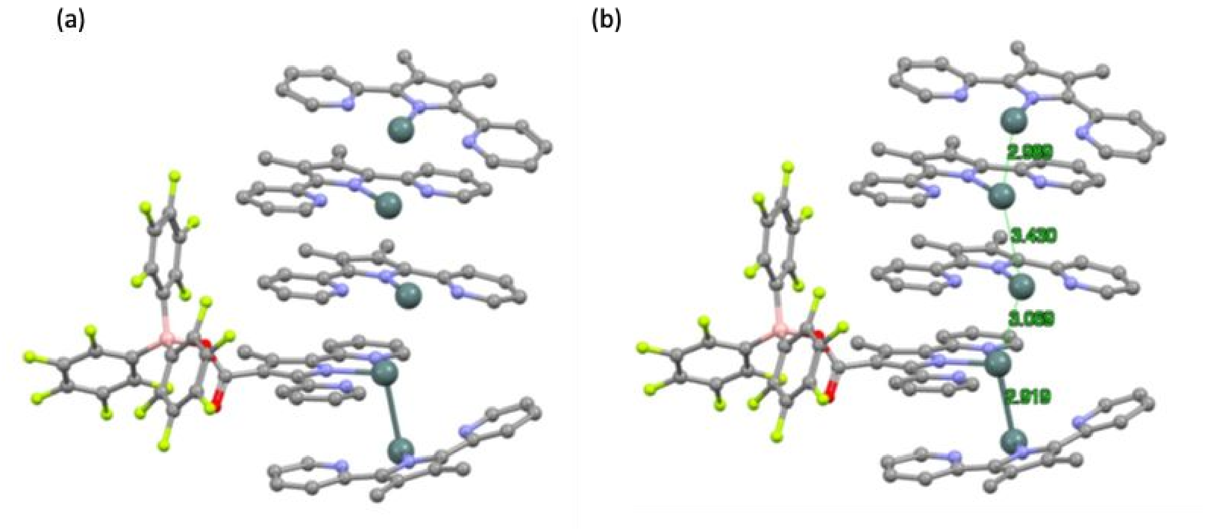

Figure S37. (a) Optimized structure of Sn_5_B2 with the (b) Sn–Sn bond distances (Å). H atoms have been omitted for clarity.
